# Strain and model development for auto- and heterotrophic 2,3-butanediol production using *Cupriavidus necator* H16

**DOI:** 10.1186/s13068-024-02549-7

**Published:** 2024-07-30

**Authors:** Janek R. Weiler, Nikolai Jürgensen, Monica Cornejo Infante, Melanie T. Knoll, Johannes Gescher

**Affiliations:** Institute of Technical Microbiology, Hamburg University of Technology, 21073 Hamburg, Germany

**Keywords:** *Cupriavidus necator* H16, *Ralstonia eutropha* H16, Autotrophic fermentation, Platform chemicals, 2,3-Butanediol

## Abstract

The production of platform chemicals from renewable energy sources is a crucial step towards a post-fossil economy. This study reports on the production of acetoin and 2,3-butanediol heterotrophically with fructose as substrate and autotrophically from CO_2_ as carbon source, H_2_ as electron donor and O_2_ as electron acceptor with *Cupriavidus necator.* In a previous study, the strain was developed for the production of acetoin with high carbon efficiency. Acetoin can serve as a precursor for the synthesis of 2,3-butanediol by the integration of a butanediol dehydrogenase. In this study, different plasmid backbones and butanediol dehydrogenases were evaluated regarding efficiency for CO_2_-based 2,3-butanediol production. The developed strain utilizes the pBBR1 plasmid bearing a 2,3-butanediol dehydrogenase from *Enterobacter cloacae* and is characterized by 2,3-butanediol as the main product and a heterotrophic total product yield of 88.11%, an autotrophic volumetric productivity of 39.45 mg L^−1^ h^−1^, a total product carbon yield of 81.6%, an H_2_ efficiency of 33.46%, and a specific productivity of 0.016 g product per gram of biomass per hour. In addition, a mathematical model was developed to simulate the processes under these conditions. With this model, it was possible to calculate productivities and substrate usage at distinct time points of the production processes and calculate productivities and substrate usage with high resolution which will be useful in future applications.

**Supplementary Information:**

The online version contains supplementary material available at 10.1186/s13068-024-02549-7.

## Introduction

To reduce the production of platform chemicals based on fossil fuels, it is crucial to develop new production techniques based on renewable and environmentally friendly carbon and energy sources. This study focuses on the engineering of *Cupriavidus necator* H16 (formerly *Ralstonia eutropha* H16) strains to produce 2,3-butanediol (2,3-BDO), a platform chemical with widespread industrial applications such as use as antifreeze, precursor for polymers or as a fuel additive [1, 2].

*C. necator*, a Gram-negative bacterium that has been intensively studied in recent decades, is recognized as a biocatalyst due to its ability to produce the biopolymer polyhydroxybutyrate (PHB) and to combine this with a chemolithoautotrophic metabolism. The bacterium is a non-pathogenic organism and the availability of its complete genome has also led to its recognition as a model organism. It can grow heterotrophically by utilizing various carbon sources and electron donors such as fructose, N-acetylglucosamine, gluconate, fatty acids and various other compounds. Under the conditions of chemolithoautotrophic growth, it is able to use oxygen, nitrate, nitrite or dimethyl sulfoxide (DMSO) as electron acceptors and carbon dioxide as a carbon source, with hydrogen acting as an electron donor [3, 4]. Previous research suggests that oxyhydrogen bacteria such as *C. necator* could be crucial for the future production of more complex carbon compounds [5]. The organism utilizes the Calvin cycle with its key enzyme ribulose-1,5-bisphosphate carboxylase/oxygenase (RuBisCO) for CO_2_ fixation. While an advantage of this process is that it directs carbon into glycolysis as a C3 compound, a disadvantage of this metabolic pathway is the undesirable oxygenase activity of RuBisCO, which requires a significant number of electrons for the reduction of oxygen instead of CO_2_, resulting in a lower yield of complex chemicals generated from metabolic intermediates. Carbonic anhydrases (CA) in *C. necator* serve as regulators of CO_2_ flux. They increase the amount of available CO_2_ in the cells and thus inhibit the oxygenase reaction of RuBisCO [6].

2,3-BDO can be produced from acetoin by utilizing the BudC enzyme (2,3-butanediol dehydrogenase) in an equilibrium reaction. Starting from a genetically modified *C. necator* strain introduced in 2019, this study aimed to expand production and investigate lithoautotrophic 2,3-BDO production. The original strain produced acetoin in minimal medium with over 100% carbon efficiency under autotrophic conditions [7] and was further analyzed and characterized by Härrer et al. in 2021 [8]. The study investigated the strain’s ability to utilize various organic acids and gases as substrates under mixotrophic and autotrophic conditions. In addition, a proteomic analysis was performed, which revealed that deletion of the *phaC1* and *phaC2* genes significantly influenced the carbon metabolism, resulting in higher carbon availability without the production of PHB. In addition, removal of the genes affected the transcription of *alsS* and *alsD*, the gene pair required for acetoin production from pyruvate, as they were placed under the control of a PHB promoter.

Various approaches for the microbial production of 2,3-BDO have been presented in scientific literature [1, 9]. Natural 2,3-BDO-producing bacterial strains such as *Klebsiella oxytoca* [10] or bacteria which are generally regarded as safe (GRAS) like *Paenibacillus peoriae* [11] and genetically engineered strains containing a 2,3-BDO metabolic pathway, such as *Escherichia coli* [12] and *Saccharomyces cerevisiae* [13], have shown carbon efficiencies of over 90% [mol mol^−1^]. These results demonstrate the potential of bacterial strains for 2,3-BDO production, but most cultured strains rely on heterotrophic carbon sources. Autotrophic production of butanediol has been observed in acetogens and cyanobacteria, but has not yet been fully exploited due to the complexity of strain engineering and process development. In a study by Köpke et al*.* [14], a concentration of 2 mM was reported for acetogenic BDO production as early as 2011. In contrast, a genetically modified strain of *Synechococcus elongatus* produced 26.4 mM 2,3-BDO from CO_2_ [15] with the disadvantage that, as a phototrophic organism, it requires a sufficient light source.

A number of modeling approaches have been developed in the literature to control and optimize PHB production processes of *C. necator* [16–18]. These models exist in different complexities and were applied to determine kinetics and process parameters of microbial growth and synthesis of PHB in batch and fed-batch operations based on differing and mixed substrate sources [17, 19]. In addition, these simulations were proposed for the optimization of substrate feeding strategies, evaluating the effects on growth and PHB production [20, 21]. One model focuses on the autotrophic process of PHB synthesis of *C. necator* considering the gas transfer and uptake of CO_2_, H_2_ and O_2_ [22]. Nevertheless, no modeling attempts have yet been made to describe the production of platform chemicals like 2,3-BDO. Still, a heterotrophic kinetic model of naturally 2,3-BDO-producing *K. oxytoca* exists, which describes cell growth and the production of 2,3-BDO under the influence of substrate and product inhibition in a batch fermentation [23].

The aim of this study was to establish and characterize the autotrophic production of 2,3-BDO using CO_2_ as carbon source and H_2_ as electron donor and energy source. We also aimed to generate a production strain that would set a new benchmark for autotrophic 2,3-BDO production. To achieve this goal, we investigated the effects of two production plasmids, pKR and pBBR1, as well as different versions of the BudC enzyme on productivity. In addition, we examined the influence of overexpression of a CA on autotrophic carbon efficiency. Furthermore, a mathematical batch process model was introduced for heterotrophic and autotrophic production of 2,3-BDO, consisting of mechanistic links describing the interaction of culture dynamics. The developed process models, estimation procedures and simulation results offer essential components for the future use of model-based automation concepts and effective user defined control of batch processes.

## Materials and methods

### Chemicals

All chemicals used in this study were supplied by Carl Roth (Karlsruhe, Germany) or Merck (Darmstadt, Germany). Gases were ordered at Westfalen (Münster, Germany).

### Strains and media

All strains and plasmids used in this study are listed in Supplement-Table S1.

*C. necator* strains were cultivated either in lysogeny broth (LB) or in minimal media (MM; German Collection of Microorganisms and Cell Cultures GmbH (DSMZ); media 81: solution C: 2.5 mg instead of 50 mg ferric ammonium citrate; additional 50 mg NaHCO_3_ at a temperature of 30 °C. When using minimal media, 20 mM fructose was added for heterotrophic growth. For autotrophic growth experiments a gas mixture of 80% H_2_, 5% CO_2_ and 15% O_2_ was added to the headspace. The cultures of *E. coli* strain WM3064 were grown in LB medium at 37 °C supplemented with 0.3 mM diaminopimelic acid (DAP). If required, kanamycin was added at a concentration of 250 μg ml^−1^ (stock solution 50 mg ml^−1^ in water) for *C. necator* strains and 50 µg ml^−1^ for *E. coli* strains or tetracycline at a concentration of 15 μg ml^−1^ (stock solution 15 mg ml^−1^ in DMSO). For growth on plates, the medium contained 2% agar. All media or stock solutions used were autoclaved at 121 °C for 20 min after preparation. Antibiotics, vitamin and fructose solutions were sterile filtered.

All experiments with explosive gas mixtures were carried out in specially designed fume cupboards with safety measures that allow the continuous release of the gas mixtures while maintaining explosion protection.

### Growth experiments

Heterotrophic growth curves of *C. necator* H16 WT were performed using an Infinite200Pro plate reader (Tecan Trading AG, Switzerland) over several days until stationary phase was reached. The precultures were grown overnight in LB at 30 °C and 180 rpm. Experiments were performed in triplicate in a 96-well plate at 30 °C and in MM containing different fructose and 2,3-BDO concentrations to determine possible substrate or product inhibition. Optical density at a wavelength of 600 nm (OD_600_) was measured hourly, with a 2-min pre-phase of shaking at 180 rpm, followed by a 1-min settling phase. The wells were filled with 200 µl of medium. The experiments were started with an OD_600_ value of 0.05.

### Production tests

The cells were pre-cultured overnight in LB and grown two nights in MM to shorten the adaptation lag phase in the production experiments. Heterotrophic experiments were inoculated at OD_600_ = 0.05 in 300 ml MM in sterile 500 ml flasks. Autotrophic experiments were inoculated at an OD_600_ = 4 in 50 ml MM in sterile, gas-tight 1-L bottles with rubber stoppers for sampling. The bottles were degassed in alternating 2-min cycles with vacuum and N_2_ addition at a pressure of 1 bar for 30 min. Vacuum was then applied for 20 min, after which the flasks were purged with the gas mixture for cultivation. The head space composition was then measured immediately. Samples of 1 mL were taken and analyzed for fructose and acetoin content by high performance liquid chromatography (HPLC). Gas chromatography (GC) was used to determine the 2,3-butanediol concentration. Samples for gas measurement were taken in a volume of 30 ml with a cannula through the rubber caps and immediately measured on a micro-GC. The pressure development caused by cultivation or sampling was then equalized to 1 bar by adding N_2_.

### Genetic construction

DNA was amplified with MangoMix (Bioline, Luckenwalde, Germany) for diagnostic polymerase chain reactions (PCRs) or with Hifi Polymerase (Nippon Genetics Europe, Düren, Germany) for preparative PCRs. The products were analyzed and isolated by gel electrophoresis. DNA products and plasmids were purified using the Wizard^®^SV Gel and PCR Clean-Up System or the Wizard^®^Plus SV Minipreps DNA Purification System (Promega, Mannheim, Germany). Restriction enzymes were provided by New England Biolabs (Frankfurt, Germany), while DNA primers were ordered from Merck (Darmstadt, Germany). The constructs were sequenced by Eurofins Genomics (Ebersberg, Germany) and aligned to the expected sequences with the help of the molecular biology tools of the software Benchling (San Francisco, USA).

The plasmids were constructed according to Windhorst and Gescher (2019) using the natural PHB promoter system, the acetoin-producing genes *alsS* and *alsD* from *Bacillus subtilis* PY79 and the 2,3-butanediol-producing BudC from *Klebsiella pneumoniae*, *Klebsiella aerogenes or Enterobacter cloacae*. In addition, a *cag* gene from *C. necator* was used to overexpress a carbonic anhydrase. The *budC* genes were optimized for codon usage of *C. necator* H16 ([24]; please see details on codon optimization in the Appendix) and synthesized as DNA strings by Thermo Fisher Scientific (Waltham, USA). If possible, the ordered strings already had the necessary overlaps to the respective plasmids. PCR copies of the strings were generated by preparative PCR with overlaps, if necessary, and cloned into the respective plasmid using Gibson assembly [25]. The generated DNA overlap sequences contained non-coding sequences as spacers between the genes containing a Shine–Dalgarno sequence. The primers, restriction enzymes and generated plasmids used can be found in Table S1. The Gibson mixture was dialyzed for 30 min and transformed into *E.* coli strain WM3064 by electrophoresis. Once a strain carrying the correct plasmid was identified, it was used as the donor strain for conjugation with the *C. necator* H16 strain JG1232. Donor and recipient strains were streaked overnight on LB plates with DAP and then on plates with the respective antibiotic but without DAP. After 3 days of incubation, the colonies were tested for the correct plasmid using diagnostic PCR and then sent for sequencing.

### Substrate and product analysis

#### High-performance liquid chromatography

HPLC measurements were performed using a Thermo Scientific^™^ UltiMate^™^ 3000 UHPLC system from Thermo Fisher Scientific (Waltham, USA). 150 μl of samples was filtered with a 0.2 μm PTFE membrane (VWR, Darmstadt, Germany) and mixed with 15 μl of 0.5 M sulfuric acid in a 96-well microtiter plate. Samples were separated using a Hi-Plex H PL1170-6830 (7.7 × 300 mm) 8 μm HPLC column from Agilent Technologies (Waldbronn, Germany) at an eluent flow rate of 0.6 ml min^−1^. The refractive index peaks were detected using a RefractoMax 521 detector from Thermo Fisher Scientific. The eluent used was 5 mM sulfuric acid in ddH_2_O, and the column temperature was 60 °C during the measurement. The results were analyzed using Chromeleon 7.2 SR4 software.

#### Gas chromatography

GC measurements were performed with a Shimadzu GC-2000 Plus equipped with a split/splitless injector system (AOC 20 s Auto Sampler) and a flame ionization detector (Det3ch Shimadzu, Kyoto, Japan). Helium was used as the carrier gas with a total flow rate of 22.7 ml min^−1^. The injection port was preheated to 230 °C in split mode with a split ratio of 1:10. Samples were separated using an Agilent CP-Chirasil-Dex CB column (25 m × 0.25 mm) with a stationary film thickness of 0.25 μm. The column temperature was initially set to 50 °C for 3 min and then increased by 10 °C min^−1^ until it reached 160 °C. The final temperature was maintained for 5 min. The temperature at the injection port and at the FID was constant at 230 °C.

#### Micro-GC

Gas chromatography was used to measure the concentrations of H_2_, O_2_, N_2_, and CO_2_. The measurements were performed with the 490 Micro-GC from Agilent Technologies (Waldbronn, Germany) and analyzed with the Agilent OpenLAB CDS (EZChrom Edition) software. The method included a stabilization time of 5 s, a sample time of 20 s, an injector time of 50 ms and a temperature of 110 °C between sample line and injector. The first column was a 10-m MS5A column with argon as carrier gas at a temperature of 70 °C and a pressure of 150 kPa. The second column was a 10-m PPQ column with helium as carrier gas at a temperature of 45 °C and a pressure of 150 kPa.

### Calculation of biomass and efficiencies

#### Determination of biomass

OD_600_ was measured using a Thermo Fisher Scientific Spectronic Genesys 20 Visible Spectrophotometer (Darmstadt, Germany). Sample biomass calculations were performed using a conversion factor from OD_600_ to dry biomass derived from the correlation of dry mass and OD_600_ of the samples in a growth experiment (Eq. 1).

The conversion factor was used as follows:

1 $$Dry\,Biomass\,[g\,{\text{L}}^{{ - {1}}} ]\, = \,0.{6416} \cdot OD_{{{6}00}} \, + \,0.0{632}$$

#### Biomass yield

The biomass yield coefficient was determined using the following equation (Eq. 2) in which Δ*c*_*x*_ is the biomass concentration produced, Δ*c*_*s*_ is the substrate consumption:

2 $$Yield\,Y_{bmass} \left[ {\frac{g\,biomass}{{g\,substrate}}} \right]\, = \,\frac{{\Delta c_{x} }}{{\Delta c_{s} }}\, = \,\frac{{c_{x,end} \, - \,c_{x,0} }}{{ c_{s,end} \, - \,c_{s,0} }}$$

where x is the biomass determined, s is the substrate, Δ*cx* [g L^−1^] is the biomass produced and Δ*cs* [g L^−1^] is the substrate consumption.

To facilitate comparison with the carbon yield, the percentage of substrate moles used by the cells for biomass production was also calculated (Eq. 3). For this purpose, the mole numbers for biomass were approximated, assuming that the simplified chemical composition of biomass is C_4_H_7_O_1.5_N [26, 27], resulting in a molecular weight of 93 g mol^−1^:

3 $$Biomass \,Yield\,Y_{X/S} \,\left[ \% \right]\, = \,\frac{{\Delta n_{x} }}{{\Delta n_{s} }}\, * \,100$$

where Δn_x_ [mol L^−1^] is the biomass produced as a molar concentration, calculated from Δ*c*_*x*_ and the assumed molecular weight of biomass. Δn_s_ [mol L^−1^] is the mass concentration of fructose consumption determined in the experiments.

### Specific growth rate

The specific growth rate was calculated in exponential growth phase over 10 h as follows (Eq. 4):

4 $$specific \,growth\,rate\,\mu \left[ {h^{ - 1} } \right]\, = \,\frac{change\,in\,measured\,optical\,density}{{period\,of\,time}}\, = \,\frac{{{\text{ln}}\left( {OD_{600, 1} } \right)\, - \,{\text{ln}}\left( {OD_{600} } \right)}}{{ t_{1} \, - \,t_{0} }}$$

where the natural logarithm values of OD_0_ and OD_1_ are used for the time points t_0_ and t_1_, respectively.

### Carbon yield under heterotrophic conditions

The product carbon yield can be calculated using the following equation (Eq. 5):

5 $$Yield\,Y_{het} \left[ \% \right]\, = \,\frac{{product\,\left[ {mM} \right]}}{{converted\,substrate\,\left[ {mM} \right]}}\, = \,\frac{{n_{product, end} \, - \,n_{product, t0} }}{{n_{substrate,t0} \, - \,n_{substrate, end} }}\, = \,\frac{{\left( {\left[ {product in mM} \right]_{End} \, - \,\left[ {product in mM} \right]_{0} } \right)\, * \,V_{liq} }}{{n_{substrate,t0} \, - \,n_{substrate, end} }}$$

Two moles of pyruvate can be formed for each mole of fructose. They can then be converted by catalysis into one mole of acetoin/2,3-BDO and two CO_2_ which consequently are lost for the heterotrophic production process. If every fructose molecule is used for acetoin production, this results in a maximum theoretical yield of 1 mol acetoin per mol fructose which was which was, therefore, calculated to be 100%. If all the acetoin is converted to 2,3-BDO, the maximum theoretical carbon yield for 2,3-BDO is, therefore, also 100%.

The volume of the liquid phase V_liq_ was affected by each sampling and was, therefore, calculated by subtracting the volume of the extracted samples from the initial liquid volume. For each sample, the amount of substance extracted was calculated and added to the amount of final products.

### Carbon yield under autotrophic conditions

The product carbon yield under autotrophic conditions can be calculated using the following equation (Eq. 6):

6 $$\begin{gathered} Yield\,Y_{aut} \left[ {\frac{mM\,product}{{mM\,substrate}}} \right]\, = \,\frac{produced\,product}{{converted\,substrate}}\, = \,\frac{{n_{product, end} \, - \,n_{product,t0} }}{{n_{C, 0} \, - \,n_{C, end} }} \hfill \\ \quad = \frac{{\left( {\left[ {Product\,in\,mM} \right]_{end} \, - \,\left[ {Product\,in\,mM} \right]_{0} } \right)\,*V_{liq} }}{{n_{C, t0} \, - \,n_{C, end} }} \hfill \\ \end{gathered}$$

where C is the carbon available as a substrate from CO_2_ and NaHCO_3_.

The volume of the liquid phase *V*_*liq*_ was affected by each sampling step and was, therefore, calculated by subtracting the volume of samples taken from the initial liquid volume. In addition, the extracted moles were calculated for each sample and added to the final product volume.

The total moles of the gas mixture in the gas phase *n*_*gas*_ were determined using the ideal gas law. Using the ideal gas constant *R* = 8.314 *JK*∙*mol*, considering a gas volume of *V*_*gas*_ = 1.08 L in the flasks and the ambient temperature (T = 298.15 K) and pressure (P = 1 bar = 100 kPa) when sampling, the gas moles were determined as follows (Eq. 7):

7 $$n_{gas} \, = \,\frac{{P*V_{gas} }}{R*T}\, = \,\frac{{100000 Pa*0.00108 m^{3} }}{{8.314 \frac{J}{K}mol*298.15 K}}\, = \,0.0435 mol\, = \,43.5 mmol$$

The quantity of each component in the gas mixture was determined by multiplying the partial volume p_*i*_ [%] by the total moles of gas calculated above (Eq. 9):

9 $$n_{i} \, = \,p_{i} *n_{gas}$$

Under autotrophic conditions, 6 mol of CO_2_ are required to form 2 mol of pyruvate. These two pyruvate molecules can then be further converted to 1 mol of acetoin/2,3-BDO and 2 mol of CO_2_. Simply put, for every mole of acetoin or 2,3-BDO, 4 mol of CO_2_ is consumed resulting in a maximum yield of 25%. For comparison, if all of the CO_2_ is used to produce acetoin or 2,3-BDO, the yield was calculated to be 100%. The gas samples taken at the relevant times were taken into consideration as with *V*_*liq*_ above, while CO_2_ and NaHCO_3_ were considered as substrate/available carbon.

### Hydrogen efficiency

The hydrogen efficiency is an equivalent of how much energy is consumed in carbon fixation for the products. It can be calculated as follows (Eq. 10):

10 $$H_{2\,eff} \left[ \% \right]\, = \,\frac{{n_{{H_{2\,theoretical} }} }}{{n_{{H_{2\,consumed} }} }}*100$$

In order to determine the theoretical amount of hydrogen ${n}_{{H}_{2}}$ [mmol] required, the amount of bound carbon was determined, and the number of electrons required to get from the substrate to the product was calculated.

### Mathematical process model

A kinetic model was used to simulate the heterotrophic and autotrophic experimental data of this work. Model structures were applied to the experimental dataset of each genetically modified *C. necator* strain and adapted individually depending on the process observations. This model provides a simple structure for heterotrophic and autotrophic simulation describing cell growth using the Monod kinetic, synthesis of acetoin and 2,3-BDO as well as equilibrium reactions and the uptake and transfer of gases for the autotrophic process.

### Heterotrophic process model

The heterotrophic set up consists of 4 differential equations (Eq. 11, 12, 13, 14) and 8 kinetic rates which are summarized in Table 1. The model is describing the growth rate $\mu$ [h^−1^] of the cells X [g_cell_ L^−1^] based on the Monod kinetic and the main substrate fructose ${c}_{S,F}$ [g L^−1^] including also a substrate affinity constant ${K}_{S,F}$ [g L^−1^] (Eq. 15). A cell lysis term was added including a minimal and a maximal death rate with ${\mu }_{d,min}$ [h^−1^] and ${\mu }_{d,max}$ [h^−1^] (Eq. 19). To describe a delay of growth or production in time ${t}_{lag}$ [h] a lag term was added as exponential function (Eqs. 15, 16) [28]. In case of a product inhibition of 2,3-BDO an inhibition term is considered affecting the growth kinetic and cell lysis via a product inhibition constant ${K}_{I,B}$ [g L^−1^] (Eq. 16, 20) [52]. The specific uptake rate of fructose ${q}_{S,F}$ [g g_cell_^−1^ h^−1^] was described by the growth rate of cells and the yield coefficient of biomass from substrate uptake ${Y}_{X,F}$ [g_cell_ g^−1^] (Eq. 17). The specific production rates for acetoin ${q}_{P,A}$ [g g_cell_^−1^ h^−1^] and 2,3-BDO ${q}_{P,B}$ [g g_cell_^−1^ h^−1^] were calculated from their linked yield coefficients of cells to acetoin ${Y}_{X,A}$ [g_cell_ g^−1^] and to 2,3-BDO production ${Y}_{X,B}$ [g_cell_ g^−1^] (Eqs. 18, 21). In case of a substrate depletion, the backward reaction from 2,3-BDO to acetoin was considered as observed in the experimental data (Eq. 22), where ${q}_{B,max}$ [g g_cell_^−1^ h^−1^] is the maximum uptake rate of 2,3-BDO as ${c}_{P,B}$ [g L^−1^] with an affinity constant ${K}_{P,B}$ [g L^−1^] and a yield coefficient of acetoin from 2,3-BDO ${Y}_{A,B}$ [g g^−1^].

**Table 1:** Heterotrophic process model in batch operation

Differential equations			
$\frac{dX}{dt}=(\mu -{\mu }_{d})\cdot X$	(Eq. 11)	$\frac{d{c}_{P,A}}{dt}={q}_{P,A}\cdot X$	(Eq. 13)
$\frac{d{c}_{S,F}}{dt}=-{q}_{S,F}\cdot X$	(Eq. 12)	$\frac{d{c}_{P,B}}{dt}={q}_{P,B}\cdot X$	(Eq. 14)
Kinetic rates			
$\mu = {\mu }_{max}\cdot \frac{{c}_{S,F}}{{c}_{S,F}+{K}_{S,F}}\cdot (1-{e}^{\frac{-t}{{t}_{lag}}})$	(Eq. 15)	${\mu }_{d}= {\mu }_{d,min}+{\mu }_{d,max}\cdot \frac{{K}_{S,F}}{{K}_{S,F}+{c}_{S,F}}$	(Eq. 19)
$\mu = {\mu }_{max}\cdot \frac{{c}_{S,F}}{{c}_{S,F}+{K}_{S,F}}\cdot (1-{e}^{\frac{-t}{{t}_{lag}}})\cdot \frac{{K}_{I,B}}{{K}_{I,B}+{c}_{P,B}}$	(Eq. 16)	${\mu }_{d}= {\mu }_{d,min}+{\mu }_{d,max}\cdot \frac{{K}_{S,F}}{{K}_{S,F}+{c}_{S,F}}\cdot \frac{{c}_{P,B}}{{c}_{P,B}+{K}_{I,B}}$	(Eq. 20)
${q}_{S,F}= \frac{\mu }{{Y}_{X,F}}$	(Eq. 17)	${q}_{P,B}= \frac{\mu }{{Y}_{X,B}}$	(Eq. 21)
${q}_{P,A}= \frac{\mu }{{Y}_{X,A}}$	(Eq. 18)	${q}_{P,AB}={Y}_{A,B}\cdot {q}_{B,max}\bullet \frac{{c}_{P,B}}{{c}_{P,B}+{K}_{P,B}}$	(Eq. 22)

### Autotrophic process model

The autotrophic process model follows a similar structure and set of equations as the heterotrophic model. It is summarized in Table 2 and consists of 9 differential equations (Eqs. 23, 24, 25, 26, 27, 28, 29, 30, 31) and 15 kinetic rates considering the uptake and transfer of gases. The dissolved gas concentrations of CO_2_, O_2_ and H_2_ as ${c}_{{CO}_{2},l}$, ${c}_{{O}_{2},l}$, and ${c}_{{H}_{2},l}$ [mol L^−1^] were calculated from the gas transfer rate and the gas uptake rate, respectively (Eqs. 29, 30, 31), by a mass transfer coefficient ${k}_{L}a$ [h^−1^], the equilibrium concentrations ${c}_{{O}_{2}}^{*}$, ${c}_{{CO}_{2}}^{*}$, and ${c}_{{H}_{2}}^{*}$ [mol L^−1^] and by considering a yield coefficient to gas uptake as ${Y}_{X,{O}_{2}}$, ${Y}_{X,{CO}_{2}}, \text{and} {Y}_{X,{H}_{2}}$ [g_cell_ mol^−1^]. Therefore, Henry constants ${H}_{{O}_{2}}$, ${H}_{C{O}_{2}}$, and ${H}_{{H}_{2}}$[mol L^−1^ atm^−1^] were calculated at 30 °C [29] to describe the gas solubilities ${c}_{{O}_{2}}^{*}$, ${c}_{{CO}_{2}}^{*}$, and ${c}_{{H}_{2}}^{*}$ at atmospheric pressure $P$ [atm] with their partial pressure in the gas phase ${c}_{{O}_{2},g}$, ${c}_{C{O}_{2},g}, \text{and} {c}_{{H}_{2},g}$ (Eqs. 42, 43, 44). The partial pressures were calculated from the gas transfer rate, the liquid volume ${V}_{liq}$ [L], the gas volume ${V}_{gas}$ [L], $R$ as the ideal gas constant [8.314 J mol^−1^ K^−1^] and $T$ the temperature [303.15 K] (Eqs. 26, 27, 28). The growth rate and cell lysis are based on the availability of the dissolved gases with their related affinity constant ${K}_{{O}_{2}}$, ${K}_{{CO}_{2}}$, and ${K}_{{H}_{2}}$ [mol L^−1^] (Eqs. 32, 33). To describe further depletion of gases if one gas is already consumed, the kinetic rates were switched (Eqs. 40, 41). The first and second forward reaction rates from acetoin to 2,3-BDO were described by a maximum uptake rate of acetoin ${q}_{f1,A,max}$, ${q}_{f2,A,max}$ [g g_cell_^−1^ h^−1^], an affinity constant${K}_{f1,A}$, ${K}_{f2,A}$ [g L^−1^] and a yield coefficient${Y}_{f1,B,A}$, ${Y}_{f2,B,A}$ [g g^−1^] (Eqs. 36, 38, 45, 46). A reversed reaction from 2,3-BDO to acetoin was conversely described by a maximum uptake rate of 2,3-BDO ${q}_{r,B,max}$ [g g_cell_^−1^ h^−1^], an affinity constant, ${K}_{r,B}$ [g L^−1^] and a yield coefficient ${Y}_{r,A,B}$ [g g^−1^] (Eqs. 37, 39). These specific rate equations (Eqs. 36, 37, 38, 39, 45, 46) were implemented in the differential equations (Eqs. 24, 25) on demand as an individual model structure for each data set.

**Table 2:** Autotrophic process model in batch operation

Differential equations			
$\frac{dX}{dt}=(\mu -{\mu }_{d})\cdot X$	(Eq. 23)	$\frac{d{c}_{{H}_{2},g}}{dt}=-{k}_{L}a\cdot ({c}_{{H}_{2}}^{*}-{c}_{{H}_{2},l})\cdot \frac{{V}_{L}\cdot R\cdot T}{{V}_{G}\cdot P}$	(Eq. 28)
$\frac{d{c}_{P,A}}{dt}={q}_{P,A}\cdot X$	(Eq. 24)	$\frac{d{c}_{{O}_{2},l}}{dt}={k}_{L}a\cdot \left({c}_{{O}_{2}}^{*}-{c}_{{O}_{2},l}\right)-X\frac{\mu }{{Y}_{X,{O}_{2}}}$	(Eq. 29)
$\frac{d{c}_{P,B}}{dt}={q}_{P,B}\cdot X$	(Eq. 25)	$\frac{d{c}_{{CO}_{2},l}}{dt}={k}_{L}a\cdot \left({c}_{C{O}_{2}}^{*}-{c}_{C{O}_{2},l}\right)-X\frac{\mu }{{Y}_{X,{CO}_{2}}}$	(Eq. 30)
$\frac{d{c}_{{O}_{2},g}}{dt}=-{k}_{L}a\cdot ({c}_{{O}_{2}}^{*}-{c}_{{O}_{2},l})\cdot \frac{{V}_{L}\cdot R\cdot T}{{V}_{G}\cdot P}$	(Eq. 26)	$\frac{d{c}_{{H}_{2},l}}{dt}={k}_{L}a\cdot \left({c}_{{H}_{2}}^{*}-{c}_{{H}_{2},l}\right)-X\frac{\mu }{{Y}_{X,{H}_{2}}}$	(Eq. 31)
$$\frac{d{c}_{{CO}_{2},g}}{dt}=-{k}_{L}a\cdot ({c}_{{CO}_{2}}^{*}-{c}_{C{O}_{2},l})\cdot \frac{{V}_{L}\cdot R\cdot T}{{V}_{G}\cdot P}$$	(Eq. 27)		
Kinetic rates			
$\mu = {\mu }_{max}\cdot \frac{{c}_{{O}_{2},l}}{{c}_{{O}_{2},l}+{K}_{{O}_{2}}}\cdot \frac{{c}_{C{O}_{2},l}}{{c}_{{CO}_{2},l}+{K}_{C{O}_{2}}}\cdot \frac{{c}_{{H}_{2},l}}{{c}_{{H}_{2},l}+{K}_{{H}_{2}}}$	(Eq. 32)	${\mu }_{switch}= {\mu }_{max}\cdot \frac{{c}_{{O}_{2},l}}{{c}_{{O}_{2},l}+{K}_{{O}_{2}}}\cdot \frac{{c}_{{H}_{2},l}}{{c}_{{H}_{2},l}+{K}_{{H}_{2}}}$	(Eq. 40)
${\mu }_{d}= {\mu }_{d,min}+{\mu }_{d,max}\cdot \frac{{c}_{{O}_{2},l}}{{c}_{{O}_{2},l}+{K}_{{O}_{2}}}\cdot \frac{{c}_{C{O}_{2},l}}{{c}_{{CO}_{2},l}+{K}_{C{O}_{2}}}\cdot \frac{{c}_{{H}_{2},l}}{{c}_{{H}_{2},l}+{K}_{{H}_{2}}}$	(Eq. 33)	${\mu }_{d,switch}= {\mu }_{d,min}+{\mu }_{d,max}\cdot \frac{{c}_{{O}_{2},l}}{{c}_{{O}_{2},l}+{K}_{{O}_{2}}}\cdot \frac{{c}_{{H}_{2},l}}{{c}_{{H}_{2},l}+{K}_{{H}_{2}}}$	(Eq. 41)
${q}_{P,A}= \frac{\mu }{{Y}_{X,A}}$	(Eq. 34)	${c}_{{O}_{2}}^{*}={c}_{{O}_{2},g}\cdot P\bullet {H}_{{O}_{2}}$	(Eq. 42)
${q}_{P,B}= \frac{\mu }{{Y}_{X,B}}$	(Eq. 35)	${c}_{C{O}_{2}}^{*}={c}_{C{O}_{2},g}\cdot P\cdot {H}_{C{O}_{2}}$	(Eq. 43)
${q}_{f1,A}={q}_{f1,A,max}\cdot \frac{{c}_{P,A}}{{c}_{P,A}+{K}_{f1,A}}$	(Eq. 36)	${c}_{{H}_{2}}^{*}={c}_{{H}_{2},g}\cdot P\cdot {H}_{{H}_{2}}$	(Eq. 44)
${q}_{r,B}={q}_{r,B,max}\cdot \frac{{c}_{P,B}}{{c}_{P,B}+{K}_{r,B}}$	(Eq. 37)	${q}_{f2,A}={q}_{f2,A,max}\cdot \frac{{c}_{P,A}}{{c}_{P,A}+{K}_{f2,A}}$	(Eq. 45)
${q}_{f1,BA}={Y}_{f1,B,A}\cdot {q}_{f1,A}$	(Eq. 38)	${q}_{f2,BA}={Y}_{f2,B,A}\cdot {q}_{f2,A}$	(Eq. 46)
$${q}_{r,AB}={Y}_{r,A,B}\cdot {q}_{r,B}$$	(Eq. 39)		

### Parameter estimation

The individual model structure and associated procedures for simulating processes and estimating parameters were implemented in Matlab (The MathWorks, Inc., USA, Version 2020b). Parameter estimation was achieved by embedding the interior-point optimization algorithm using the built-in solver “fmincon” in Matlab. To consider further parameter bounds, upper and lower limits were applied with a maximum of 3000 function evaluations and 200 iterations. The implemented ordinary differential–algebraic equations were solved using the ode15s algorithm based on numerical differentiation formulas for stiff systems. In order to obtain the best possible fit, the sum of least squared errors between simulated and experimental data was determined as objective function. As quality criteria for the validation of the simulated results, the coefficient of determination, R^2^, was applied to evaluate the ratio of the squared differences between the experimental data ${y}_{i}$ and the simulated data ${y}_{s}$ as well as the squared differences between the experimental data and the mean value $\overline{y}$ (Eq. 47) [30]. The model accuracy is higher as closer the coefficient of determination is to 1 [31]:

47 $${\text{R}}^{2} {\mkern 1mu} = {\mkern 1mu} 1{\mkern 1mu} - {\mkern 1mu} \frac{{\sum\nolimits_{{{\text{ i = 1}}}}^{{{\text{ n}}}} {\left( {{\text{y}}_{{\text{i}}} {\mkern 1mu} - {\mkern 1mu} {\text{y}}_{{\text{s}}} } \right)^{2} } }}{{\sum\nolimits_{{{\text{ i = 1}}}}^{{{\text{ n}}}} {\left( {{\text{y}}_{{\text{i}}} {\mkern 1mu} - {\mkern 1mu} \overline{{\text{y}}} } \right)^{2} } }}$$

During each iteration, the calculated differences between the measured and predicted values were summarized by an objective function. The unknown model parameters were then adjusted iteratively until error tolerances or convergence criteria were met and the best model fit was achieved.

## Results

To achieve a productive and stable autotrophic production of the platform chemical 2,3-BDO, different versions of the production gene and various production plasmids were investigated in this study. In addition, an endogenous CA was overexpressed to optimize autotrophic carbon efficiency. An existing strain was used as a starting point to facilitate the production of butanediol and increase productivity and efficiency [7].

### Tolerance growth tests

**Fig. 1:**
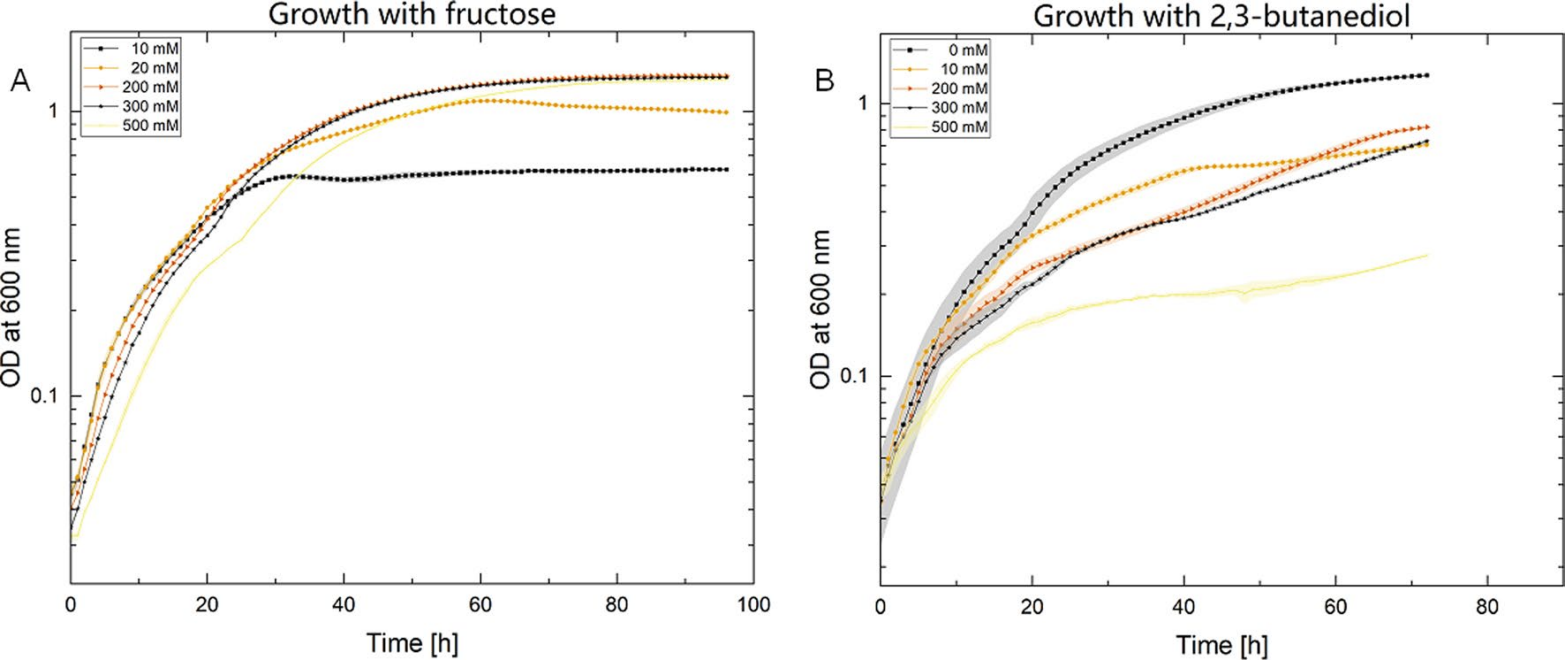
Growth experiments were conducted with *C. necator* H16 WT using increasing concentrations of fructose (**A**) and 2,3-BDO (**B**) over 72 and 98 h. Triplicates are indicated by the surrounding color clouds. The datasets were reduced for a better distinction between the sets. The full datasets can be seen in Appendix A figure S1

In order to establish the production of 2,3-BDO, the ability of the organism to tolerate the product had to be tested. Therefore, heterotrophic assays were performed to assess the organism’s tolerance to the substrate and product using growth experiments with a *C. necator* H16 wild-type strain in a medium containing increasing concentrations of fructose and 2,3-BDO, respectively, from 0 to 500 mM. The corresponding data can be seen in Fig. 1, which shows only a reduced amount of tested concentrations for visibility. The full dataset can be found in Appendix A figure S1. No significant effect on cell growth was observed up to 400 mM fructose over a period of 72–98 h, with cells reaching a maximum growth of OD_600_ = 1.41 (Fig. 1A). Concentrations of 450 and 500 mM resulted in a slightly prolonged growth period until plateau phase was reached with a 10-h delay. The cells metabolized lower concentrations of 10 and 20 mM fructose after 30 and 60 h, respectively, and maintained a stable plateau of optical density during the remaining measurements. All strains showed a comparable specific growth rate between 0.049 and 0.059 h^−1^ during the exponential phase. On the other hand, cell growth was already impaired by concentrations of 2,3-BDO above 10 mM (Fig. 1B). All concentrations up to 450 mM showed a comparable pattern with a maximum optical density of 0.6–0.8 and a specific growth rate between 0.014 and 0.024 h^−1^. In contrast, the culture grown with 500 mM of 2,3-BDO showed a maximum optical density of 0.25 after 72 h and a growth rate of *µ* = 0.01 h^−1^.

### Heterotrophic production tests

**Fig. 2:**
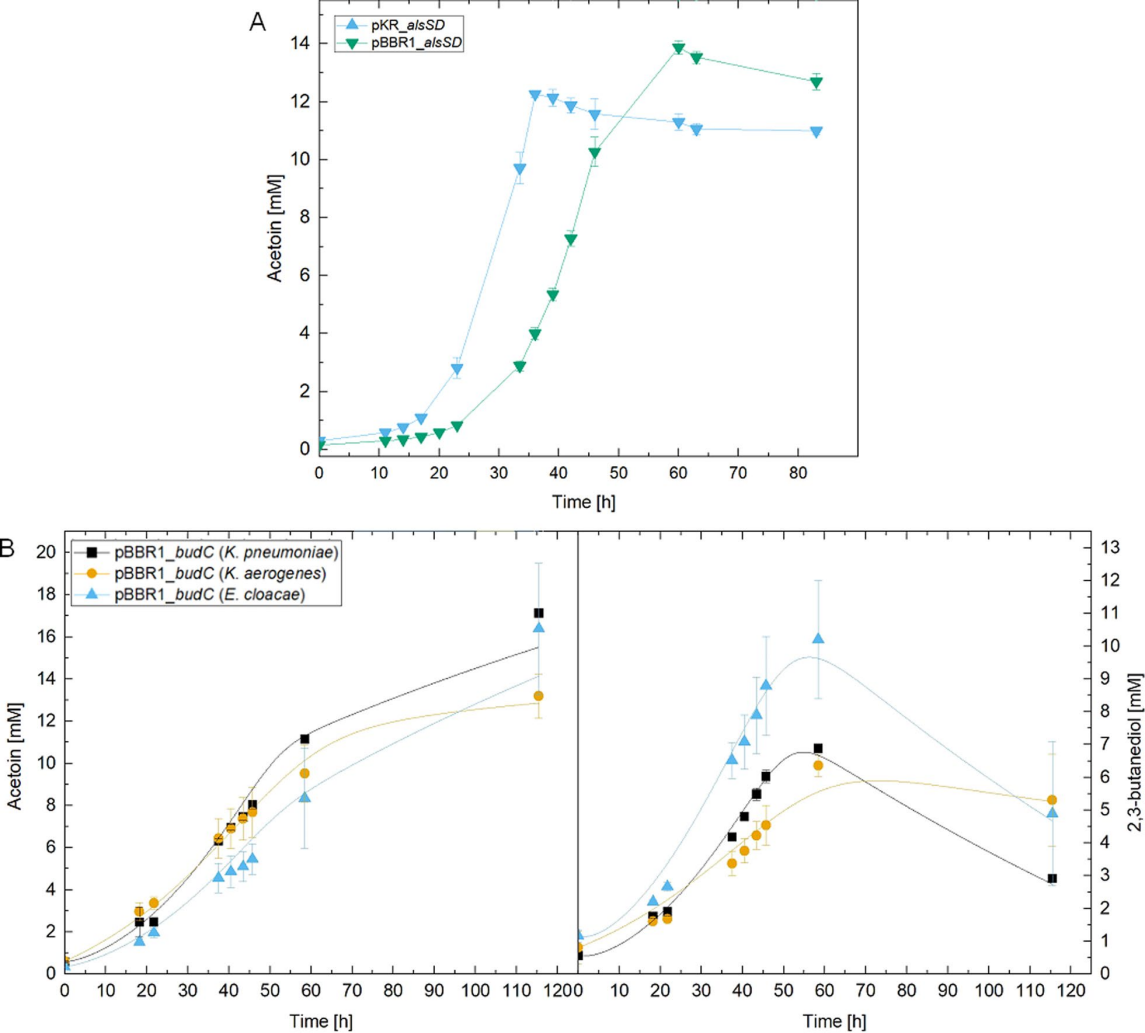
Comparison of production plasmids (**A**) and variants of *budC* from different organisms (**B**; organism of origin specified in parentheses) under heterotrophic conditions. Acetoin and 2,3-BDO values are displayed, while Figures S1 (for **A**) and S2 (for **B**) contain optical density and fructose measurements. The lines in the lower graph represent the simulated, while the symbols indicate measured results of the experiments

After determining the growth of the organisms in production tests, two plasmids, both carrying identical *alsSD* genes, were tested regarding the resulting acetoin production under heterotrophic conditions. The better performing plasmid was then selected and three versions of the *budC* sequence from different organisms were compared to determine the most effective variant in terms of volumetric productivity, carbon efficiency and acetoin/2,3-BDO ratio (Fig. 2). The donor organisms were selected to be *Klebsiella pneumoniae*, *Klebsiella aerogenes* (basionym: *Enterobacter aerogenes*) and *Enterobacter cloacae* as they are all known to be efficient 2,3-butanediol producers and were used in various approaches in the past [32–34].

Comparison of the plasmids pKR and pBBR1 revealed that while the pKR plasmid led to a faster response, the overall titer for acetoin was higher for pBBR1 (Fig. 2A). Hence, further experiments to compare different versions of the *budC* gene were conducted using the pBBR1 plasmid. Of the three variants, *budC* (*K. pneumoniae*) showed the highest acetoin accumulation, while *budC* (*E. cloacae*) showed the highest amount of 2,3-BDO at 10.2 mM (Fig. 2B). The results of the comparisons and of the applied heterotrophic model simulation for acetoin and 2,3-BDO are depicted in Fig. 2. The fit for *budC* (*K. pneumoniae*) showed the best predicted outcome of 15.5 mM acetoin, demonstrating a *R*^2^ value of 0.98 and a 2,3-BDO accumulation of 6.8 mM with a *R*^2^ of 0.99. In comparison, simulations of *budC* (*K. aerogenes*) and *budC* (*E. cloacae*) provided an acetoin production of 12.8 and 14.1 mM, while 2,3-BDO amounted to 5.9 and 9.6 mM, respectively. Each showed a *R*^2^ value for acetoin of 0.99 and 0.96, whereas for 2,3-BDO, the model demonstrated a *R*^2^ value of 0.95 and 0.98. The measured and simulated optical densities and fructose concentrations are shown in Figures S1 and S2. An overview of the R^2^ values and fitted parameters are summarized in Tables S2 and S4.

After fructose depletion, all strains apparently started to reoxidize the produced 2,3-BDO to acetoin, potentially to stabilize membrane potential using the regenerated NADH as electron donor for the respiratory transport chain towards oxygen (Fig. 2B). This reverse reaction was also included in the model structure and shows a decline of 2,3-BDO amounts, while acetoin concentrations are increasing.

In terms of carbon efficiency and biomass yield of the experiments, *budC* (*E. cloacae*) demonstrated the highest carbon yield for 2,3-BDO and the highest total product yield with 88.11%. In addition, among the pBBR1 constructs, it displayed the lowest biomass- and acetoin carbon yield. Among all constructs, pBBR1_*budC* (*K. pneumoniae*) exhibited the highest acetoin yield, while *budC* (*K. aerogenes*) had the highest biomass yield (Table 3).

**Table 3:** Carbon yields of the strains in heterotrophic production experiments presented as percentages of carbon derived from fructose as well as biomass yields and total product yields

*budC* sequence from	Biomass yield [g g^−1^]	Acetoin yield [%]	2,3-butanediol yield [%]	Total product yield [%]
*K. pneumoniae* (pKR)	0.23 ± 0.01	25.96 ± 0.00	10.8 ± 0.01	36.74 ± 0.01
*K. pneumoniae* (pBBR1)	0.075 ± 0.01	54.45 ± 0.00	32.55 ± 0.01	87.00 ± 0.01
*K. aerogenes*	0.078 ± 0.00	52.48 ± 0.09	32.70 ± 0.05	85.19 ± 0.14
*E. cloacae*	0.064 ± 0.01	41.34 ± 0.07	46.77 ± 0.1	88.11 ± 0.05

### Autotrophic production tests

Potentially, the most interesting applications for production strains of oxyhydrogen bacteria are both mixotrophic and autotrophic. Therefore, the best performing constructs were tested in autotrophic efficiency experiments and compared to the heterotrophic results to determine comparability.

**Fig. 3:**
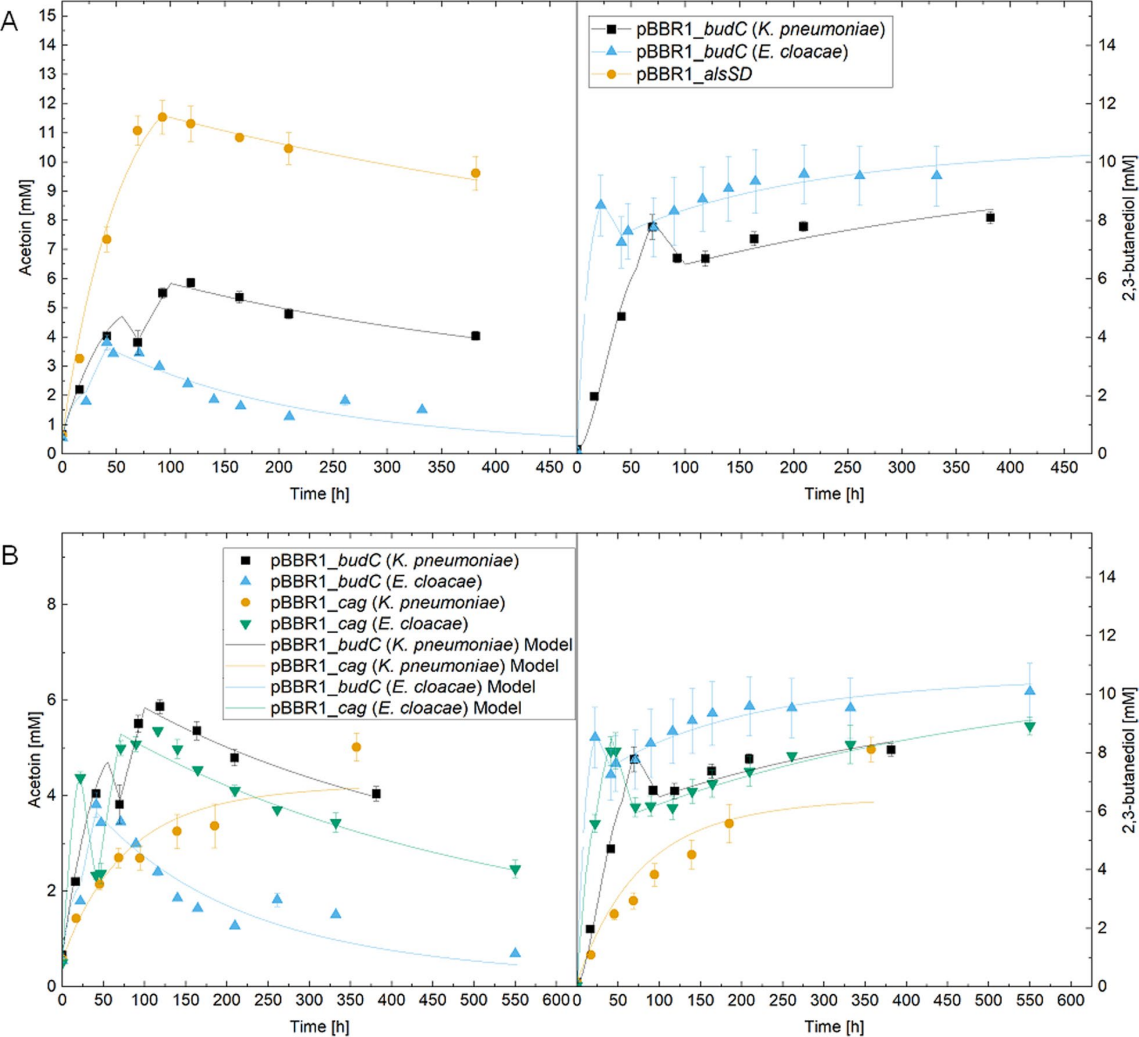
Comparing *budC* variations from different organisms (**A**; as indicated by organism names in parentheses) and strains carrying a CA gene under autotrophic conditions (**B**). Each case displays acetoin and 2,3-BDO values, while optical density and gas measurements can be found in Figures S3–S5. The lines represent the simulated, while the symbols show measured experimental results

Autotrophic experiments were performed as cell suspension assays with an OD_600_ = 4 (Fig. 3). For comparison purposes, the two best heterotrophic versions of *budC* were selected for the autotrophic test (Fig. 3A). In direct comparison, cells with *budC* (*E. cloacae*) produced up to 8.5 mM 2.3-BDO and 1.8 mM acetoin within 22 h. Conversely, the strain with *budC* (*K. pneumoniae*) accumulated a maximum of 7.8 mM 2,3-BDO and 3.8 mM acetoin after 70 h. The simulations predicted an accumulated 2,3-BDO amount of 8.5 mM and acetoin of 2.1 mM during the first 22 h with an *R*^2^ value of 0.99 and 0.90, respectively for *budC* (*E. cloacae*). The maximum of 2,3-BDO with 10.3 mM was reached at the end of the simulation. Simulation results of *budC* (*K. pneumoniae)* showed a 2,3-BDO concentration of 7.9 mM after 70 h and a maximum of 5.8 mM acetoin after 100 h, demonstrating a *R*^2^ of 0.99 and 0.99, respectively. The highest 2,3-BDO concentration for this strain was calculated after 380 h at 8.4 mM.

To potentially improve carbon uptake in the production strains, a naturally occurring CA (*cag*) was integrated into the plasmids which has been hypothesized to be responsible to supplement CO_2_ to the RuBisCO directly [6]. For comparison, Fig. 3B shows a side-by-side comparison of the two strains. The *budC*-bearing strain (*E. cloacae*) with *cag* overexpression showed similar behavior to the original strain, with lower 2,3-BDO values but higher acetoin accumulations. After 47 h, the strain reached 8 mM 2,3-BDO, with a maximum concentration of 8.9 mM, and a maximum concentration of 5.4 mM acetoin after 116 h. In comparison the autotrophic simulation results for *cag* (*E. cloacae*) showed 8 mM of 2,3-BDO at 47 h, with a maximum of 9.1 mM at the end and 5.3 mM of acetoin after 72 h. The coefficient of determination resulted in 0.99 and 0.97 for 2,3-BDO and acetoin, respectively. A maximum 2,3-BDO concentration of 6.3 mM and acetoin of 4.1 mM after 360 h was reached by *cag* (*K. pneumoniae*), with R^2^ values of 0.90 and 0.88 each. The autotrophic simulated optical densities and gas concentrations are shown in Figures S4 and S5. An overview of the R^2^ values and fitted parameters are summarized in Tables S3 and S5.

**Fig. 4:**
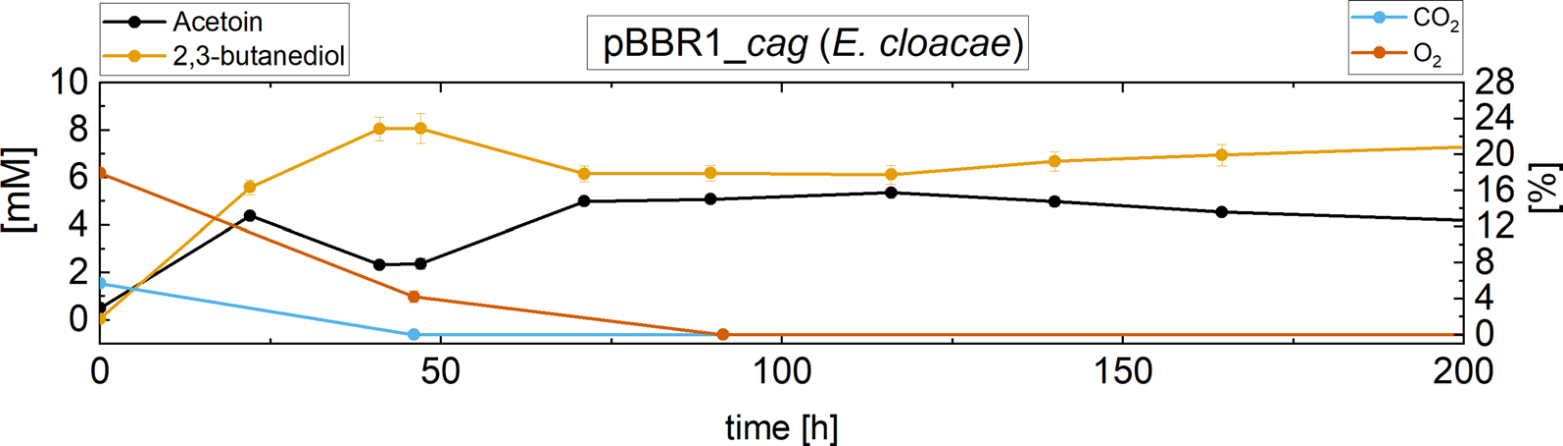
Exemplary demonstration of the catalyzed back-reaction from 2,3-butanediol to acetoin under autotrophic conditions of pBBR1PHB_*cag* (*E. cloacae*). Furthermore, CO_2_ and O_2_ measurements are presented to display the correlation between substrate availability and the enzyme’s catalytic reactions

The overexpression of *cag* in the strain containing the *budC* gene of *K. pneumoniae* led to a decrease overall and a more than threefold reduction in the consumption rate of gaseous substrates. Only after 357 h, the production levels were similar to those of the original strain. All strains carrying *budC* versions showed a comparable reverse reaction of 2,3-BDO to acetoin as in the heterotrophic experiments after depletion of the carbon source. However, the reverse reaction stopped after oxygen as electron acceptor was depleted (as shown for one strain in Fig. 4). Subsequently, the reverse reaction to 2,3-BDO slowly resumed. These observed effects were incorporated and simulated with an individual model structure for each strain.

The carbon and hydrogen yields were determined for the tested strains (see Table 4). Moreover, the resulting volumetric productivities were calculated. The lack of back-reaction to acetoin resulted in significantly higher yields for 2,3-BDO and a more favorable ratio between the products compared to the heterotrophic experiments. Both strains carrying the *budC* version of *E. cloacae* exhibited a higher carbon yield for 2,3-BDO than their counterparts carrying the *K. pneumoniae* version, but a lower yield for acetoin. In addition, *budC* (*E. cloacae*) showed the highest carbon yield for 2,3-BDO, the lowest yield for acetoin and the highest volumetric productivity of 39.45 mg L^−1^ h^−1^ among all strains, which is consistent with previous results (compare Table 3). Strain *cag* (*K. pneumoniae*) had the lowest volumetric productivity value of all strains at 9.37 mg L^−1^ h^−1^. However, it had the highest hydrogen and total product carbon yield of all strains and the highest acetoin carbon yield of the *budC* strains. For comparison, the simulated volumetric productivities are depicted for each autotrophic strain and displayed in Table 5 at the same time points as measurements. In addition, the calculated maximum volumetric productivities at different time points occurring in between sampling points are displayed.

**Table 4:** Carbon yields from production assays under autotrophic conditions using strains carrying the pBBR1 plasmid as well as total hydrogen efficiencies and the H_2_ to CO_2_ ratios

Strain	Acetoin yield [%]	2,3-butanediol yield [%]	Total product yield [%]	Hydrogen efficiency [%]	H_2_/CO_2_*
*budC* (*E. cloacae*)	8.68 ± 0.4	72.92 ± 3.8	81.6 ± 4.2	33.46 ± 0.67	6.62
*cag* (*E. cloacae*)	18.36 ± 1	66.4 ± 3.8	84.76 ± 4.8	31.68 ± 4.37	7.11
*budC* (*K. pneumoniae*)	27.52 ± 1.6	61.16 ± 3.2	88.68 ± 4.8	36 ± 2.08	6.63
*cag* (*K. pneumoniae*)	35.04 ± 2.6	62 ± 3.4	97.04 ± 6	41.1 ± 2.57	6.62
pBBR1_*alsSD*	71.72 ± 4		71.72 ± 4	38.55 ± 2.84	7.00

* + NaHCO_3_

**Table 5:** Measured and calculated volumetric productivities of the strains at autotrophic productions

Vol. productivity [mg L^−1^ h^−1^] at first sample point							Simulated maximum	
Strain	Acetoin	2,3-BDO	Total	Acetoin_Simulated_	2,3-BDO_Simulated_	Total_Simulated_	Acetoin_Simulated_	2,3-BDO_Simulated_
*budC* (*K. pneumoniae*)	8.45	10.21	18.67	9.50	7.75	17.25	10.79	10.82
*cag* (*K. pneumoniae*)	4.45	4.92	9.37	3.67	6.46	10.13	4.09	7.19
*budC* (*E. cloacae*)	4.97	34.48	39.45	6.33	34.53	40.86	14.44	78.78
*cag* (*E. cloacae*)	15.52	22.70	38.23	15.82	22.75	38.57	26.25	37.74
*alsSD* (*B. subtilis*)	14.50	–	14.50	19.01	–	19.01	21.81	–

Volumetric productivities were measured at the first sampling point and compared with the correlating model calculation. In addition, the maximum simulated values were added

## Discussion

There is a necessity for alternatives to petroleum-based chemical production. In particular, utilizing waste streams is a promising approach to reduce the total amount of waste, the energy required for its disposal and the environmental damage. One of the most challenging waste streams is CO_2_ from industrial processing. To utilize it effectively, stable mixotrophic and autotrophic productions are essential to develop a viable alternative to petrochemical processing.

To assess the potential applicability of *C*. *necator* for long-term production of 2,3-BDO resistance to substrate and product was investigated in this study. 2,3-BDO has been shown to inhibit the growth of certain organisms, which necessitated these resistance tests. The results showed a clear range of optimal performance. Fructose had no significant effect on growth in the concentrations tested, while 2,3-BDO showed an effect between 10 and 450 mM, finally inhibiting growth almost completely at 500 mM, which is a comparable value to studies with other organisms [14, 35]. The following investigation focused on the autotrophic production of 2,3-BDO using different plasmid and gene combinations, based on the study by Windhorst and Gescher from 2019, in which a remarkably efficient strain for the autotrophic production of acetoin, a precursor of 2,3-BDO, was presented. To evaluate the efficiency of production, two production plasmids with identical gene sets were compared. These comparative studies clearly showed the correlation between slow growth and fast production and vice versa, especially in the plasmid comparisons. The differences in productivity could be due to differences in plasmid size, antibiotic resistance or variations in plasmid copy number. pBBR1, which is characterized by smaller size and kanamycin resistance, has been documented as an intermediate copy number plasmid, whereas differing copy numbers have been reported for pKR. As strain heterogeneity significantly affects the copy numbers of different plasmids, as opposed to the widely accepted static amounts [36], it is difficult to determine which, if not all, of the aforementioned differences are responsible although copy number appears to be the most obvious factor at first sight since it directly influences the number of the production genes [37]. The choice of antibiotic resistance can significantly affect productivity due to their different underlying mechanisms. For example, kanamycin resistance is due to an aminoglycoside phosphotransferase that catalyzes phosphorylation of the antibiotic depleting it over time, while energy-dependent efflux pumps enable tetracycline resistance. Still, it is worth noting that this antibiotic can form complexes with various iron ions which will also lead to fluctuating levels of selection pressure during the experimental period [38]. For 2,3-BDO production experiments, pBBR1 was chosen because it is robust and easy to use and the catalysis of fructose to acetoin was faster and more efficient. All strains with *budC* variants proved to be viable in the heterotrophic experiments. To ensure comparability of strain productivity, the amount of products was calculated in relation to the accumulated biomass. The two strains with the best carbon efficiency and specific productivity, carrying *budC* variations of *E. cloacae* and *K. pneumoniae*, showed maximum values of 0.108 and 0.114 g product per g biomass per hour, which were then further investigated in production experiments under autotrophic conditions.

High carbon efficiencies ranging from 81 to 97% were achieved in all 2,3-BDO strains during the experiments. The *E. cloacae budC* variant had the highest butanediol accumulation with a volumetric productivity of 39.45 mg L^−1^ h^−1^. However, its specific productivity per biomass was relatively low compared to heterotrophic experiments. Strains with *budC* (*K. pneumoniae*) produced yields ranging from 0.005 to 0.009, while *E. cloacae* variants yielded 0.015 to 0.016 g of product per g of biomass per hour.

Comparison with prior studies based on *C. necator* is partially limited, as they show lower volumetric productivities but produce larger, more complex end products [39], or indicate similar efficiencies but higher productivities in flow-through set-ups with a larger time scale, where maximum productivity is only reached after several hundred hours and substrate is not limited [40]. Other comparisons with natural producers of 2,3-BDO, which are capable of producing 2,3-BDO autotrophically, are complex since the experiments here were conducted as cell suspension assays. This stands in contrast to typical other studies where productivity was assessed during growth of the organisms. Nevertheless, some cyanobacteria and acetogenic bacteria can naturally produce 2,3-BDO autotrophically such as the above-mentioned cyanobacterium *S. elongatus* which reached a concentration of 5.5 mM of 2,3-BDO after 72 h showing a volumetric productivity of 6.9 mg L^−1^ h^−1^ [41]. This is a considerably lower concentration compared to the 8.5 mM of 2.3-BDO which were produced within a 22 h timeframe by the here developed *C. necator* strain. Although a long-term production study has not yet been conducted, the experiments presented here also indicate that the top-performing strain has a significantly higher maximum volumetric productivity of 34.5 mg 2,3-BDO L^−1^ h^−1^. Some acetogenic bacteria are well known for the autotrophic production of 2,3-BDO and have been the focus of many publications and optimization studies. Acetogenic bacteria produce acetate and ethanol in large quantities and 2,3-BDO as a side product. Hence, many optimization studies revolve around shifting the end product towards a higher 2,3-BDO yield involving (I) addition of cysteine leading to a 2,3-BDO concentration of 28.6 mM with CO_2_ and H_2_ as substrates [42], (II) optimizing gas composition and adding CO as electron donor which increased the yield of 2,3-BDO in a fed-batch fermentation up to 188 mM [43] or (III) adding zinc and iron to the medium resulting in 22 mM of 2,3-BDO and a volumetric productivity of 38 mg L^−1^ h^−1^ [44]. Although it will be necessary to show in scalable fermentation studies the robustness and long-term productivity of the developed *C. necator* strain, similar autotrophic productivities compared to acetogens were reached and 2,3-BDO was the main fermentation end product.

The ability to utilize syngas as a substrate is a significant advantage for acetogens due to their capacity to consume gas blends containing high levels of carbon monoxide (CO) which is used as electron donor. On the downside, the hydrogenases of acetogenic bacteria are sensitive to CO which hampers the use of H_2_ and CO at the same time at too high levels rendering it necessary to first utilize one and then the other [45]. Interestingly, the hydrogenases of C. necator were shown to be insensitive to CO [46, 47]. Naturally, *C. necator* lacks the essential carbon monoxide dehydrogenase to utilize CO but researchers could show that an engineered strain was able to utilize CO as substrate [4]. In addition, through laboratory evolution two critical mutations that enhance CO-tolerance of the organism were identified [48], providing the potential for engineering a viable strain that can use CO as a substrate with an increased resistance to its toxic effects. Alternatively, with aforementioned mutations, the microbe could also be used to clean CO in off-gas mixtures through consumption of H_2_ and CO_2_, thereby furnishing it as a reducer for various industrial carbonylation reactions in chemical production or catalytic processes [49].

The hydrogen efficiencies were generally comparable to those of other studies with *C. necator* [7, 40] but were inferior to acetogens that are commonly utilized in autotrophic production. Natural acetogenic producers of 2,3-BDO typically achieve a hydrogen yield of around 90% including side products. However, the production of 2,3-BDO is limited to low concentrations and yields high amounts of byproducts such as acetate and ethanol [44]. Subsequently, these efficiencies might be misleading because the process requires upcycling of acetate to a final end product in a second step, resulting in a significant decrease in overall efficiency.

The addition of the CA gene *cag* did not lead to an increase in efficiencies. Although the strains showed slightly higher efficiencies, the differences were not statistically significant, as indicated by a two-tailed t-test, with *p* values of 0.36 for the strains bearing *budC* (*E*. *cloacae*) and 0.16 for the strains carrying *budC* (*K*. *pneumoniae*), respectively. Still, four different versions of CAs are present in *C. necator* H16, each having different roles in the CO_2_ flux, ranging from pH regulation to bicarbonate supply for metabolism and CO_2_ supply to the RuBisCO enzyme. Hence, the concerted concentration of all four CAs might have to be carefully adjusted to benefit from their catalysis regarding carbon and hydrogen efficiency [6].

The back-reaction of butanediol to acetoin is a remarkable process that regulates NAD(P)H concentration and is necessary in wild-type strains for the utilization of butanediol/acetoin as a carbon source [3, 9]. Nevertheless, the deletion of the *acoABC* genes blocked further consumption of acetoin. Still the reaction from 2,3-BDO to acetoin was observed in all strains after the carbon source was depleted, but ceases in the autotrophic assays once the electron acceptor is no longer present. Hence, using continuous production, it seems possible to efficiently suppress this unfavorable reaction.

The strains that grew at a faster rate demonstrated lower efficiencies, whereas those with slower growth exhibited higher values. This seems to indicate a direct relation between substrate uptake, metabolism and production efficiencies. Within these lines, future studies will focus on upscaling and feeding strategies for continuous processes. Especially, feeding regimes have been shown in previous publications to be crucial for optimizing heterotrophic as well as autotrophic 2,3-BDO production [44, 50].

The heterotrophic batch process model based on mass balances as differential equations was able to mimic the production of acetoin and 2,3-BDO based on fructose consumption and biomass growth for 3 heterotrophic tested strains. The incorporated model and proposed rate structures are similar as described in Möller et al. [51]. The simulations for *budC* (*K. pneumoniae)*, *budC* (*K. aerogenes)*, and *budC* (*E. cloacae)* showed overall good fitting results. However, the biomass accumulations were overestimated after the exponential growth phase until the substrate amount was too low and the effect of the lysis kinetic took place. The simulated acetoin values agreed well with the experimental data, up to the last measured point where their amount was predicted to be lower. This is due to the proposed reverse reaction of 2,3-BDO to acetoin as soon as the substrate is almost depleted, since the decreasing 2,3-BDO concentrations were calculated to be converted with a fitted theoretical yield coefficient around 0.97 g g^−1^. However, the suggested product inhibition function [52] had no influence on the simulation results as the concentrations of 2,3-BDO were below the level of inhibition observed in the growth experiments at 10 mM, which could be the reason for an overestimated biomass simulation as the inhibitions influence is uncertain between 0 and 10 mM. The potential of autotrophic compared to heterotrophic 2,3-BDO production implies a CO_2_ reduction, whereas the biomass growth rate is usually less, similar to an autotrophic model describing PHB production with *C. necator* [22]. The developed autotrophic model was capable of predicting uptake, transfer of gases and the production of acetoin and 2,3-BDO well based on their calculated coefficient of determination and is so far the only model for this process. However, the experimentally obtained data indicated variations in the reaction rates of autotrophic strains, necessitating individual adaptations of the model structures. For strain *budC* (*K. pneumoniae*), a forward reaction of acetoin to 2,3-BDO was considered when CO_2_ was almost depleted and a backward reaction occurred afterwards as long as enough O_2_ was present. As soon as the two gases were consumed, the forward reaction appeared again. Those mathematically described effects were also integrated for *cag* (*E. cloacae*). Conversely, the model structure for *budC* (*E. cloacae)* does not incorporate the first forward reaction. For *cag* (*K. pneumoniae*), only the last forward reaction was considered, since the gases were not completely depleted. In case one, gas is depleted during the fermentation, the kinetic for the growth rate is switched, to describe an ongoing depletion of the present gases. Otherwise, the reactions would stop in the simulations, since the term of the consumed gas would tend to zero. However, the suggested specific uptake rates to describe the observed kinetic reactions were mathematically formulated to be independent of the growth and linked with yield coefficients. This enables a simple applicable model structure to achieve prediction of the observed processes. The simulated volumetric productivities were in agreement with the experimental data and showed overall the highest volumetric productivities for the *E. cloacae* variants at the first sampling point with 40.86 mg L^−1^ h^−1^ (*budC*) and 38.57 mg L^−1^ h^−1^ (*cag*) compared to *K. pneumoniae* variants with 17.25 mg L^−1^ h^−1^ (*budC*) and 10.13 mg L^−1^ h^−1^ (*cag*). However, the maximum yields from the simulations were found to be almost twice as much for the *E. cloacae* variants, happening before the first sampling point from the experiments, which is in line with the earlier proposed higher volumetric productivities due to the already depleted CO_2_ at the first sampling points. Hence, the mathematical model is simulating a higher resolution of productivities than sampling once a day provides and is potentially showing volumetric productivities which will take place in long-term production experiments without substrate depletion. Additional to selecting the appropriate parameters and defining reasonable constraints, the selection of a suitable objective function and optimization method can significantly impact the estimations of parameters and the resulting simulation outcomes. In the future, this model could be extended to a fed-batch system, to describe control strategies and influences of different nutrient limitations.

## Conclusions

In this study, we developed a carbon- and hydrogen-efficient production strain capable of producing the platform chemical 2,3-butanediol as main product from CO_2_ with a total volumetric productivity of 39.45 mg L^−1^ h^−1^, a total product carbon yield of 81.6%, an H_2_ efficiency of 33.46%, and a specific productivity of 0.016 g of product per gram of biomass per hour under autotrophic conditions. Growth studies have shown that the level of 2,3-BDO in the growth medium, when kept below 500 mM, is somewhat growth inhibiting but not toxic for the strains. In addition, there are several reports on the extraction of butanediol during ongoing fermentations, which offer the possibility of industrially relevant long-term production which may even heighten productivity over time [53]. The applied mathematical model simulations were suitable to describe the batch processes comparable to the experimental data. However, the model structures were individually adapted depending on the observed processes for each strain to achieve an accurate prediction. With this, control strategies with minimum experimentation of the entire process could potentially be developed in the future.

### Supplementary Information

Supplementary material 1.

Supplementary material 2.

Supplementary material 3.

Supplementary material 4.

## Data Availability

The datasets supporting the conclusions of this article are included within the article and its additional files.
