# Supplementary material for: Strain and model development for auto- and heterotrophic 2,3-butanediol production using Cupriavidus necator H16
Source: Biotechnol Biofuels Bioprod. 2024 Jul 30;17:108. doi: 10.1186/s13068-024-02549-7 (PMC11290209; doi:10.1186/s13068-024-02549-7)
Supplement: Supplementary file 1 — Supplementary material 1. [file 13068_2024_2549_MOESM1_ESM.docx]

**Appendix**

Table S1: Used organisms and strains with their relevant genotype and plasmids combined with the used primers and restriction enzymes to produce them.

| ***Organism / Strain***  ***specific strain Number*** | ***Relevant Genotype*** | ***Plasmid*** |  | ***Restriction enzyme*** | ***Primer*** | ***Sequence [5’-3’]*** | ***Source*** |
| --- | --- | --- | --- | --- | --- | --- | --- |
| ***E. coli WM* 3064**  **JG 98** | *thrB1004 pro thi rpsL hsdS lacZ* Δ*M15RP4–1360* Δ(*araBAD*)567 Δ*dapA1341*::[*erm* *pir*(*wt*)] |  |  |  |  |  | W. Metcalf, University of Illinois |
| ***C. necator* H16**  **JG 1070** |  |  |  |  |  |  | DSM 428 |
| ***C. necator* H16 del**  **JG 1232** | ∆*acoABC* ∆*phaC1* ∆*phaC2* |  |  |  |  |  | Windhorst & Gescher 2019 |
| **pKR_*alsSD***  **JG 1233** | ∆*acoABC* ∆*phaC1* ∆*phaC2* | pKR_phb__*alsSD* | *tc^R^*, P_phb_, RSF1010 *mob* and origin of replication, *par*, *alsSD* (codon optimized*) |  |  |  | Windhorst & Gescher 2019 |
| **pKR_*budC* (*K. pneumoniae*)**  **JG 1304** | ∆*acoABC* ∆*phaC1* ∆*phaC2* | pKR_phb__*alsSD* _*budC* | *tc^R^*, P_phb_, RSF1010 *mob* and origin of replication, *par*, *alsSD, budC* (codon optimized*) |  |  |  | *This work* |
|  |  |  |  | *Hind*III, *AP* |  |  | New England Biolabs (NEB; Frankfurt, Germany) |
|  |  |  |  |  | for: 3292 | ATCGAAACCACCGAAGGCT | *This work* |
|  |  |  |  |  | rev: 3293 | TAACTGTGATAAACTACCGCA | *This work* |
|  |  | pBBR1MCS-2 | *km*^R^, P_lac_, *oriV, mob*, *lacZα* |  |  |  | Kovach et al. 1995 |
| **pBBR1_*alsSD***  **JG 1884** | ∆*acoABC* ∆*phaC1* ∆*phaC2* | pBBR1_phb__*alsSD* | *km*^R^, P_phb_, *oriV, mob*, *lacZα, alsSD* (codon optimized*) |  |  |  | *This work* |
|  |  |  |  | *Xba*I, *Bam*HI |  |  | NEB |
|  |  |  |  |  | for: 3464 | aattggagctccaccgcggtggcggccgctGTGTGGGGCCGCACCTAT | *This work* |
|  |  |  |  |  | rev: 3465 | tatcgaattcctgcagcccgggggatccacTTATTCaGGCGAGCCTTCGG | *This work* |
| **pBBR1_*budC* (*K. aerogenes*)**  **JG 1885** | ∆*acoABC* ∆*phaC1* ∆*phaC2* | pBBR1_phb__*alsSD_budC* | *km*^R^, P_phb_, *oriV, mob*, *lacZα, alsSD, budC* (codon optimized*) |  |  |  | *This work* |
|  |  |  |  | *Bam*HI, *Hind*III |  |  | NEB |
|  |  |  |  |  | for: 3292  for: 3514 | ATCGAAACCACCGAAGGCT  gaaaccaccgaaggctcgcctgaataagtgCAGCACTAAATAAAGGAGGT | *This work*  *This work* |
|  |  |  |  |  | rev: 3518 | ccccctcgaggtcgacggtatcgataagctTTAGTTGAACACCATGCCAC | *This work* |
| **pBBR1_*budC* (*K. pneumoniae*)**  **JG 1886** | ∆*acoABC* ∆*phaC1* ∆*phaC2* | pBBR1_phb__*alsSD_budC* | *km*^R^, P_phb_, *oriV, mob*, *lacZα, alsSD, budC* (codon optimized*) |  |  |  | *This work* |
|  |  |  |  | *Bam*HI, *Hind*III |  |  | NEB |
|  |  |  |  |  | for: 3515 | gaaaccaccgaaggctcgcctgaataagtgCAGCACTAAATAAAGGAGGT | *This work* |
|  |  |  |  |  | rev: 3518 | ccccctcgaggtcgacggtatcgataagctTTAGTTGAACACCATGCCAC | *This work* |
| **pBBR1_*budC* (*E. cloacae*)**  **JG 1887** | ∆*acoABC* ∆*phaC1* ∆*phaC2* | pBBR1_phb__*alsSD_budC* | *km*^R^, P_phb_, *oriV, mob*, *lacZα, alsSD, budC* (codon optimized*) |  |  |  | *This work* |
|  |  |  |  | *Bam*HI, *Hind*III |  |  | NEB |
|  |  |  |  |  | for: 3515 | gaaaccaccgaaggctcgcctgaataagtgCAGCACTAAATAAAGGAGGT | *This work* |
|  |  |  |  |  | rev: 3519 | ccccctcgaggtcgacggtatcgataagctTTAGTTGAACACCATCCCAC | *This work* |
| **pBBR1_*cag* (*K. pneumoniae*)**  **JG 1843** | ∆*acoABC* ∆*phaC1* ∆*phaC2* | pBBR1_phb__*alsSD_budC_cag* | *km*^R^, P_phb_, *oriV, mob*, *lacZα, alsSD, budC* (codon optimized*), *cag* |  |  |  | *This work* |
|  |  |  |  | *Hind*III, *Xho*I |  |  | NEB |
|  |  |  |  |  | for: 4077 | ctgatcgacggtggcatggtgttcaactaaACCCTGGCCCTTTAAAGTCC | *This work* |
|  |  |  |  |  | rev: 4112 | gggaacaaaagctgggtaccgggcccccccGGTCGTCAGCCGATCCGC | *This work* |
| **pBBR1_*cag* (*E. cloacae*)**  **JG 1852** | ∆*acoABC* ∆*phaC1* ∆*phaC2* | pBBR1_phb__*alsSD_budC_cag* | *km*^R^, P_phb_, *oriV, mob*, *lacZα, alsSD, budC* (codon optimized*), *cag* |  |  |  | *This work* |
|  |  |  |  | *Hind*III, *Xho*I |  |  | NEB |
|  |  |  |  |  | for: 4113 | ctgattgatggtgggatggtgttcaactaaACCCTGGCCCTTTAAAGTCC | *This work* |
|  |  |  |  |  | rev: 4112 | gggaacaaaagctgggtaccgggcccccccGGTCGTCAGCCGATCCGC | *This work* |

* Sequence is available in the supplements (supplement sequence *alsS,* *alsD,* all *budC* versions and *cag*).


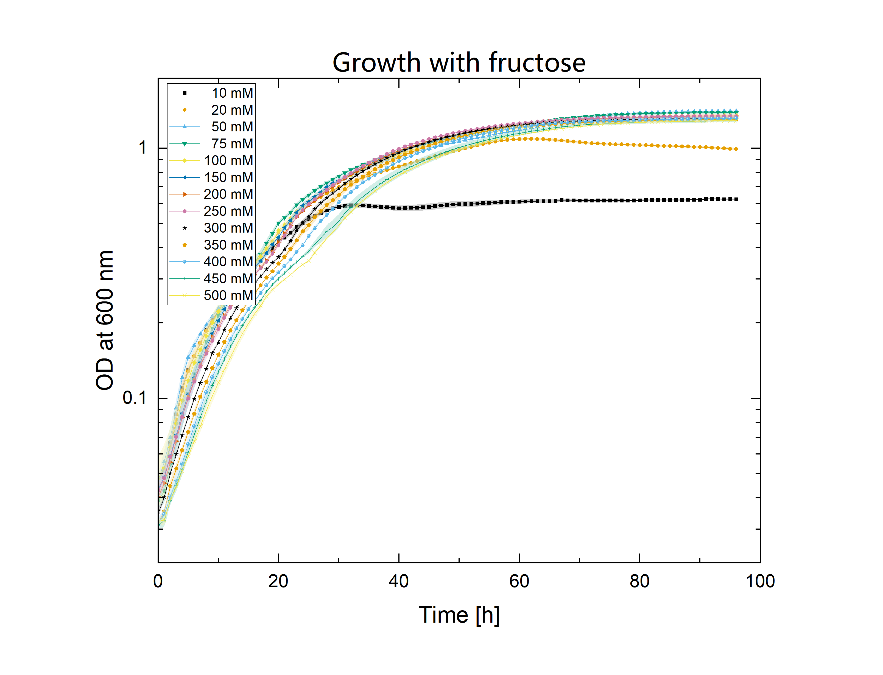

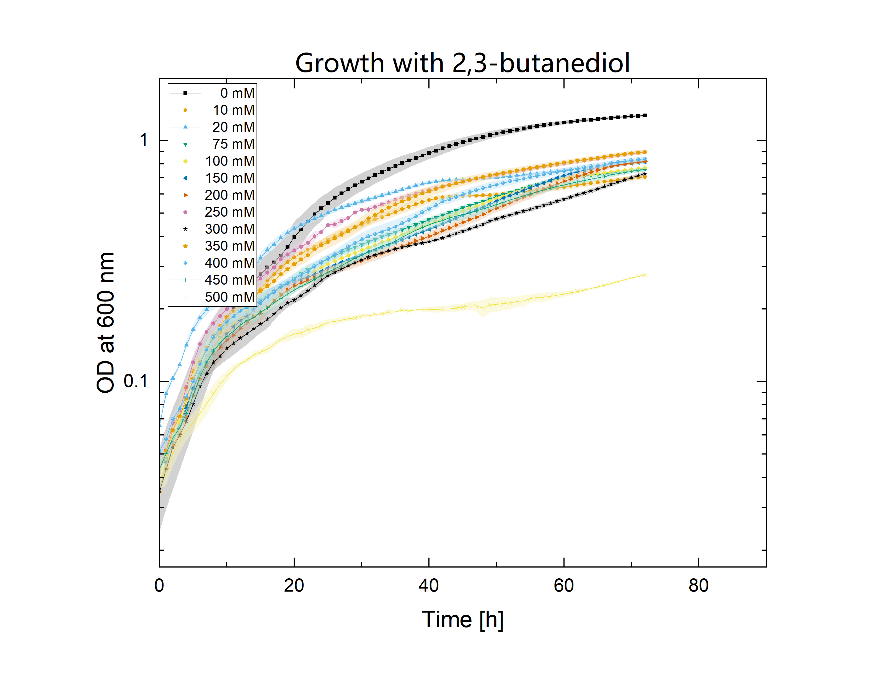


Figure S1: Growth experiments were conducted with C. necator H16 WT using increasing concentrations of fructose (left) and 2,3-BDO (right) over 72 and 98 hours to assess possible substrate and product inhibition. Triplicates are indicated by the surrounding color clouds.


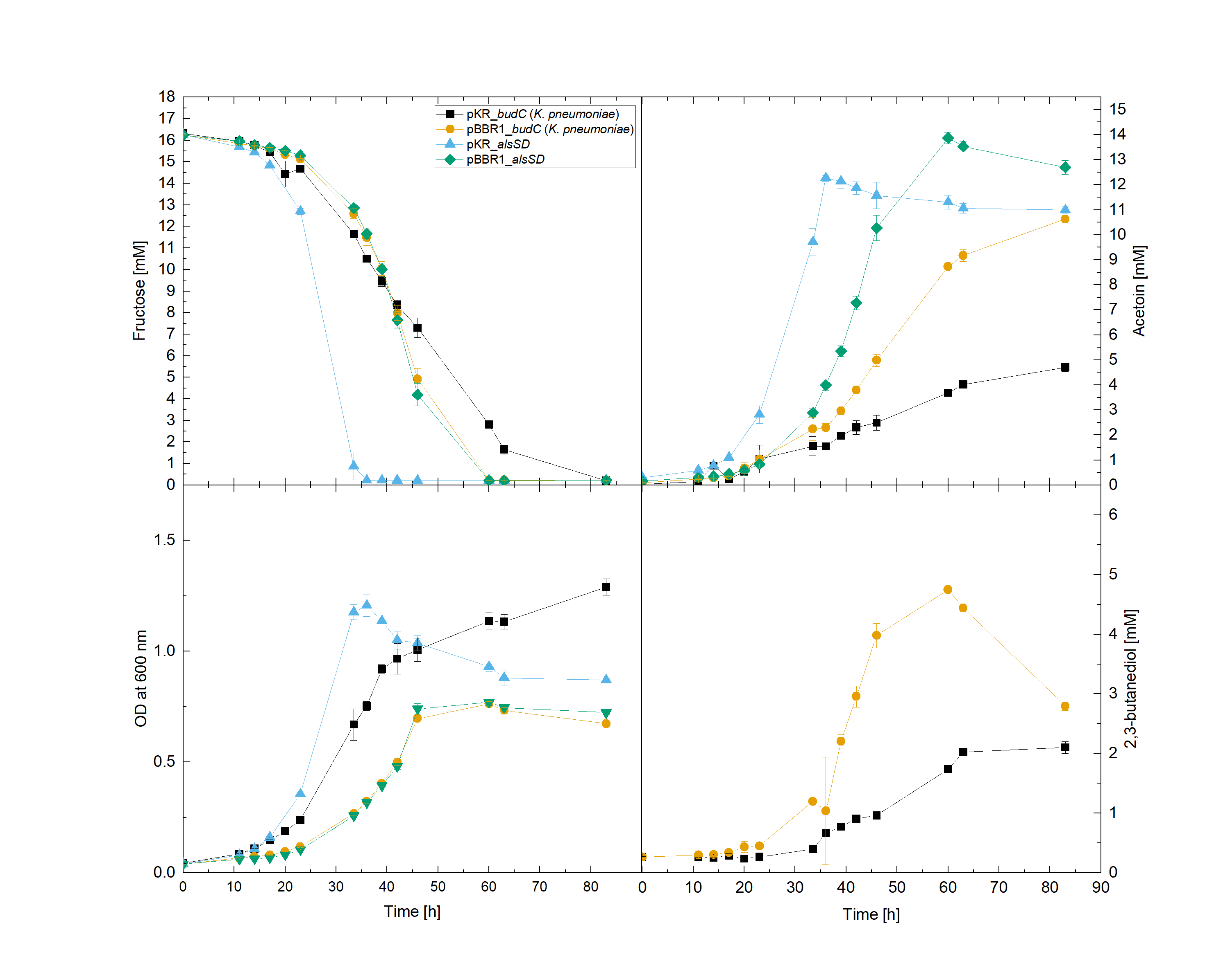


Figure S1: Comparison of production plasmids (organism of origin specified in parentheses) under heterotrophic conditions. For each case, acetoin, 2,3-BDO, fructose and measured OD_600_ values are displayed.


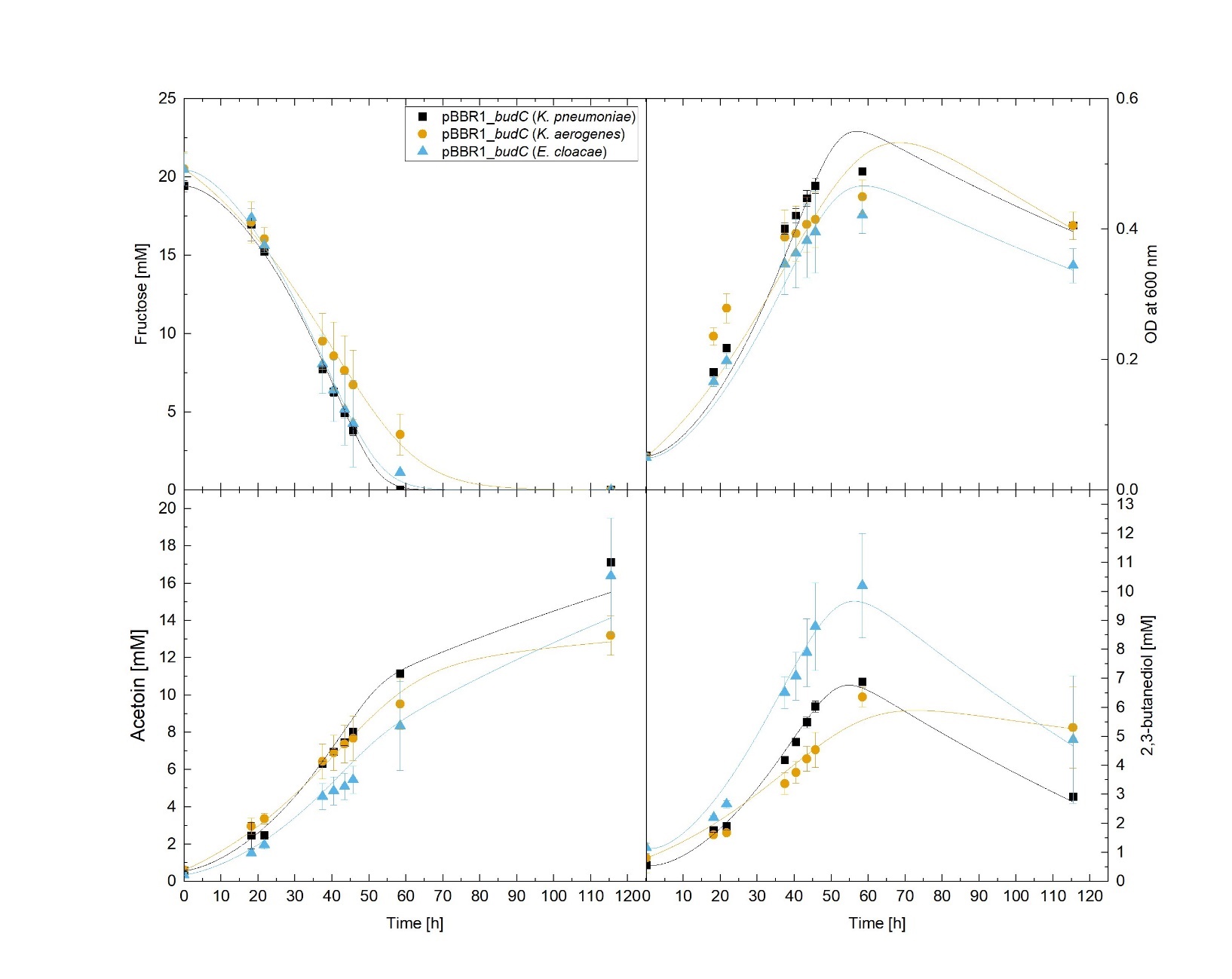


Figure S2: Comparison of variants of budC from different organisms (organism of origin specified in parentheses) under heterotrophic conditions. For each case, acetoin, 2,3-BDO, fructose and measured OD_600_ values are displayed. Measured experiment values are shown in symbols while estimated data from the mathematic model are shown as lines in the corresponding colors.


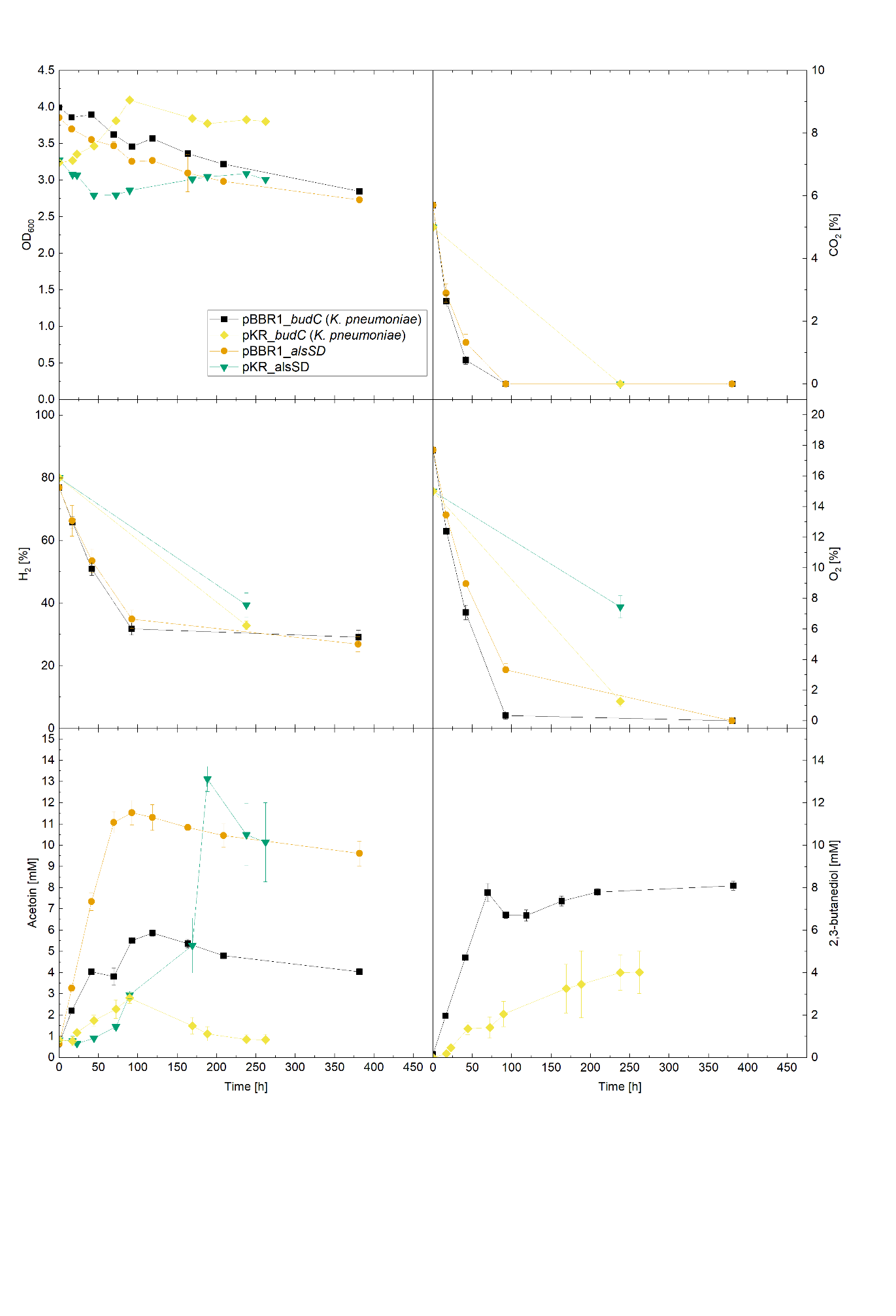


Figure S3: Comparing production plasmids (organism of origin for budC as indicated by organism names in parentheses) under autotrophic conditions. Each case displays acetoin, 2,3-BDO values, optical density and gas measurements.


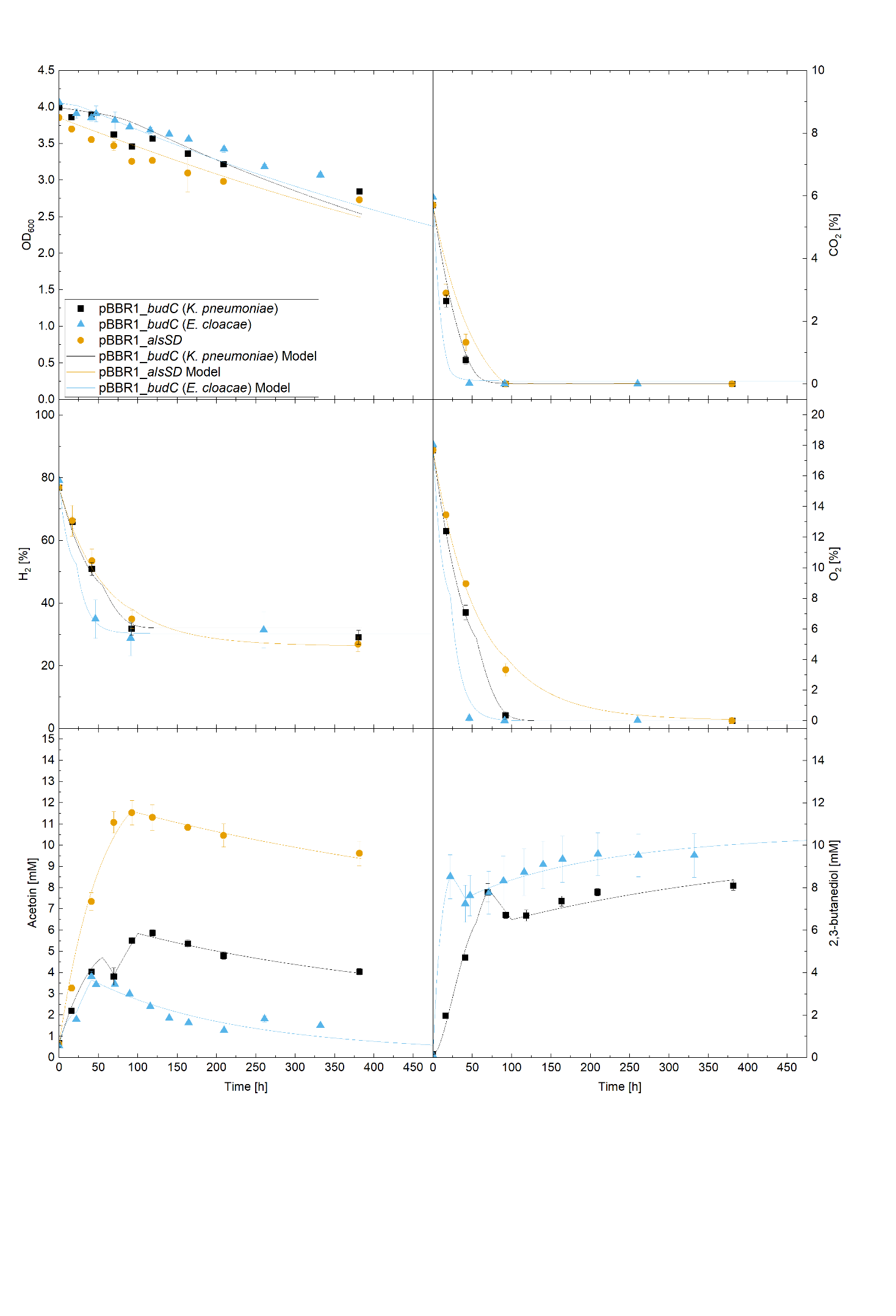


Figure S4: Comparison of variants of budC from different organisms (organism of origin specified in parentheses) under autotrophic conditions. Each case displays acetoin, 2,3-BDO values, optical density and gas measurements. Measured experiment values are shown in symbols while estimated data from the mathematic model are shown as lines in the corresponding colors.


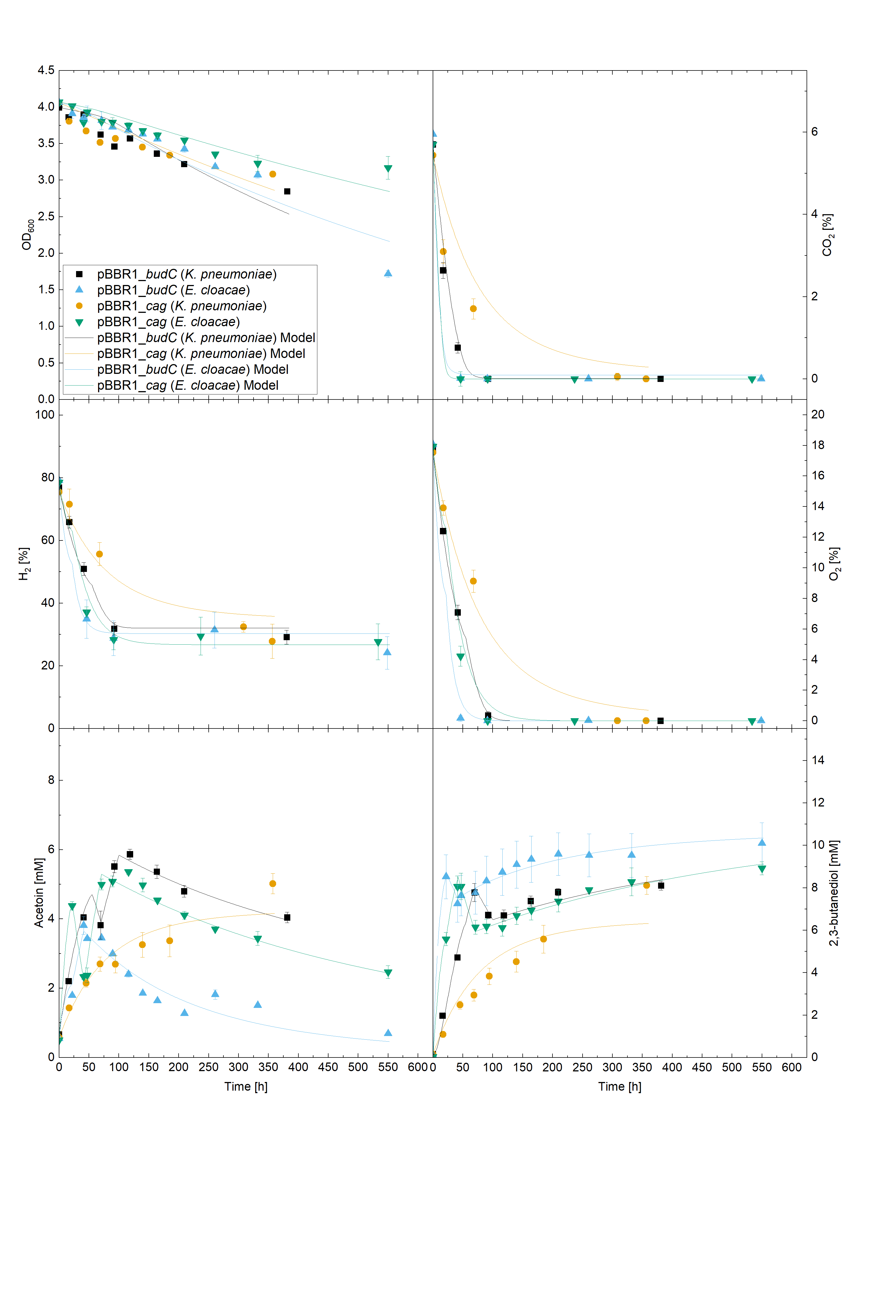


Figure S5: Comparison of budC variations from different organisms (as indicated by organism names in parentheses) and strains carrying a CA gene under autotrophic conditions. Each case displays acetoin, 2,3-BDO values, optical density and gas measurements. Measured experiment values are shown in symbols while estimated data from the mathematic model are shown as lines in the corresponding colors.
