# Supplementary material for: Strain and model development for auto- and heterotrophic 2,3-butanediol production using Cupriavidus necator H16
Source: Biotechnol Biofuels Bioprod. 2024 Jul 30;17:108. doi: 10.1186/s13068-024-02549-7 (PMC11290209; doi:10.1186/s13068-024-02549-7)
Supplement: Supplementary file 2 — Supplementary material 2. [file 13068_2024_2549_MOESM2_ESM.docx]

Codon optimized gene sequences

***budC* *Klebsiella* *pneumoniae***

Locus *budC* 771 bp

Organism *Klebsiella* *pneumoniae* IAM1063

Sequence:

| 1 | ATGAAGAAGG | TGGCCCTGGT | GACCGGTGCA | GGCCAGGGCA | TCGGCAAGGC | CATCGCCCTG |
| --- | --- | --- | --- | --- | --- | --- |
| 61 | CGCCTGGTGA | AGGACGGCTT | CGCCGTGGCC | ATCGCCGACT | ACAACGACGC | CACCGCCAAG |
| 121 | GCCGTGGCCT | CGGAAATCAA | CCAGGCAGGA | GGCCACGCCG | TGGCCGTGAA | GGTGGACGTG |
| 181 | TCGGACCGCG | ACCAGGTGTT | CGCAGCCGTG | GAACAGGCAC | GCAAGACCCT | GGGTGGCTTC |
| 241 | GACGTGATCG | TGAACAACGC | AGGCGTGGCA | CCGTCGACCC | CGATCGAATC | GATCACCCCG |
| 301 | GAAATCGTGG | ACAAGGTGTA | CAACATCAAC | GTGAAGGGCG | TGATCTGGGG | CATCCAGGCA |
| 361 | GCCGTGGAAG | CCTTCAAGAA | GGAAGGCCAC | GGTGGCAAGA | TCATCAACGC | CTGCTCGCAG |
| 421 | GCAGGCCACG | TGGGCAACCC | GGAACTGGCC | GTGTACTCGT | CGTCGAAGTT | CGCCGTGCGT |
| 481 | GGCCTGACCC | AGACCGCAGC | ACGCGACCTG | GCACCGCTGG | GCATCACCGT | GAACGGCTAC |
| 541 | TGCCCTGGCA | TCGTGAAGAC | CCCGATGTGG | GCAGAAATCG | ACCGCCAGGT | GTCGGAAGCC |
| 601 | GCAGGCAAGC | CGCTGGGCTA | CGGCACTGCC | GAATTCGCCA | AGCGCATCAC | CCTGGGTCGC |
| 661 | CTGTCGGAAC | CGGAAGACGT | GGCAGCCTGC | GTGTCGTACC | TGGCCTCGCC | GGACTCGGAC |
| 721 | TACATGACCG | GCCAGTCGCT | GCTGATCGAC | GGTGGCATGG | TGTTCAACTA | A |


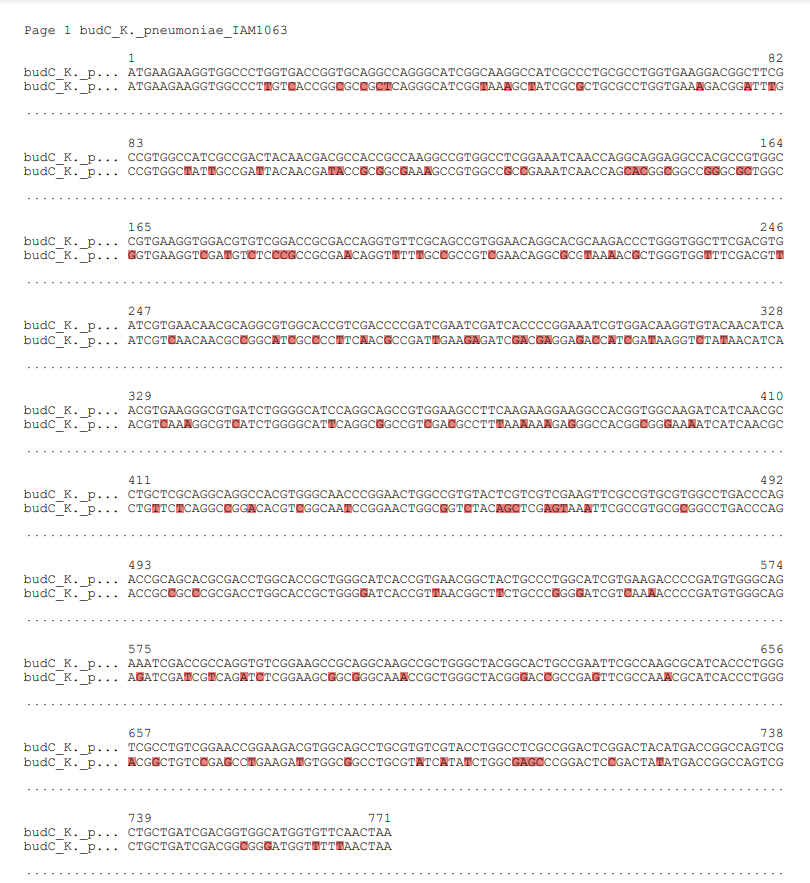


***budC Klebsiella aerogenes***

Locus *budC* 771 bp

Organism *Klebsiella* *aerogenes* AUH-KAM-9

Sequence:

| 1 | ATGAAGAAGG | TGGCCCTGGT | GACCGGTGCA | GGCCAGGGCA | TCGGCAAGGC | CATCGCCCTG |
| --- | --- | --- | --- | --- | --- | --- |
| 61 | CGCCTGGTGA | AGGACGGCTT | CGCCGTGGCC | ATCGCCGACT | ACAACGACGT | GACCGCCAAG |
| 121 | GCCGTGGCCG | ACGAAATCAA | CCAGCACGGA | GGCCGCGCCA | TCGCCGTGAA | GGTGGACGTG |
| 181 | TCGGACCGCG | AGCAGGTGTT | CGCAGCCGTG | GAACAGGCAC | GCAAGACCCT | GGGTGGCTTC |
| 241 | GAGGTGATCG | TGAACAACGC | AGGCGTGGCA | CCGTCGACCC | CGATCGAATC | GATCACCCCG |
| 301 | GAAATCGTGG | ACAAGGTGTA | CAACATCAAC | GTGAAGGGCG | TGATCTGGGG | CATCCAGGCA |
| 361 | GCCGTGGAAG | CCTTCAAGAA | GGAAGGCCAC | GGTGGCAAGA | TCATCAACGC | CTGCTCGCAG |
| 421 | GCAGGCCACG | TGGGCAACCC | GGAACTGGCC | GTGTACTCGT | CGTCGAAGTT | CGCCGTGCGT |
| 481 | GGCCTGACCC | AGACCGCAGC | ACGCGACCTG | GCACCGCTGG | GCATCACCGT | GAACGGCTAC |
| 541 | TGCCCTGGCA | TCGTGAAGAC | CCCGATGTGG | GCAGAAATCG | ACCGCCAGGT | GTCGGAAGCC |
| 601 | GCAGGCAAGC | CGCTGGGCTA | CGGCACTGCC | GAATTCGCCA | AGCGCATCAC | CCTGGGTCGC |
| 661 | CTGTCGGAAC | CGGAAGACGT | GGCAGCCTGC | GTGTCGTACC | TGGCCTCGCC | GGACTCGGAC |
| 721 | TACATGACCG | GCCAGTCGCT | GCTGATCGAC | GGTGGCATGG | TGTTCAACTA | A |


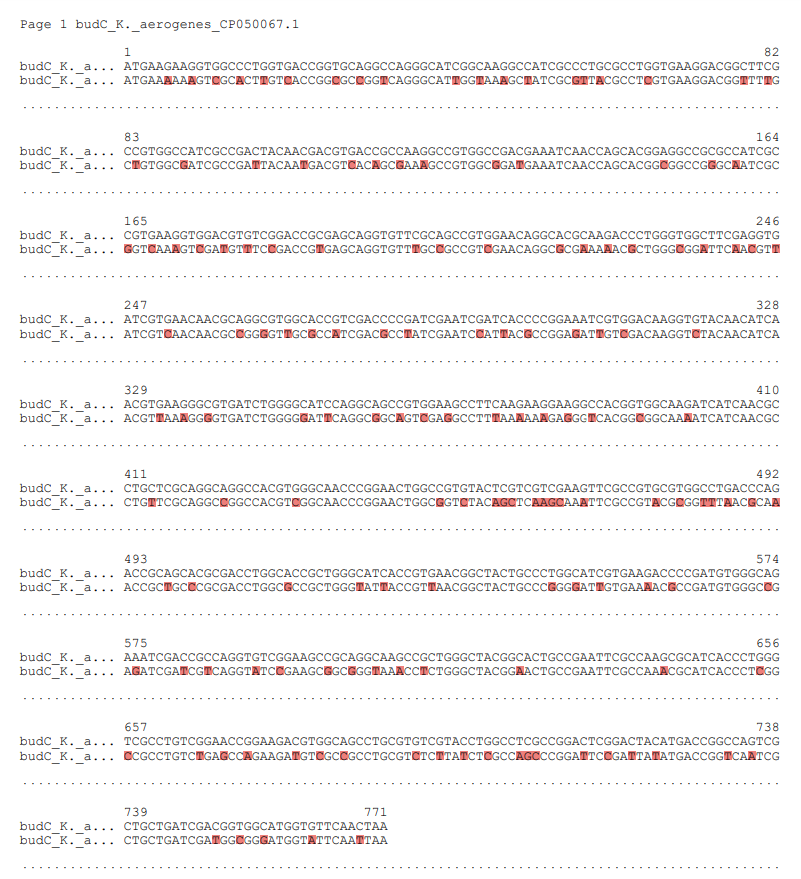


***budC Enterobacter cloacae***

Locus *budC* 771 bp

Organism *Enterobacter* *cloacae* 3849

Sequence:

| 1 | ATGCAAAGAG | TGGCTCTCGT | AACCGGCTCC | GGCCAGGGGA | TTGGCAAAGC | GATCGCGCTT |
| --- | --- | --- | --- | --- | --- | --- |
| 61 | CGCCTGGTGA | AGGACGGCTT | TGCCGTCGCT | ATCGCGGATT | ACAACGAAGA | GACGGCGAAA |
| 121 | GCGGTCGCGG | ATGAGATCAC | CCGCAACGGC | GGGAAAGCCG | TCGCCGTGAA | GGTGGACGTC |
| 181 | TCTGACCGCG | ACCAGGTGTT | TGCGGCGGTG | GAAAAAGCCC | GCACTGCGCT | GGGCGGCTTC |
| 241 | AACGCCATTG | TCAATAACGC | CGGGGTGGCG | CCGTCTACCC | CTATCGAATC | CATCACGCCG |
| 301 | GAAATTGTCG | ACAAGGTCTA | CAACATCAAC | GTGAAAGGGG | TGATCTGGGG | CATTCAGGCC |
| 361 | GCGATTGACG | CGTTCCGCAA | AGAGGGGCAC | GGCGGTAAAA | TCATCAACGC | CTGCTCCCAG |
| 421 | GCGGGCCATA | CCGGCAACCC | GGAACTGGCG | GTGTACAGCT | CCAGCAAGTT | CGCGGTGCGT |
| 481 | GGCTTAACCC | AGACCGCCGC | GCGGGATCTC | GCGCCGCTGG | GGATCACCGT | TAACGCCTAT |
| 541 | TGCCCGGGCA | TCGTCAAAAC | GCCGATGTGG | GCGGAAATCG | ACCGTCAGGT | CTCCGAGGCG |
| 601 | GCGGGTAAAC | CGCTCGGCTA | CGGAACGGAA | ACCTTTGCCA | AACGCATCAC | GCTTGGCCGT |
| 661 | TTGTCCGAAC | CGGAAGATGT | GGCCGCCTGC | GTCTCTTACC | TCGCCGGACC | AGACTCCGAC |
| 721 | TACATGACCG | GTCAGTCGCT | GCTGATTGAT | GGTGGGATGG | TGTTCAACTA | A |

***
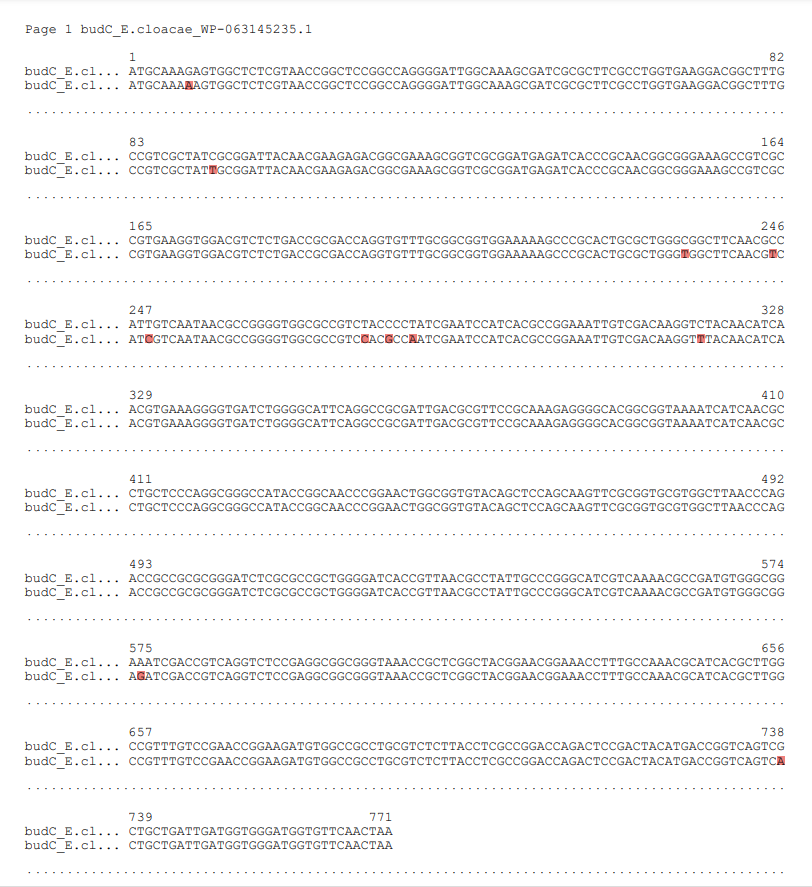
***

***Cag (naturally occurring)***

Locus *cag* 525 bp

Organism *Cupriavidus* *necator* H16

Sequence:

| 1 | ATGGCGCTTT | ACCAGCTCGG | CGACGTCAAG | CCCAATATCG | ACAGCGATGC | CTACGTGGCC |
| --- | --- | --- | --- | --- | --- | --- |
| 61 | CCCGAAGCCA | CCGTCATCGG | CAACGTGACC | CTCAAGTCCC | GCGCCAGCGC | CTGGCCCGGT |
| 121 | GTCGTGATCC | GCGGCGACAA | CGAACCGATC | GTGGTGGGCG | AGGACACCAA | CATCCAGGAA |
| 181 | GGCTCGGTGC | TGCATACCGA | CCCGGGCTGC | CCGCTGACCC | TGGGCGACAA | GGTCTCGATC |
| 241 | GGCCACCAGG | CCATGCTGCA | TGGCTGCACC | GTAGGCGAAG | GCTCGCTCAT | CGGGATCCAG |
| 301 | GCCGTGGTGC | TGAACCGCGC | CGTGATCGGC | AAGGAATGCC | TGGTCGGCGC | CGGTGCCGTG |
| 361 | GTGACCGAGG | GCAAGGTCTT | CCCGGACCGC | TCGCTGATCC | TGGGCGCGCC | GGCCAAGGTG |
| 421 | GTGCGCCAGC | TGACCGACGC | CGACGTCGCC | AACCTGTACC | GCAACGCCGA | GACCTACGCC |
| 481 | ACCCGGCAGG | CCATGTACAA | GCAACAGCTC | AAGCGGATCG | GCTGA |  |
