# Supplementary material for: Strain and model development for auto- and heterotrophic 2,3-butanediol production using Cupriavidus necator H16
Source: Biotechnol Biofuels Bioprod. 2024 Jul 30;17:108. doi: 10.1186/s13068-024-02549-7 (PMC11290209; doi:10.1186/s13068-024-02549-7)
Supplement: Supplementary file 3 — Supplementary material 3. [file 13068_2024_2549_MOESM3_ESM.docx]

Table S2: Estimates of the coefficient of determination in heterotrophic batch process for each strain

|  | Strain | | |
| --- | --- | --- | --- |
| Model efficiency R^2^ | pBBR1_budC  (*K. pneumoniae)* | pBBR1_budC  (*K. aerogenes)* | pBBR1_budC  (*E. cloacae)* |
| Optical density | 0.94 | 0.85 | 0.95 |
| Fructose | 0.99 | 0.99 | 0.99 |
| Acetoin | 0.98 | 0.99 | 0.96 |
| 2,3-BDO | 0.99 | 0.94 | 0.98 |

Table S3: Estimates of the coefficient of determination in autotrophic batch process for each strain

|  | Strain | | | | |
| --- | --- | --- | --- | --- | --- |
| Model efficiency R^2^ | pBBR1_budC  (*E. cloacae)* | pBBR1_cag  (*E. cloacae)* | pBBR1_budC  (*K. pneumoniae)* | pBBR1_cag  (*K. pneumoniae)* | pBBR1_  alsSD |
| Optical density | 0.91 | 0.76 | 0.73 | 0.57 | 0.83 |
| Acetoin | 0.90 | 0.97 | 0.99 | 0.90 | 0.98 |
| 2,3-BDO | 0.99 | 0.99 | 0.99 | 0.88 | 0.96 |
| CO_2,g_ | 0.99 | 0.99 | 0.98 | 0.88 | 0.95 |
| O_2,g_ | 0.99 | 0.99 | 0.99 | 0.98 | 0.99 |
| H_2,g_ | 0.98 | 0.98 | 0.98 | 0.96 | 0.98 |

Table S4: Parameter estimates in heterotrophic batch process

|  | Strain | | |
| --- | --- | --- | --- |
|  | pBBR1_budC  (*K. pneumoniae)* | pBBR1_budC  (*K. aerogenes)* | pBBR1_budC  (*E. cloacae)* |
| $\mu_{max}$ [h^-1^] | 4.40$\cdot$10^-2^ | 5.00$\cdot$10^-2^ | 4.61$\cdot$10^-2^ |
| $\mu_{d,min}$ [h^-1^] | 6.26$\cdot$10^-6^ | 1.59$\cdot$10^-6^ | 1.22$\cdot$10^-6^ |
| $\mu_{d,max}$ [h^-1^] | 4.90$\cdot$10^-3^ | 6.39$\cdot$10^-3^ | 5.02$\cdot$10^-3^ |
| $Y_{X,F}$ [g_cell_ g^-1^] | 10.0$\cdot$10^-2^ | 10.7$\cdot$10^-2^ | 8.3$\cdot$10^-2^ |
| $Y_{X,A}$ [g_cell_ g^-1^] | 0.49 | 0.43 | 0.76 |
| $Y_{X,B}$ [g_cell_ g^-1^] | 0.43 | 0.71 | 0.27 |
| $Y_{A,B}$ [g g^-1^] | 1.03 | 0.98 | 1.04 |
| $K_{S,F}$ [g L^-1^] | 0.55 | 1.68 | 0.90 |
| $K_{P,B}$ [g L^-1^] | 1.03$\cdot$10^-3^ | 1.12 | 1.03$\cdot$10^-3^ |
| $q_{B,max}$ [g g_cell_^-1^ h^-1^] | 1.68$\cdot$10^-2^ | 1.63$\cdot$10^-2^ | 2.49$\cdot$10^-2^ |
| $t_{lag}$ [h] | 6.00 | 2.43$\cdot$10^-5^ | 6.00 |
| $S_{F,min}$ [g L^-1^] | 5.00$\cdot$10^-2^ | 5.55$\cdot$10^-2^ | 5.02$\cdot$10^-2^ |

Table S5: Parameter estimates in autotrophic batch process for each strain

| Strain | | | | | |
| --- | --- | --- | --- | --- | --- |
|  | pBBR1_budC  (*E. cloacae)* | pBBR1_cag  (*E. cloacae)* | pBBR1_budC  (*K. pneumoniae)* | pBBR1_cag  (*K. pneumoniae)* | pBBR1_  alsSD |
| $\mu_{max}$ [h^-1^] | 6.13$\cdot$10^-2^ | 1.18$\cdot$10^-3^ | 1.15$\cdot$10^-3^ | 7.48$\cdot$10^-3^ | 9.61$\cdot$10^-3^ |
| $\mu_{d,min}$ [h^-1^] | 2.69$\cdot$10^-4^ | 5.48$\cdot$10^-4^ | 3.48$\cdot$10^-4^ | 9.72$\cdot$10^-4^ | 9.80$\cdot$10^-4^ |
| $\mu_{d,max}$ [h^-1^] | 7.83$\cdot$10^-3^ | 1.78$\cdot$10^-3^ | 1.00$\cdot$10^-3^ | 7.96$\cdot$10^-2^ | 1.94$\cdot$10^-3^ |
| $k_{L}a$ [h^-1^] | 159 | 142 | 155 | 156 | 163 |
| $Y_{X,A}$ [g_cell_ g^-1^] | 2.32$\cdot$10^-2^ | 3.07$\cdot$10^-2^ | 0.62$\cdot$10^-2^ | 8.54$\cdot$10^-2^ | 0.12$\cdot$10^-3^ |
| $Y_{X,B}$ [g_cell_ g^-1^] | 0.45$\cdot$10^-2^ | 2.14$\cdot$10^-2^ | 0.22$\cdot$10^-2^ | 4.86$\cdot$10^-2^ | 0.44$\cdot$10^-3^ |
| $Y_{X,{CO}_{2}}$ [g_cell_ mol^-1^] | 7.37$\cdot$10^-2^ | 24.7$\cdot$10^-2^ | 5.22$\cdot$10^-2^ | 66.0$\cdot$10^-2^ | 0.27$\cdot$10^-2^ |
| $Y_{X,O_{2}}$ [g_cell_ mol^-1^] | 4.29$\cdot$10^-2^ | 26.0$\cdot$10^-2^ | 2.37$\cdot$10^-2^ | 20.9$\cdot$10^-2^ | 0.11$\cdot$10^-2^ |
| $Y_{X,H_{2}}$ [g_cell_ mol^-1^] | 1.59$\cdot$10^-2^ | 9.02$\cdot$10^-2^ | 0.93$\cdot$10^-2^ | 8.90$\cdot$10^-2^ | 0.41$\cdot$10^-3^ |
| $Y_{r,A,B}$ [g g^-1^] | 0.94 | 1.17 | 1.18 | - | - |
| $Y_{f1,B,A}$ [g g^-1^] | - | 1.13 | 1.15 | - | - |
| $Y_{f2,B,A}$ [g g^-1^] | 0.89 | 1.13 | 1.02 | - | 1.14 |
| $K_{{CO}_{2}}$ [mol L^-1^] | 8.58$\cdot$10^-4^ | 4.15$\cdot$10^-4^ | 1.93$\cdot$10^-4^ | 8.36$\cdot$10^-2^ | 1.27$\cdot$10^-5^ |
| $K_{O_{2}}$ [mol L^-1^] | 4.92$\cdot$10^-2^ | 3.85$\cdot$10^-4^ | 1.21$\cdot$10^-5^ | 1.01$\cdot$10^-5^ | 1.61 |
| $K_{H_{2}}$ [mol L^-1^] | 2.88$\cdot$10^-5^ | 1.39$\cdot$10^-5^ | 1.97$\cdot$10^-2^ | 1.25$\cdot$10^-5^ | 1.63$\cdot$10^-5^ |
| $K_{f1,A}$ [g L^-1^] | - | 1.03 | 0.05 | - | - |
| $K_{f2,A}$ [g L^-1^] | 5.68 | 5.54 | 6.24 | - | 6.38 |
| $K_{r,B}$ [g L^-1^] | 0.73 | 0.65 | 0.59 | - | - |
| $q_{f1,A,max}$  [g g_cell_^-1^ h^-1^] | - | 1.85$\cdot$10^-2^ | 0.25$\cdot$10^-2^ | - | - |
| $q_{f2,A,max}$  [g g_cell_^-1^ h^-1^] | 1.59$\cdot$10^-2^ | 0.42$\cdot$10^-2^ | 0.44$\cdot$10^-2^ | - | 0.27$\cdot$10^-2^ |
| $q_{r,B,max}$  [g g_cell_^-1^ h^-1^] | 0.54$\cdot$10^-2^ | 0.61$\cdot$10^-2^ | 0.35$\cdot$10^-2^ | - | - |
