# Supplementary material for: Strain and model development for auto- and heterotrophic 2,3-butanediol production using Cupriavidus necator H16
Source: Biotechnol Biofuels Bioprod. 2024 Jul 30;17:108. doi: 10.1186/s13068-024-02549-7 (PMC11290209; doi:10.1186/s13068-024-02549-7)
Supplement: Supplementary file 4 — Supplementary material 4. [file 13068_2024_2549_MOESM4_ESM.docx]

**Heterotroph Script**

clc
clear all
close all

%molare Massen
Acetoin = 88.11; %g/mol
zweidreiButandiol = 90.121; %g/mol
Fructose = 180.16; %g/mol

% Data imported from Excel
Data_K_pneumoniae_OD=xlsread('HeterotrophResults.xlsx','K.pneumoniae','I2:I10'); %OD600
Data_K_pneumoniae_Time=xlsread('HeterotrophResults.xlsx','K.pneumoniae','E2:E10'); %h
Data_K_pneumoniae_Fructose=xlsread('HeterotrophResults.xlsx','K.pneumoniae','O2:O10'); %mM
Data_K_pneumoniae_Acetoin=xlsread('HeterotrophResults.xlsx','K.pneumoniae','Y2:Y10'); %mM
Data_K_pneumoniae_zweidreiButandiol=xlsread('HeterotrophResults.xlsx','K.pneumoniae','AN2:AN10'); %mM
Data_K_pneumoniae_BTM=0.6416.*Data_K_pneumoniae_OD+0.0632; %BTM g/L

Data_K_aerogenes_OD=xlsread('HeterotrophResults.xlsx','K.aerogenes','I2:I10'); %OD600
Data_K_aerogenes_Time=xlsread('HeterotrophResults.xlsx','K.aerogenes','E2:E10'); %h
Data_K_aerogenes_Fructose=xlsread('HeterotrophResults.xlsx','K.aerogenes','O2:O10'); %mM
Data_K_aerogenes_Acetoin=xlsread('HeterotrophResults.xlsx','K.aerogenes','Y2:Y10'); %mM
Data_K_aerogenes_zweidreiButandiol=xlsread('HeterotrophResults.xlsx','K.aerogenes','AN2:AN10'); %mM
Data_K_aerogenes_BTM=0.6416.*Data_K_aerogenes_OD+0.0632; %BTM g/L

Data_E_cloacae_OD=xlsread('HeterotrophResults.xlsx','E.cloacae','I2:I10'); %OD600
Data_E_cloacae_Time=xlsread('HeterotrophResults.xlsx','E.cloacae','E2:E10'); %h
Data_E_cloacae_Fructose=xlsread('HeterotrophResults.xlsx','E.cloacae','O2:O10'); %mM
Data_E_cloacae_Acetoin=xlsread('HeterotrophResults.xlsx','E.cloacae','Y2:Y10'); %mM
Data_E_cloacae_zweidreiButandiol=xlsread('HeterotrophResults.xlsx','E.cloacae','AN2:AN10'); %mM
Data_E_cloacae_BTM=0.6416.*Data_E_cloacae_OD+0.0632; %BTM g/L

Data_K_pneumoniae_Fructose_SI = (Data_K_pneumoniae_Fructose./1000).*Fructose; %g/L
Data_K_pneumoniae_Acetoin_SI = (Data_K_pneumoniae_Acetoin./1000).*Acetoin; %g/L
Data_K_pneumoniae_zweidreiButandiol_SI = (Data_K_pneumoniae_zweidreiButandiol./1000).*zweidreiButandiol; %g/L

Data_K_aerogenes_Fructose_SI = (Data_K_aerogenes_Fructose./1000).*Fructose; %g/L
Data_K_aerogenes_Acetoin_SI = (Data_K_aerogenes_Acetoin./1000).*Acetoin; %g/L
Data_K_aerogenes_zweidreiButandiol_SI = (Data_K_aerogenes_zweidreiButandiol./1000).*zweidreiButandiol; %g/L

Data_E_cloacae_Fructose_SI = (Data_E_cloacae_Fructose./1000).*Fructose; %g/L
Data_E_cloacae_Acetoin_SI = (Data_E_cloacae_Acetoin./1000).*Acetoin; %g/L
Data_E_cloacae_zweidreiButandiol_SI = (Data_E_cloacae_zweidreiButandiol./1000).*zweidreiButandiol; %g/L

% Defines intitial values from data
Kp0 = [Data_K_pneumoniae_BTM(1,1), Data_K_pneumoniae_Fructose_SI(1,1), Data_K_pneumoniae_Acetoin_SI(1,1), Data_K_pneumoniae_zweidreiButandiol_SI(1,1)];

Ka0 = [Data_K_aerogenes_BTM(1,1), Data_K_aerogenes_Fructose_SI(1,1), Data_K_aerogenes_Acetoin_SI(1,1), Data_K_aerogenes_zweidreiButandiol_SI(1,1)];

Ec0 = [Data_E_cloacae_BTM(1,1), Data_E_cloacae_Fructose_SI(1,1), Data_E_cloacae_Acetoin_SI(1,1), Data_E_cloacae_zweidreiButandiol_SI(1,1)];


% starting values for curve fitting (choose by hand)
mumax = 0.008;
Y_xS = 0.08;
Y_PxA = 0.3;
Y_PxB = 0.2;
Y_PAB = 1.1;
Ks = 0.5;
Kd = 0.00001;
mudmin = 0.0005;
mudmax = 0.001;
qBmax = 0.02;
KSB = 0.8;
Smin = 0.003;
t_lag = 2;
KIB = 0.005;
parnum = 13;

## Use fminocon

for i = 1:3

% transfer of parameters to vector
par0 = [mumax, Y_xS,Y_PxA, Y_PxB, Y_PAB, Ks,mudmin,mudmax,qBmax, KSB,Smin,t_lag,KIB];

% Lower bounds
lowerBounds = [0, 0.001, 0.001, 0.1, 0.9, 0.001, 0, 0, 0, 0.001, 0, 0, 0.0001];
% Upper bounds
upperBounds = [0.05, 1, 1, 2, 1.2, 2, 0.001, 0.008, 4, 2, 0.1, 6, 4];

% structure/options for curve fitting (optional)
options = optimset('Display','Iter','MaxIter',1000,'TolFun',1e-8,'TolX',1e-8);

if i == 1
inputdata = Kp0;
elseif i == 2
inputdata = Ka0;
elseif i == 3
inputdata = Ec0;
end

paropt = fmincon(@objective, par0, [], [], [], [], lowerBounds, upperBounds, [], options,i,inputdata,Data_K_pneumoniae_BTM,Data_K_pneumoniae_Time,Data_K_pneumoniae_Fructose_SI,Data_K_pneumoniae_Acetoin_SI,Data_K_pneumoniae_zweidreiButandiol_SI,...
 Data_K_aerogenes_BTM,Data_K_aerogenes_Time,Data_K_aerogenes_Fructose_SI,Data_K_aerogenes_Acetoin_SI,Data_K_aerogenes_zweidreiButandiol_SI,...
 Data_E_cloacae_BTM,Data_E_cloacae_Time,Data_E_cloacae_Fructose_SI,Data_E_cloacae_Acetoin_SI,Data_E_cloacae_zweidreiButandiol_SI);

if i == 1
 Results_Kp0(i,:) = [i paropt]; % safe estimated parameters
elseif i == 2
 Results_Ka0(i,:) = [i paropt];
elseif i == 3
 Results_Ec0(i,:) = [i paropt];
end

end

## Final Simulation

for i = 1:3
if i == 1
% Final simulation
options=odeset('reltol',1E-12,'abstol',1E-12,'maxstep',0.1,'normcontrol','on');
tspan = Data_K_pneumoniae_Time(:,1);
ttspan = 0:0.1:max(tspan);

[t,c] = ode15s(@DGL,ttspan,Kp0,' ', Results_Kp0(i,2:end));

figure('Name','K. pneumoniae','NumberTitle','off');
subplot(2,1,1)
yyaxis left
plot(ttspan,(c(:,1)-0.0632)./0.6416,'-k',tspan,Data_K_pneumoniae_OD(:,1),'ok');
x1=xlabel('Time [h]');
y1=ylabel('OD_{600}');
legend('OD_{600}','exp. Data OD_{600}','Fructose', 'exp. Data Fructose','Location','southeast','NumColumns',2);
x1.Color='black';
y1.Color='black';
ax = gca;
ax.YColor = 'k';
yyaxis right
plot(ttspan,c(:,2),'-r',tspan,Data_K_pneumoniae_Fructose_SI(:,1),'or');
legend('OD_{600}','exp. Data OD_{600}','Fructose', 'exp. Data Fructose','Location','southeast','NumColumns',2);
y2=ylabel('Fructose [g L^-^1]');
y2.Color='black';
ax = gca;
ax.YColor = 'k';
hold off

subplot(2,1,2)
yyaxis left
plot(ttspan,c(:,3),'-b',tspan,Data_K_pneumoniae_Acetoin_SI(:,1),'ob');
x1=xlabel('Time [h]');
y1=ylabel('Acetoin [g L^-^1]');
legend('Acetoin','exp. Data Acetoin','Butandiol','exp. Data Butandiol','Location','southeast','NumColumns',2);
x1.Color='black';
y1.Color='black';
ax = gca;
ax.YColor = 'k';
hold on
yyaxis right
plot(ttspan,c(:,4),'-g',tspan,Data_K_pneumoniae_zweidreiButandiol_SI(:,1),'og');
legend('Acetoin','exp. Data Acetoin','Butandiol','exp. Data Butandiol','Location','southeast','NumColumns',2);
y2=ylabel('2,3-Butandiol[g L^-^1]');
y2.Color='black';
ax = gca;
ax.YColor = 'k';
hold off

out_file = 'Butandiolfitspaper';
xlswrite(out_file,(c(:,1)-0.0632)./0.6416,'Fit Heterotroph','B4'); %OD
xlswrite(out_file,c(:,2)./Fructose.*1000,'Fit Heterotroph','C4'); % Fructose mM
xlswrite(out_file,c(:,3)./Acetoin.*1000,'Fit Heterotroph','D4'); % Acetoin mM
xlswrite(out_file,c(:,4)./zweidreiButandiol.*1000,'Fit Heterotroph','E4'); % Butandiol mM
xlswrite(out_file,ttspan.','Fit Heterotroph','A4'); %time
xlswrite(out_file,Results_Kp0(1,2:end),'Fit Heterotroph','Q8'); %paropt

% options=odeset('reltol',1E-12,'abstol',1E-12,'maxstep',0.1,'normcontrol','on');
tspan = Data_K_pneumoniae_Time(:,1);
[t,c] = ode15s(@DGL,tspan,Kp0,'',Results_Kp0(i,2:end));

Kp_ODE_R2 = [ c(:,1), c(:,2), c(:,3), c(:,4)];

% R2
R2_Kp_BTM = 1-(sum((Data_K_pneumoniae_BTM(:,1)-c(:,1)).^2)./(sum((Data_K_pneumoniae_BTM(:,1)-mean(Data_K_pneumoniae_BTM(:,1))).^2)));
R2_Kp_S = 1-(sum((Data_K_pneumoniae_Fructose_SI(:,1)-c(:,2)).^2)./(sum((Data_K_pneumoniae_Fructose_SI(:,1)-mean(Data_K_pneumoniae_Fructose_SI(:,1))).^2)));
R2_Kp_Ac = 1-(sum((Data_K_pneumoniae_Acetoin_SI(:,1)-c(:,3)).^2)./(sum((Data_K_pneumoniae_Acetoin_SI(:,1)-mean(Data_K_pneumoniae_Acetoin_SI(:,1))).^2)));
R2_Kp_Bu = 1-(sum((Data_K_pneumoniae_zweidreiButandiol_SI(:,1)-c(:,4)).^2)./(sum((Data_K_pneumoniae_zweidreiButandiol_SI(:,1)-mean(Data_K_pneumoniae_zweidreiButandiol_SI(:,1))).^2)));

R2_Kp_overview = [R2_Kp_BTM R2_Kp_S R2_Kp_Ac R2_Kp_Bu];

xlswrite(out_file,R2_Kp_overview(:,:),'Fit Heterotroph','Q4');

elseif i ==2

tspan2 = Data_K_aerogenes_Time(:,1);
ttspan2 = 0:0.1:max(tspan2);

[t,c] = ode15s(@DGL,ttspan2,Ka0,' ',Results_Ka0(i,2:end));

figure('Name','K. aerogenes','NumberTitle','off');
% hold all
subplot(2,1,1)
yyaxis left
plot(ttspan2,(c(:,1)-0.0632)./0.6416,'-k',tspan2,Data_K_aerogenes_OD(:,1),'ok');
x1=xlabel('Time [h]');
y1=ylabel('OD_{600}');
x1.Color='black';
y1.Color='black';
ax = gca;
ax.YColor = 'k';
hold on
yyaxis right
plot(ttspan2,c(:,2),'-r',tspan2,Data_K_aerogenes_Fructose_SI(:,1),'or');
y2=ylabel('Fructose [g L^-^1]');
y2.Color='black';
ax = gca;
ax.YColor = 'k';
hold off

subplot(2,1,2)
yyaxis left
plot(ttspan2,c(:,3),'-b',tspan2,Data_K_aerogenes_Acetoin_SI(:,1),'ob');
x1=xlabel('Time [h]');
y1=ylabel('Acetoin [g L^-^1]');
x1.Color='black';
y1.Color='black';
ax = gca;
ax.YColor = 'k';
hold on
yyaxis right
plot(ttspan2,c(:,4),'-g',tspan2,Data_K_aerogenes_zweidreiButandiol_SI(:,1),'og');
y2=ylabel('2,3-Butandiol[g L^-^1]');
y2.Color='black';
ax = gca;
ax.YColor = 'k';
hold off

xlswrite(out_file,(c(:,1)-0.0632)./0.6416,'Fit Heterotroph','G4'); %OD
xlswrite(out_file,c(:,2)./Fructose.*1000,'Fit Heterotroph','H4'); % Fructose mM
xlswrite(out_file,c(:,3)./Acetoin.*1000,'Fit Heterotroph','I4'); % Acetoin mM
xlswrite(out_file,c(:,4)./zweidreiButandiol.*1000,'Fit Heterotroph','J4'); % Butandiol mM
xlswrite(out_file,Results_Ka0(2,2:end),'Fit Heterotroph','Q9'); %paropt

tspan2 = Data_K_aerogenes_Time(:,1);

[t,c] = ode15s(@DGL,tspan2,Ka0,' ',Results_Ka0(i,2:end));

Ka_ODE_R2 = [ c(:,1), c(:,2), c(:,3), c(:,4)];

R2_Ka_BTM = 1-(sum((Data_K_aerogenes_BTM(:,1)-c(:,1)).^2)./(sum((Data_K_aerogenes_BTM(:,1)-mean(Data_K_aerogenes_BTM(:,1))).^2)));
R2_Ka_S = 1-(sum((Data_K_aerogenes_Fructose_SI(:,1)-c(:,2)).^2)./(sum((Data_K_aerogenes_Fructose_SI(:,1)-mean(Data_K_aerogenes_Fructose_SI(:,1))).^2)));
R2_Ka_Ac = 1-(sum((Data_K_aerogenes_Acetoin_SI(:,1)-c(:,3)).^2)./(sum((Data_K_aerogenes_Acetoin_SI(:,1)-mean(Data_K_aerogenes_Acetoin_SI(:,1))).^2)));
R2_Ka_Bu = 1-(sum((Data_K_aerogenes_zweidreiButandiol_SI(:,1)-c(:,4)).^2)./(sum((Data_K_aerogenes_zweidreiButandiol_SI(:,1)-mean(Data_K_aerogenes_zweidreiButandiol_SI(:,1))).^2)));

R2_Ka_overview = [R2_Ka_BTM R2_Ka_S R2_Ka_Ac R2_Ka_Bu];

xlswrite(out_file,R2_Ka_overview(:,:),'Fit Heterotroph','Q5');

elseif i == 3

tspan3 = Data_E_cloacae_Time(:,1);
ttspan3 = 0:0.1:max(tspan3);

[t,c] = ode15s(@DGL,ttspan3,Ec0,' ',Results_Ec0(i,2:end));

figure('Name','E. cloacae','NumberTitle','off');
subplot(2,1,1)
yyaxis left
plot(ttspan3,(c(:,1)-0.0632)./0.6416,'-k',tspan3,Data_E_cloacae_OD(:,1),'ok');
x1=xlabel('Time [h]');
y1=ylabel('OD_{600}');
x1.Color='black';
y1.Color='black';
ax = gca;
ax.YColor = 'k';
hold on
yyaxis right
plot(ttspan3,c(:,2),'-r',tspan3,Data_E_cloacae_Fructose_SI(:,1),'or');
y2=ylabel('Fructose [g L^-^1]');
y2.Color='black';
ax = gca;
ax.YColor = 'k';
hold off

subplot(2,1,2)
yyaxis left
plot(ttspan3,c(:,3),'-b',tspan3,Data_E_cloacae_Acetoin_SI(:,1),'ob');
x1=xlabel('Time [h]');
y1=ylabel('Acetoin [g L^-^1]');
x1.Color='black';
y1.Color='black';
ax = gca;
ax.YColor = 'k';
hold on
yyaxis right
plot(ttspan3,c(:,4),'-g',tspan3,Data_E_cloacae_zweidreiButandiol_SI(:,1),'og');
y2=ylabel('2,3-Butandiol[g L^-^1]');
y2.Color='black';
ax = gca;
ax.YColor = 'k';
hold off

xlswrite(out_file,(c(:,1)-0.0632)./0.6416,'Fit Heterotroph','L4'); %OD
xlswrite(out_file,c(:,2)./Fructose.*1000,'Fit Heterotroph','M4'); % Fructose mM
xlswrite(out_file,c(:,3)./Acetoin.*1000,'Fit Heterotroph','N4'); % Acetoin mM
xlswrite(out_file,c(:,4)./zweidreiButandiol.*1000,'Fit Heterotroph','O4'); % Butandiol mM
xlswrite(out_file,Results_Ec0(3,2:end),'Fit Heterotroph','Q10'); %paropt

tspan3 = Data_E_cloacae_Time(:,1);

[t,c] = ode15s(@DGL,tspan3,Ec0,'',Results_Ec0(i,2:end));

Ec_ODE_R2 = [ c(:,1), c(:,2), c(:,3), c(:,4)];

R2_Ec_BTM = 1-(sum((Data_E_cloacae_BTM(:,1)-c(:,1)).^2)./(sum((Data_E_cloacae_BTM(:,1)-mean(Data_E_cloacae_BTM(:,1))).^2)));
R2_Ec_S = 1-(sum((Data_E_cloacae_Fructose_SI(:,1)-c(:,2)).^2)./(sum((Data_E_cloacae_Fructose_SI(:,1)-mean(Data_E_cloacae_Fructose_SI(:,1))).^2)));
R2_Ec_Ac = 1-(sum((Data_E_cloacae_Acetoin_SI(:,1)-c(:,3)).^2)./(sum((Data_E_cloacae_Acetoin_SI(:,1)-mean(Data_E_cloacae_Acetoin_SI(:,1))).^2)));
R2_Ec_Bu = 1-(sum((Data_E_cloacae_zweidreiButandiol_SI(:,1)-c(:,4)).^2)./(sum((Data_E_cloacae_zweidreiButandiol_SI(:,1)-mean(Data_E_cloacae_zweidreiButandiol_SI(:,1))).^2)));

R2_Ec_overview = [R2_Ec_BTM R2_Ec_S R2_Ec_Ac R2_Ec_Bu];

R2_results = [R2_Kp_overview; R2_Ka_overview; R2_Ec_overview];

xlswrite(out_file,R2_Ec_overview(:,:),'Fit Heterotroph','Q6');

end
end

## Parameterfiting

function J=objective(par,i,inputdata,Data_K_pneumoniae_BTM,Data_K_pneumoniae_Time,Data_K_pneumoniae_Fructose_SI,Data_K_pneumoniae_Acetoin_SI,Data_K_pneumoniae_zweidreiButandiol_SI,...
 Data_K_aerogenes_BTM,Data_K_aerogenes_Time,Data_K_aerogenes_Fructose_SI,Data_K_aerogenes_Acetoin_SI,Data_K_aerogenes_zweidreiButandiol_SI,...
 Data_E_cloacae_BTM,Data_E_cloacae_Time,Data_E_cloacae_Fructose_SI,Data_E_cloacae_Acetoin_SI,Data_E_cloacae_zweidreiButandiol_SI)

if i == 1
 % Choose weighting
weight = [20;...% weighting viable cell densitiy
 1;... % weighting Substrate
 2;... % weighting Acetoin
 15];... % weighting Butandiol

% options=odeset('reltol',1E-08,'abstol',1E-08,'maxstep',0.1,'normcontrol','on');
tspan = Data_K_pneumoniae_Time(:,1);
[~,c] = ode15s(@DGL,tspan,inputdata,'',par);

% evaluation criterion from the weighting sum of least squares
J = sum(weight(1)*sum(abs(c(:,1)-Data_K_pneumoniae_BTM(:,1)).^2)...
 +weight(2)*sum(abs(c(:,2)-Data_K_pneumoniae_Fructose_SI(:,1)).^2)...
 +weight(3)*sum(abs(c(:,3)-Data_K_pneumoniae_Acetoin_SI(:,1)).^2)...
 +weight(4)*sum(abs(c(:,4)-Data_K_pneumoniae_zweidreiButandiol_SI(:,1)).^2));

elseif i== 2
 % Choose weighting
weight = [20;...% weighting viable cell densitiy
 1;... % weighting Substrate
 10;... % weighting Acetoin
 10];... % weighting Butandiol

% options=odeset('reltol',1E-16,'abstol',1E-16,'maxstep',0.1,'normcontrol','on');
tspan = Data_K_aerogenes_Time(:,1);
[~,c] = ode15s(@DGL,tspan,inputdata,'',par);

% evaluation criterion from the weighting sum of least squares
J = sum(weight(1)*sum(abs(c(:,1)-Data_K_aerogenes_BTM(:,1)).^2)...
 +weight(2)*sum(abs(c(:,2)-Data_K_aerogenes_Fructose_SI(:,1)).^2)...
 +weight(3)*sum(abs(c(:,3)-Data_K_aerogenes_Acetoin_SI(:,1)).^2)...
 +weight(4)*sum(abs(c(:,4)-Data_K_aerogenes_zweidreiButandiol_SI(:,1)).^2));

elseif i== 3
 % Choose weighting
weight = [60;...% weighting viable cell densitiy
 2;... % weighting Substrate
 2;... % weighting Acetoin
 15];... % weighting Butandiol

% options=odeset('reltol',1E-16,'abstol',1E-16,'maxstep',0.1,'normcontrol','on');
tspan = Data_E_cloacae_Time(:,1);
[~,c] = ode15s(@DGL,tspan,inputdata,'',par);

% evaluation criterion from the weighting sum of least squares
J = sum(weight(1)*sum(abs(c(:,1)-Data_E_cloacae_BTM(:,1)).^2)...
 +weight(2)*sum(abs(c(:,2)-Data_E_cloacae_Fructose_SI(:,1)).^2)...
 +weight(3)*sum(abs(c(:,3)-Data_E_cloacae_Acetoin_SI(:,1)).^2)...
 +weight(4)*sum(abs(c(:,4)-Data_E_cloacae_zweidreiButandiol_SI(:,1)).^2));

end
end

## ODE

function dcdt= DGL(t,c,par)

%renaming variables
X=c(1);
S_e=c(2);
PrA=c(3);
PrB=c(4);

mumax = par(1);
Y_xS = par(2);
Y_PxA = par(3);
Y_PxB = par(4);
Y_PAB = par(5);
Ks = par(6);
mudmin= par(7);
mudmax = par(8);
qBmax = par(9);
KSB= par(10);
Smin = par(11);
t_lag = par(12);
KIB = par(13);

%define other equations
mu=mumax*(S_e/(Ks+S_e))*(1-exp(-t/t_lag)); %
mud =mudmin+mudmax*Ks/(S_e+Ks);

qs= mu/Y_xS; %g/g h
qpA = mu/Y_PxA;
qpB = mu/Y_PxB;
qpAB = Y_PAB*qBmax*PrB/(PrB+KSB);

% mass balance
dcdt(1,1)=(mu-mud)*X; %dxdt [g biomass/L h]
dcdt(2,1)=-qs*X; %dFructose/dt [g/L h]
dcdt(3,1)=qpA*X; % dAcetoin/dt [g/L h]
dcdt(4,1)=qpB*X; % dButandiol/dt [g/L h]

if dcdt(4,1)>=10/1000*90.121 %butandiol inihibtion from 10mmol
mu=mumax*(S_e/(Ks+S_e))*(1-exp(-t/t_lag))*(KIB/(KIB+PrB)); %
mud =mudmin+mudmax*Ks/(S_e+Ks)*(PrB/(PrB*KSB));
end

if dcdt(2,1) <= Smin %
dcdt(1,1)=(mu-mud)*X; %dxdt [g biomass/L h]
dcdt(2,1)=-qs*X; %dFructose/dt [g/L h]
dcdt(3,1)=qpA*X + qpAB*X; %dAcetoin/dt [g/L h]
dcdt(4,1)=qpB*X - qpAB*X; % dButandiol/dt [g/L h]
end

if dcdt(2,1) <= Smin && dcdt(4,1)>=10/1000*90.121 %butandiol inihibtion from 10mmol/L and no substrate
mu=mumax*(S_e/(Ks+S_e))*(1-exp(-t/t_lag))*(KIB/(KIB+PrB)); %
mud =mudmin+mudmax*Ks/(S_e+Ks)*(PrB/(PrB*KSB));

dcdt(1,1)=(mu-mud)*X; %dxdt [g biomass/L h]
dcdt(2,1)=-qs*X; %dFructose/dt [g/L h]
dcdt(3,1)=qpA*X + qpAB*X; %dAcetoin/dt [g/L h]
dcdt(4,1)=qpB*X - qpAB*X; % dButandiol/dt [g/L h]
end
end

[*Published with MATLAB® R2020b*](https://www.mathworks.com/products/matlab)

**Autotroph Script**

clc
clear all
close all

% Data imported from Excel
budC_cloac_Time_1=xlsread('Autotroph.xlsx','budC(cloac)','E2:E14'); %h
budC_cloac_OD=xlsread('Autotroph.xlsx','budC(cloac)','I2:I14'); %OD600
budC_cloac_X = (0.6417 * budC_cloac_OD + 0.0632); %g/l
budC_cloac_Acetoin=xlsread('Autotroph.xlsx','budC(cloac)','AM2:AM14'); %g/l
budC_cloac_Butandiol=xlsread('Autotroph.xlsx','budC(cloac)','AN2:AN14'); %g/l
budC_cloac_Time_2=xlsread('Autotroph.xlsx','budC(cloac)','B38:B42'); %h
budC_cloac_CO2=xlsread('Autotroph.xlsx','budC(cloac)','C38:C42'); %
budC_cloac_CO2_calc = budC_cloac_CO2 / 100;
budC_cloac_O2=xlsread('Autotroph.xlsx','budC(cloac)','G38:G42'); %
budC_cloac_O2_calc = budC_cloac_O2 / 100;
budC_cloac_H2=xlsread('Autotroph.xlsx','budC(cloac)','E38:E42'); %
budC_cloac_H2_calc = budC_cloac_H2 / 100;

budC_cloac_Time_3=xlsread('Autotroph.xlsx','budC(cloac)','AU2:AU11'); %h
budC_cloac_Time_4=xlsread('Autotroph.xlsx','budC(cloac)','AW2:AW10'); %h

budC_cloac_CO2_new=xlsread('Autotroph.xlsx','budC(cloac)','AT2:AT11'); %
budC_cloac_CO2_calc_new= budC_cloac_CO2_new / 100;
budC_cloac_O2_new=xlsread('Autotroph.xlsx','budC(cloac)','AX2:AX10'); %
budC_cloac_O2_calc_new = budC_cloac_O2_new / 100;
budC_cloac_H2_new=xlsread('Autotroph.xlsx','budC(cloac)','AV2:AV10'); %
budC_cloac_H2_calc_new = budC_cloac_H2_new / 100;

T1 = budC_cloac_Time_1;
OD = budC_cloac_OD;
X = budC_cloac_X;
A = budC_cloac_Acetoin;
B = budC_cloac_Butandiol;
T2 = budC_cloac_Time_2;
CO2_calc = budC_cloac_CO2_calc;
O2_calc = budC_cloac_O2_calc;
H2_calc = budC_cloac_H2_calc;

mumax = 0.02; %1 % Maximum specific growth rate(1/h)
mudmin = 0.001; %2 %Minimum specific death rate (1/h)
mudmax = 0.02; %3 %Maximum specific death rate (1/h)
KLa = 150; %4 %Mass transfer coefficient for shaking flasks (1/h)
K_O2 = 0.00003125; %5 % (Monod constant) Half-saturation constant for O2 (in g/L) mole/L
K_CO2 = 0.0000227; %6 % (Monod constant) Half-saturation constant for CO2 (in g/L) mole/L
K_H2 = 0.0005; %7 % (Monod constant) Half-saturation constant for H2 (in g/L) mole/L
K_B = 0.005; %8 (Monod constant) Half-saturation constant butandiol (g/l)
q_A_d_max = 0.00001; %9 Maximum specific consumption rate of Acetoin by biomass after the depletion of CO2 (g acetoin/g biomass)
Y_AB = 0.97; %10 Yield coefficient of Acetoin converted back from Butandiol (g acetoin/g Butanediol)
K1_A = 0.005; %11 (Monod constant) Half-saturation constant acetoin during first forward reaction after co2 depletion (g/l)
Y_BA_r = 1.02; %12 Yield coefficient of Butandiol converted from Acetoin (g butandiol/g acetoin)
q_B_upmax = 0.00001; %13 Maximum specific consumption rate of butandiol by biomass after the depletion of CO2 with still oxygen (g butandiol / g biomass)
Y_XA = 0.0005; %14 Yield coefficient of biomass produced acetoin (g biomass/g acetoin)
Y_XB = 0.005; %15 Yield coefficient of biomass produced butandiol (g biomass/g butandiol)
Y_XCO2 = 0.005; %16 Yield coefficient of biomass per CO2 (g biomass/mole CO2)
Y_XO2 = 0.005; %17 Yield coefficient of biomass per O2 (g biomass/mole O2)
Y_XH2 = 0.005; %18 Yield coefficient of biomass per H2 (g biomass/mole H2)
t_lag = 2; %19 time term for lag phase and delayed grwoth or production (h)
q_A_d_max_end = 0.00001; %20 Maximum specific consumption rate of acetoin by biomass after the depletion of CO2 and O2 (g acetoin / g biomass)
K2_A = 0.005; % 21 (Monod constant) Half-saturation constant acetoin after co2 and o2 depletion (g/l)
Y_BA_r_end = 1.02; %22 Yield coefficient of Butandiol converted from Acetoin after co2 and o2 depletion (g butandiol/g acetoin)


%molare Massen
Acetoin = 88.11; %g/mol
zweidreiButandiol = 90.14; %g/mol
CO2 = 44.01; %g/mol
H2 = 2;%g/mol
O2 = 32;%g/mol

%Gas constants and calculations
T = 303.15; %Temperature [K]
R = 8314.47; %Gas constant[Pa L moL-1 K-1]
P =101325; %Pressure [Pa]
T_regular = 298.15; % K

Hatm_CO2 = 3.4e-2; %Henry constant for CO2 [mol/L.atm]
Hatm_O2 = 1.3e-3; %Henry constant for O2 [mol/L.atm]
Hatm_H2 = 7.8e-4; %Henry constant for H2 [mol/L.atm]

c_co2 = 2400; %constant for temperature dependent henry constant
c_o2 = 1700; %constant for temperature dependent henry constant
c_h2 = 500; %constant for temperature dependent henry constant

Hatm_CO2_op = Hatm_CO2*exp(c_co2*(1/T-1/T_regular)); % henry constant at 30°C
Hatm_O2_op = Hatm_O2*exp(c_o2*(1/T-1/T_regular)); % henry constant at 30°C
Hatm_H2_op = Hatm_H2*exp(c_h2*(1/T-1/T_regular)); % henry constant at 30°C

He_CO2 = Hatm_CO2_op/P; %Henry constant for CO2 [mol/L.Pa]
He_O2 = Hatm_O2_op/P; %Henry constant for O2 [mol/L.Pa]
He_H2 = Hatm_H2_op/P; %Henry constant for H2 [mol/L.Pa]

%Gases initial (percentage) in air
YO2_0=O2_calc(1,1); %initial proportion oxygen in gas (%)
YCO2_0=CO2_calc(1,1); %initial proportion CO2 in gas (%)
YH2_0=H2_calc(1,1); %initial proportion H2 in gas (%)

%Initial Gases concentrations in liquid (mol/L)
 cCO2_L_0 = YCO2_0*P*He_CO2;
 cO2_L_0 = YO2_0*P*He_O2;
 cH2_L_0 = YH2_0*P*He_H2;

% Defines intitial values from data and guessed parameters
ICs = [X(1,1), A(1,1), B(1,1), CO2_calc(1,1),O2_calc(1,1),H2_calc(1,1), cCO2_L_0,cO2_L_0, cH2_L_0]; %Initial conditions

%Parameters chosen by hand for curve fitting and optimization
par0 = [mumax, mudmin, mudmax, KLa,K_O2, K_CO2, K_H2, K_B,q_A_d_max,Y_AB,K1_A,Y_BA_r,q_B_upmax,Y_XA,Y_XB,Y_XCO2,Y_XO2,Y_XH2, t_lag,q_A_d_max_end,K2_A,Y_BA_r_end];

% Lower bounds
lowerBounds = [0.001, 0.0001, 0.001, 130, 0.00001, 0.00001, 0.00001, 0.001, 0, 0.8, 0.001, 0.8, 0, 0.0001, 0.0001, 0.0001, 0.0001, 0.0001, 0 ,0, 0.001, 0.8 ];
% Upper bound
upperBounds = [0.1, 0.01, 0.2, 180, 1, 1, 1, 8, 4, 1.2, 8, 1.2, 4, 40, 40, 10, 10, 10, 200, 4, 8, 1.2];

## Use fmincon

options = optimset('Display', 'Iter', 'MaxIter', 200, 'TolFun', 1e-6, 'TolX', 1e-6);

paropt = fmincon(@objective, par0, [], [], [], [], lowerBounds, upperBounds, [], options, ICs, T1, X, A, B, T2, CO2_calc, O2_calc, H2_calc,budC_cloac_Time_3,budC_cloac_Time_4,budC_cloac_CO2_calc_new,budC_cloac_O2_calc_new,budC_cloac_H2_calc_new);
Results_par = paropt; % Safe estimated parameters

## Final simulation

tspan1 = T1(:,1);
ttspan1 = 0:0.5:max(tspan1 + 4);
tspan2 = T2(:,1);
ttspan2 = 0:0.5:max(tspan2 + 4);

options=odeset('reltol',1E-4,'abstol',1E-4);
[t,c] = ode15s(@DGL,ttspan2,ICs, options, Results_par);

OD_graph = (c(:,1) - 0.0632)/0.6417;
figure('Name','budC(cloac)','NumberTitle','off');
subplot(3,1,1)
plot(tspan1, OD(:,1), 'ob');
hold on
plot(t, OD_graph , '-r', 'LineWidth', 1);
hold off
x=xlabel('Time [h]');
y=ylabel('OD_{600}');
legend('Lab','Model');
x.Color='black';
y.Color='black';
ax = gca;
ax.YColor = 'k';

subplot(3,1,2)
plot(t,c(:,2),'-r',tspan1,A(:,1),'or');
x1=xlabel('Time [h]');
y1=ylabel('Acetoin [g L^-^1]');
legend('Acetoin', 'exp. Data Acetoin','Location','southeast','NumColumns',2);
y1.Color='black';
x1.Color='black';
ax = gca;
ax.YColor = 'k';
hold off

subplot(3,1,3)
plot(t,c(:,3),'-b',tspan1,B(:,1),'ob');
x1=xlabel('Time [h]');
y1=ylabel('Butandiol [g L^-^1]');
legend('Butandiol','exp. Data Butandiol','Location','southeast','NumColumns',2);
x1.Color='black';
y1.Color='black';
ax = gca;
ax.YColor = 'k';
hold off

figure('Name','budC(cloac)','NumberTitle','off');
subplot (3,1,1)
plot(t,c(:,4).*100,'-b',tspan2,CO2_calc(:,1).*100,'og');
legend('CO2','exp. Data CO2','Location','southeast','NumColumns',2);
y1=ylabel('CO2 [%]');
x1=xlabel('Time [h]');
x1.Color='black';
y1.Color='black';
ax = gca;
ax.YColor = 'k';
hold off

subplot (3,1,2)
plot(t,c(:,5).*100,'-b',tspan2,O2_calc(:,1).*100,'og');
legend('O2','exp. Data CO2','Location','southeast','NumColumns',2);
y1=ylabel('O2 [%]');
x1=xlabel('Time [h]');
x1.Color='black';
y1.Color='black';
ax = gca;
ax.YColor = 'k';
hold off

subplot (3,1,3)
plot(t,c(:,6).*100,'-b',tspan2,H2_calc(:,1).*100,'og');
legend('H2','exp. Data CO2','Location','southeast','NumColumns',2);
y1=ylabel('H2 [%]');
x1=xlabel('Time [h]');
x1.Color='black';
y1.Color='black';
ax = gca;
ax.YColor = 'k';
hold off

figure('Name','budC(cloac)','NumberTitle','off');
subplot (3,1,1)
plot(t,c(:,7),'-b');
legend('CO2','Location','southeast','NumColumns',2);
y1=ylabel('CO2 [mole L^-^1]');
x1=xlabel('Time [h]');
x1.Color='black';
y1.Color='black';
ax = gca;
ax.YColor = 'k';
hold off

subplot (3,1,2)
plot(t,c(:,8),'-b');
legend('O2 in liquid','Location','southeast','NumColumns',2);
y1=ylabel('O2 in liquid [mole L^-^1]');
x1=xlabel('Time [h]');
x1.Color='black';
y1.Color='black';
ax = gca;
ax.YColor = 'k';
hold off

subplot (3,1,3)
legend('H2 in liquid','Location','southeast','NumColumns',2);
plot(t,c(:,9),'-b');
y1=ylabel('H2 [mole L^-^1]');
x1=xlabel('Time [h]');
x1.Color='black';
y1.Color='black';
ax = gca;
ax.YColor = 'k';
hold off

%
TT1 = 2 * (T1 + 1);
TT1 = round(TT1);

TT2 = 2 * (T2 +1) ;
TT2 = round(TT2);

lab1 = OD;
lab2 = A;
lab3 = B;
lab4 = CO2_calc;
lab5 = O2_calc;
lab6 = H2_calc;

model1 = OD_graph(TT1 ,1);
model2 = c(TT1,2);
model3 = c(TT1,3);
model4 = c(TT2,4);
model5 = c(TT2,5);
model6 = c(TT2,6);

% Calculate the squared error
SQ1 = sum((lab1 - model1).^2);
SQ2 = sum((lab2 - model2).^2);
SQ3 = sum((lab3 - model3).^2);
SQ4 = sum((lab4 - model4).^2);
SQ5 = sum((lab5 - model5).^2);
SQ6 = sum((lab6 - model6).^2);

%
MSQ1 = sum((lab1 - mean(lab1)).^2);
MSQ2 = sum((lab2 - mean(lab2)).^2);
MSQ3 = sum((lab3 - mean(lab3)).^2);
MSQ4 = sum((lab4 - mean(lab4)).^2);
MSQ5 = sum((lab5 - mean(lab5)).^2);
MSQ6 = sum((lab6 - mean(lab6)).^2);

%
rmse1 = 1 - SQ1/MSQ1;
rmse2 = 1 - SQ2/MSQ2;
rmse3 = 1 - SQ3/MSQ3;
rmse4 = 1 - SQ4/MSQ4;
rmse5 = 1 - SQ5/MSQ5;
rmse6 = 1 - SQ6/MSQ6;

RMSE = [rmse1 rmse2 rmse3 rmse4 rmse5 rmse6 ];
% Display the RMSE
disp('RMSE: ');
disp(RMSE);

%molare Massen
Acetoin = 88.11; %g/mol
zweidreiButandiol = 90.14; %g/mol

out_file = 'Autotrophfit';
xlswrite(out_file,OD_graph(:,1),'Fit1','B8');%OD
xlswrite(out_file,c(:,2)./Acetoin.*1000,'Fit1','C8'); % acetoin mM
xlswrite(out_file,c(:,3)./zweidreiButandiol.*1000,'Fit1','D8'); % butandiol mM
xlswrite(out_file,c(:,4).*100,'Fit1','E8'); %CO2gas
xlswrite(out_file,c(:,5).*100,'Fit1','F8');%O2gas
xlswrite(out_file,c(:,6).*100,'Fit1','G8');%H2gas
xlswrite(out_file,c(:,7),'Fit1','H8');%CO2 liquid
xlswrite(out_file,c(:,8),'Fit1','I8');%O2 liquid
xlswrite(out_file,c(:,9),'Fit1','J8');%H2 liquid
xlswrite(out_file,t(:,1),'Fit1','A8');%Time

xlswrite(out_file,paropt(:,:),'Fit1','H1'); %
xlswrite(out_file,rmse1,'Fit1','A1'); %
xlswrite(out_file,rmse2,'Fit1','B1'); %
xlswrite(out_file,rmse3,'Fit1','C1'); %
xlswrite(out_file,rmse4,'Fit1','D1'); %
xlswrite(out_file,rmse5,'Fit1','E1'); %
xlswrite(out_file,rmse6,'Fit1','F1'); %

## Objective

function J_final=objective(par,ICs,T1, X, A, B, T2,CO2_calc, O2_calc, H2_calc,budC_cloac_Time_3,budC_cloac_Time_4,budC_cloac_CO2_calc_new,budC_cloac_O2_calc_new,budC_cloac_H2_calc_new)

weight = [1;...% weighting viable cell densitiy
 8;... % weighting Acetoin
 6;... % weighting Butandiol
 800;... % weighting CO2
 60;... % Weighting O2
 6];... % Weighting H2


% Solves the DGL System with starting values
options=odeset('reltol',1E-09,'abstol',1E-09,'maxstep',0.1,'normcontrol','on');

t_all = T1;
tspan = 0:0.1:max(T1)+1;

size_vec = size(T1,1);

[~,c] = ode15s(@DGL,tspan,ICs,options,par);

% Concentration profile over time
c_1 = zeros(size_vec,1);
c_2 = zeros(size_vec,1);
c_3 = zeros(size_vec,1);

[~,LocAllB] = ismembertol(tspan',t_all(1:end,1),0.0001);
[~,LocAllB2] = ismembertol(tspan',t_all(1:end,1),0.0001);
[~,LocAllB3] = ismembertol(tspan',t_all(1:end,1),0.0001);

I = zeros(length(t_all(1:end,1)),3);

for timing = 1:size(t_all(1:end,1),1)

 I(timing,1) = find(timing==LocAllB);
 I(timing,2) = find(timing==LocAllB2);
 I(timing,3) = find(timing==LocAllB3);
end

for j = 1:size(t_all,1)
 c_1(j,:) = c(I(j,1),1);
 c_2(j,:) = c(I(j,2),2);
 c_3(j,:) = c(I(j,3),3);
end

% Solves the DGL System with starting values
options=odeset('reltol',1E-09,'abstol',1E-09,'maxstep',0.1,'normcontrol','on');

t_all_2 = round(budC_cloac_Time_3,1);
t_all_3 = round(budC_cloac_Time_4,1);
tspan_2 = 0:0.1:max(budC_cloac_Time_3)+1;

size_vec_2 = size(budC_cloac_Time_3,1);
size_vec_3 = size(budC_cloac_Time_4,1);

[~,c] = ode15s(@DGL,tspan_2,ICs,options,par);

% Concentration profile over time
c_4 = zeros(size_vec_2,1);
c_5 = zeros(size_vec_3,1);
c_6 = zeros(size_vec_3,1);

[~,LocAllB4] = ismembertol(tspan_2',t_all_2(1:end,1),0.0001);
[~,LocAllB5] = ismembertol(tspan_2',t_all_3(1:end,1),0.0001);
[~,LocAllB6] = ismembertol(tspan_2',t_all_3(1:end,1),0.0001);

I2 = zeros(length(t_all_2(1:end,1)),3);
I3 = zeros(length(t_all_3(1:end,1)),3);

for timing = 1:size(t_all_2(1:end,1),1)

 I2(timing,1) = find(timing==LocAllB4);
end

for j = 1:size(t_all_2,1)
 c_4(j,:) = c(I2(j,1),4);

end

for timing = 1:size(t_all_3(1:end,1),1)

 I3(timing,1) = find(timing==LocAllB5);
 I3(timing,2) = find(timing==LocAllB6);
end

for j = 1:size(t_all_3,1)

 c_5(j,:) = c(I3(j,1),5);
 c_6(j,:) = c(I3(j,2),6);

end

 J_final = sum(weight(1)*sum(abs(c_1(:,1)- X(:,1)).^2)...
 +weight(2)*sum(abs(c_2(:,1)- A(:,1)).^2)...
 +weight(3)*sum(abs(c_3(:,1)- B(:,1)).^2)...
 +weight(4)*sum(abs(c_4(:,1)- budC_cloac_CO2_calc_new(:,1)).^2)...
 +weight(5)*sum(abs(c_5(:,1)- budC_cloac_O2_calc_new(:,1)).^2)...
 +weight(6)*sum(abs(c_6(:,1)- budC_cloac_H2_calc_new(:,1)).^2));

end

## ODEs for Batch reactor

function dcdt= DGL(t,c,par)

%Gas constants and calculations
T = 303.15; %Temperature [K]
R = 8314.47; %Gas constant[Pa L moL-1 K-1]
P =101325; %Pressure [Pa]
T_regular = 298.15; % K

Hatm_CO2 = 3.4e-2; %Henry constant for CO2 [mol/L.atm]
Hatm_O2 = 1.3e-3; %Henry constant for O2 [mol/L.atm]
Hatm_H2 = 7.8e-4; %Henry constant for H2 [mol/L.atm]

c_co2 = 2400; %constant for temperature dependent henry constant
c_o2 = 1700; %constant for temperature dependent henry constant
c_h2 = 500; %constant for temperature dependent henry constant

Hatm_CO2_op = Hatm_CO2*exp(c_co2*(1/T-1/T_regular)); % henry constant at 30°C
Hatm_O2_op = Hatm_O2*exp(c_o2*(1/T-1/T_regular)); % henry constant at 30°C
Hatm_H2_op = Hatm_H2*exp(c_h2*(1/T-1/T_regular)); % henry constant at 30°C

He_CO2 = Hatm_CO2_op/P; %Henry constant for CO2 [mol/L.Pa]
He_O2 = Hatm_O2_op/P; %Henry constant for O2 [mol/L.Pa]
He_H2 = Hatm_H2_op/P; %Henry constant for H2 [mol/L.Pa]

% constant parameters
VL =0.05; %liquid volume [L]
VG = 0.95; % Gas volume [L]

%Renaming variables for easier overview
X=c(1); % g/l
PrA=c(2); % g/l
PrB=c(3); % g/l
YCO2= c(4); % %
YO2 = c(5); % %
YH2 = c(6); % %
cCO2_L = c(7); % moles/l
cO2_L = c(8); % moles/l
cH2_L = c(9); % moles/l

%Parameters after optimization
mumax = par(1);
mudmin = par(2);
mudmax = par(3);
KLa = par(4);
K_O2 = par(5);
K_CO2 = par(6);
K_H2 = par(7);
K_B = par(8);
q_A_d_max = par(9);
Y_AB = par(10);
K1_A = par(11);
Y_BA_r = par(12);
q_B_upmax = par(13);
Y_XA = par(14);
Y_XB = par(15);
Y_XCO2 = par(16);
Y_XO2 = par(17);
Y_XH2 = par(18);
t_lag = par(19);
q_A_d_max_end = par(20);
K2_A = par(21);
Y_BA_r_end = par(22);

mu = mumax* (cCO2_L /(K_CO2+cCO2_L))*(cO2_L/(K_O2+cO2_L)) *(cH2_L/(K_H2+cH2_L)) ; %Specific growth rate of biomass (1/h)
mud =mudmin + mudmax *(cCO2_L /(K_CO2+cCO2_L))*(K_O2/(K_O2+cO2_L)) * (K_H2/(K_H2+cH2_L)); %Specific death rate of biomass(1/h)

q_A_P = mu/Y_XA;
q_B = mu/Y_XB;

dcdt(1,1)= (mu-mud)*X ; %dxdt [g biomass/L h]
dcdt(2,1)= (q_A_P) * X ; % dAcetoin/dt [g/L h]
dcdt(3,1)= (q_B)* X ; % dButandiol/dt [g/L h]
dcdt(4,1)= - KLa * VL/VG * R*T/P * (YCO2*He_CO2*P - cCO2_L); % dCO2/dt in gas [%CO2/h]
dcdt(5,1)= - KLa * VL/VG * R*T/P * (YO2*He_O2*P - cO2_L); % dO2/dt in gas [%O2/h]
dcdt(6,1)= - KLa * VL/VG * R*T/P * (YH2*He_H2*P - cH2_L); % dH2/dt in gas [%H2/h]
dcdt(7,1)= KLa*(YCO2*He_CO2*P - cCO2_L)- X*mu/Y_XCO2; % dCO2/dt in liquid [mole/L h]
dcdt(8,1)= KLa*(YO2*He_O2*P - cO2_L)- X*mu/Y_XO2; % dO2/dt in liquid [mole/L h]
dcdt(9,1)= KLa*(YH2*He_H2*P - cH2_L)- X*mu/Y_XH2 ; % dH2/dt in liquid [mole/L h]

if t>22 && t<=41
mu = mumax* (cCO2_L /(K_CO2+cCO2_L))*(cO2_L/(K_O2+cO2_L)) *(cH2_L/(K_H2+cH2_L)) ; %Specific growth rate of biomass (1/h)

mu1= mumax*(cO2_L/(K_O2+cO2_L)) *(cH2_L/(K_H2+cH2_L)) ; %Specific growth rate of biomass (1/h)
mud1 =mudmin + mudmax *(K_O2/(K_O2+cO2_L)) * (K_H2/(K_H2+cH2_L)); %Specific death rate of biomass(1/h)

q_A_P = mu/Y_XA;
q_B = mu/Y_XB;
q_B_r = q_B_upmax*PrB/(PrB+K_B) ;
q_B_rA = q_B_r*Y_AB ;

dcdt(1,1)= (mu1-mud1)*X ; %dxdt [g biomass/L h]
dcdt(2,1)= (q_A_P+q_B_rA) * X ; % dAcetoin/dt [g/L h]
dcdt(3,1)= (q_B-q_B_r)* X ; % dButandiol/dt [g/L h
dcdt(4,1)= - KLa * VL/VG * R*T/P * (YCO2*He_CO2*P - cCO2_L); % dCO2/dt in gas [%CO2/h]
dcdt(5,1)= - KLa * VL/VG * R*T/P * (YO2*He_O2*P - cO2_L); % dO2/dt in gas [%O2/h]
dcdt(6,1)= - KLa * VL/VG * R*T/P * (YH2*He_H2*P - cH2_L); % dH2/dt in gas [%H2/h]
dcdt(7,1)= KLa*(YCO2*He_CO2*P - cCO2_L)- X*mu/Y_XCO2; % dCO2/dt in liquid [mole/L h]
dcdt(8,1)= KLa*(YO2*He_O2*P - cO2_L)- X*mu1/Y_XO2; % dO2/dt in liquid [mole/L h]
dcdt(9,1)= KLa*(YH2*He_H2*P - cH2_L)- X*mu1/Y_XH2 ; % dH2/dt in liquid [mole/L h]

elseif t>41
mu = mumax* (cCO2_L /(K_CO2+cCO2_L))*(cO2_L/(K_O2+cO2_L)) *(cH2_L/(K_H2+cH2_L)) ; %Specific growth rate of biomass (1/h)

mu1= mumax*(cO2_L/(K_O2+cO2_L)) *(cH2_L/(K_H2+cH2_L)) ; %Specific growth rate of biomass (1/h)
mud1 =mudmin + mudmax *(K_O2/(K_O2+cO2_L)) * (K_H2/(K_H2+cH2_L)); %Specific death rate of biomass(1/h)

q_A_P = mu/Y_XA;
q_B = mu/Y_XB;
q_A_d_end = q_A_d_max_end * PrA/(PrA + K2_A) ;
q_A_d_r_end = q_A_d_end * Y_BA_r_end;

dcdt(1,1)= (mu1-mud1)*X ; %dxdt [g biomass/L h]
dcdt(2,1)= (q_A_P-q_A_d_end) * X ; % dAcetoin/dt [g/L h]
dcdt(3,1)= (q_B+q_A_d_r_end)* X ; % dButandiol/dt [g/L h
dcdt(4,1)= - KLa * VL/VG * R*T/P * (YCO2*He_CO2*P - cCO2_L); % dCO2/dt in gas [%CO2/h]
dcdt(5,1)= - KLa * VL/VG * R*T/P * (YO2*He_O2*P - cO2_L); % dO2/dt in gas [%O2/h]
dcdt(6,1)= - KLa * VL/VG * R*T/P * (YH2*He_H2*P - cH2_L); % dH2/dt in gas [%H2/h]
dcdt(7,1)= KLa*(YCO2*He_CO2*P - cCO2_L)- X*mu/Y_XCO2; % dCO2/dt in liquid [mole/L h]
dcdt(8,1)= KLa*(YO2*He_O2*P - cO2_L)- X*mu1/Y_XO2; % dO2/dt in liquid [mole/L h]
dcdt(9,1)= KLa*(YH2*He_H2*P - cH2_L)- X*mu1/Y_XH2 ; % dH2/dt in liquid [mole/L h]
end
end

clc
clear all
close all

 % Data imported from Excel
cag_cloac_Time_1=xlsread('Autotroph.xlsx','cag(cloac)','E2:E14'); %h
cag_cloac_OD=xlsread('Autotroph.xlsx','cag(cloac)','I2:I14'); %OD600
cag_cloac_X = (0.6417 * cag_cloac_OD + 0.0632); %g/l
cag_cloac_Acetoin=xlsread('Autotroph.xlsx','cag(cloac)','AM2:AM14'); %g/l
cag_cloac_Butandiol=xlsread('Autotroph.xlsx','cag(cloac)','AN2:AN14'); %g/l
cag_cloac_Time_2=xlsread('Autotroph.xlsx','cag(cloac)','B38:b42'); %h
cag_cloac_CO2=xlsread('Autotroph.xlsx','cag(cloac)','C38:C42'); %
cag_cloac_CO2_calc = cag_cloac_CO2 / 100;
cag_cloac_O2=xlsread('Autotroph.xlsx','cag(cloac)','G38:G42'); %
cag_cloac_O2_calc = cag_cloac_O2 / 100;
cag_cloac_H2=xlsread('Autotroph.xlsx','cag(cloac)','E38:E42'); %
cag_cloac_H2_calc = cag_cloac_H2 / 100;

cag_cloac_Time_3=xlsread('Autotroph.xlsx','cag(cloac)','AS2:AS14'); %h
cag_cloac_Time_4=xlsread('Autotroph.xlsx','cag(cloac)','AV2:AV14'); %h

cag_cloac_CO2_new=xlsread('Autotroph.xlsx','cag(cloac)','AR2:AR14'); %
cag_cloac_CO2_calc_new = cag_cloac_CO2_new / 100;
cag_cloac_O2_new=xlsread('Autotroph.xlsx','cag(cloac)','AU2:AU14'); %
cag_cloac_O2_calc_new = cag_cloac_O2_new / 100;
cag_cloac_H2_new=xlsread('Autotroph.xlsx','cag(cloac)','AX2:AX14'); %
cag_cloac_H2_calc_new = cag_cloac_H2_new / 100;

T1 = cag_cloac_Time_1;
OD = cag_cloac_OD;
X = cag_cloac_X;
A = cag_cloac_Acetoin;
B = cag_cloac_Butandiol;
T2 = cag_cloac_Time_2;
CO2_calc = cag_cloac_CO2_calc;
O2_calc = cag_cloac_O2_calc;
H2_calc = cag_cloac_H2_calc;

%Parameters chosen by hand for curve fitting and optimization
mumax = 0.02; %1 % Maximum specific growth rate(1/h)
mudmin = 0.001; %2 %Minimum specific death rate (1/h)
mudmax = 0.02; %3 %Maximum specific death rate (1/h)
KLa = 150; %4 %Mass transfer coefficient for shaking flasks (1/h)
K_O2 = 0.00003125; %5 % (Monod constant) Half-saturation constant for O2 (in g/L) mole/L
K_CO2 = 0.0000227; %6 % (Monod constant) Half-saturation constant for CO2 (in g/L) mole/L
K_H2 = 0.0005; %7 % (Monod constant) Half-saturation constant for H2 (in g/L) mole/L
K_B = 0.0005; %8 (Monod constant) Half-saturation constant butandiol (g/l)
q_A_d_max = 0.00001; %9 Maximum specific consumption rate of Acetoin by biomass after the depletion of CO2 (g acetoin/g biomass)
Y_AB = 0.97; %10 Yield coefficient of Acetoin converted back from Butandiol (g acetoin/g Butanediol)
K1_A = 0.0005; %11 (Monod constant) Half-saturation constant acetoin during first forward reaction after co2 depletion (g/l)
Y_BA_r = 1.02; %12 Yield coefficient of Butandiol converted from Acetoin (g butandiol/g acetoin)
q_B_upmax = 0.00001; %13 Maximum specific consumption rate of butandiol by biomass after the depletion of CO2 with still oxygen (g butandiol / g biomass)
Y_XA = 0.005; %14 Yield coefficient of biomass produced acetoin (g biomass/g acetoin)
Y_XB = 0.005; %15 Yield coefficient of biomass produced butandiol (g biomass/g butandiol)
Y_XCO2 = 0.005; %16 Yield coefficient of biomass per CO2 (g biomass/mole CO2)
Y_XO2 = 0.005; %17 Yield coefficient of biomass per O2 (g biomass/mole O2)
Y_XH2 = 0.005; %18 Yield coefficient of biomass per H2 (g biomass/mole H2)
t_lag = 2; %19 time term for lag phase and delayed grwoth or production (h)
q_A_d_max_end = 0.0001; %20 Maximum specific consumption rate of acetoin by biomass after the depletion of CO2 and O2 (g acetoin / g biomass)
K1_A_end = 0.0005; % 21 (Monod constant) Half-saturation constant acetoin after co2 and o2 depletion (g/l)
Y_BA_r_end = 1.02; %22 Yield coefficient of Butandiol converted from Acetoin after co2 and o2 depletion (g butandiol/g acetoin)

%molare Massen
Acetoin = 88.11; %g/mol
zweidreiButandiol = 90.14; %g/mol
CO2 = 44.01; %g/mol
H2 = 2;%g/mol
O2 = 32;%g/mol

%Gas constants and calculations
T = 303.15; %Temperature [K]
R = 8314.47; %Gas constant[Pa L moL-1 K-1]
P =101325; %Pressure [Pa]
T_regular = 298.15; % K

Hatm_CO2 = 3.4e-2; %Henry constant for CO2 [mol/L.atm]
Hatm_O2 = 1.3e-3; %Henry constant for O2 [mol/L.atm]
Hatm_H2 = 7.8e-4; %Henry constant for H2 [mol/L.atm]

c_co2 = 2400; %constant for temperature dependent henry constant
c_o2 = 1700; %constant for temperature dependent henry constant
c_h2 = 500; %constant for temperature dependent henry constant

Hatm_CO2_op = Hatm_CO2*exp(c_co2*(1/T-1/T_regular)); % henry constant at 30°C
Hatm_O2_op = Hatm_O2*exp(c_o2*(1/T-1/T_regular)); % henry constant at 30°C
Hatm_H2_op = Hatm_H2*exp(c_h2*(1/T-1/T_regular)); % henry constant at 30°C

He_CO2 = Hatm_CO2_op/P; %Henry constant for CO2 [mol/L.Pa]
He_O2 = Hatm_O2_op/P; %Henry constant for O2 [mol/L.Pa]
He_H2 = Hatm_H2_op/P; %Henry constant for H2 [mol/L.Pa]

%Gases initial (percentage) in air
YO2_0=cag_cloac_O2_calc(1,1); %initial proportion oxygen in gas (%)
YCO2_0=cag_cloac_CO2_calc(1,1); %initial proportion CO2 in gas (%)
YH2_0=cag_cloac_H2_calc(1,1); %initial proportion H2 in gas (%)

%Initial Gases concentrations in liquid (mol/L)
 cCO2_L_0 = YCO2_0*P*He_CO2;
 cO2_L_0 = YO2_0*P*He_O2;
 cH2_L_0 = YH2_0*P*He_H2;

% Defines intitial values from data and guessed parameters
ICs = [cag_cloac_X(1,1), cag_cloac_Acetoin(1,1), cag_cloac_Butandiol(1,1), cag_cloac_CO2_calc(1,1),cag_cloac_O2_calc(1,1),cag_cloac_H2_calc(1,1), cCO2_L_0,cO2_L_0, cH2_L_0];

par0 = [mumax, mudmin, mudmax, KLa,K_O2, K_CO2, K_H2, K_B,q_A_d_max,Y_AB,K1_A,Y_BA_r,q_B_upmax,Y_XA,Y_XB,Y_XCO2,Y_XO2,Y_XH2, t_lag q_A_d_max_end, K1_A_end,Y_BA_r_end];


% % Lower bounds
lowerBounds = [0.0008, 0.0001, 0.001, 130, 0.00001, 0.00001, 0.00001, 0, 0, 0.8, 0, 0.8, 0, 0, 0, 0, 0, 0, 0, 0, 0, 0.8];
% Upper bound
upperBounds = [0.2, 0.01, 0.2, 180, 1, 1, 1, 1, 10, 1.2, 9, 1.2, 2, 40, 40, 10, 10, 10, 200, 10 , 9, 1.2];

## Use fmincon

options = optimset('Display', 'Iter', 'MaxIter', 200, 'TolFun', 1e-6, 'TolX', 1e-6);

paropt = fmincon(@objective, par0, [], [], [], [], lowerBounds, upperBounds, [], options, ICs, T1, X, A, B, T2, CO2_calc, O2_calc, H2_calc,cag_cloac_Time_3,cag_cloac_Time_4,cag_cloac_CO2_calc_new,cag_cloac_O2_calc_new,cag_cloac_H2_calc_new);
Results_par = paropt; % Safe estimated parameters

## Final simulation

tspan1 = T1(:,1);
ttspan1 = 0:0.5:max(tspan1 + 4);
tspan2 = T2(:,1);
ttspan2 = 0:0.5:max(tspan2 + 4);

options=odeset('reltol',1E-4,'abstol',1E-4);
[t,c] = ode15s(@DGL,ttspan2,ICs, options, Results_par); %

OD_graph = (c(:,1) - 0.0632)/0.6417;
figure('Name','cag(cloac)','NumberTitle','off');
subplot(3,1,1)
plot(tspan1, OD(:,1), 'ob');
hold on
plot(t, OD_graph , '-r', 'LineWidth', 1);
hold off
x=xlabel('Time [h]');
y=ylabel('OD_{600}');
legend('Lab','Model');
x.Color='black';
y.Color='black';
ax = gca;
ax.YColor = 'k';

subplot(3,1,2)
plot(t,c(:,2),'-r',tspan1,A(:,1),'or');
x1=xlabel('Time [h]');
y1=ylabel('Acetoin [g L^-^1]');
legend('Acetoin', 'exp. Data Acetoin','Location','southeast','NumColumns',2);
y1.Color='black';
x1.Color='black';
ax = gca;
ax.YColor = 'k';
hold off

subplot(3,1,3)
plot(t,c(:,3),'-b',tspan1,B(:,1),'ob');
x1=xlabel('Time [h]');
y1=ylabel('Butandiol [g L^-^1]');
legend('Butandiol','exp. Data Butandiol','Location','southeast','NumColumns',2);
x1.Color='black';
y1.Color='black';
ax = gca;
ax.YColor = 'k';
hold off

figure('Name','cag(cloac)','NumberTitle','off');
subplot (3,1,1)
plot(t,c(:,4).*100,'-b',tspan2,CO2_calc(:,1).*100,'og');
legend('CO2','exp. Data CO2','Location','southeast','NumColumns',2);
y1=ylabel('CO2 [%]');
x1=xlabel('Time [h]');
x1.Color='black';
y1.Color='black';
ax = gca;
ax.YColor = 'k';
hold off

subplot (3,1,2)
plot(t,c(:,5).*100,'-b',tspan2,O2_calc(:,1).*100,'og');
legend('O2','exp. Data CO2','Location','southeast','NumColumns',2);
y1=ylabel('O2 [%]');
x1=xlabel('Time [h]');
x1.Color='black';
y1.Color='black';
ax = gca;
ax.YColor = 'k';
hold off

subplot (3,1,3)
plot(t,c(:,6).*100,'-b',tspan2,H2_calc(:,1).*100,'og');
legend('H2','exp. Data CO2','Location','southeast','NumColumns',2);
y1=ylabel('H2 [%]');
x1=xlabel('Time [h]');
x1.Color='black';
y1.Color='black';
ax = gca;
ax.YColor = 'k';
hold off

figure('Name','cag(cloac)','NumberTitle','off');
subplot (3,1,1)
plot(t,c(:,7),'-b');
legend('CO2','Location','southeast','NumColumns',2);
y1=ylabel('CO2 [mole L^-^1]');
x1=xlabel('Time [h]');
x1.Color='black';
y1.Color='black';
ax = gca;
ax.YColor = 'k';
hold off

subplot (3,1,2)
plot(t,c(:,8),'-b');
legend('O2 in liquid','Location','southeast','NumColumns',2);
y1=ylabel('O2 in liquid [mole L^-^1]');
x1=xlabel('Time [h]');
x1.Color='black';
y1.Color='black';
ax = gca;
ax.YColor = 'k';
hold off

subplot (3,1,3)
legend('H2 in liquid','Location','southeast','NumColumns',2);
plot(t,c(:,9),'-b');
y1=ylabel('H2 [mole L^-^1]');
x1=xlabel('Time [h]');
x1.Color='black';
y1.Color='black';
ax = gca;
ax.YColor = 'k';
hold off

%
TT1 = 2 * (T1 + 1);
TT1 = round(TT1);

TT2 = 2 * (T2 +1) ;
TT2 = round(TT2);

lab1 = OD;
lab2 = A;
lab3 = B;
lab4 = CO2_calc;
lab5 = O2_calc;
lab6 = H2_calc;

model1 = OD_graph(TT1 ,1);
model2 = c(TT1,2);
model3 = c(TT1,3);
model4 = c(TT2,4);
model5 = c(TT2,5);
model6 = c(TT2,6);

% Calculate the squared error
SQ1 = sum((lab1 - model1).^2);
SQ2 = sum((lab2 - model2).^2);
SQ3 = sum((lab3 - model3).^2);
SQ4 = sum((lab4 - model4).^2);
SQ5 = sum((lab5 - model5).^2);
SQ6 = sum((lab6 - model6).^2);

%
MSQ1 = sum((lab1 - mean(lab1)).^2);
MSQ2 = sum((lab2 - mean(lab2)).^2);
MSQ3 = sum((lab3 - mean(lab3)).^2);
MSQ4 = sum((lab4 - mean(lab4)).^2);
MSQ5 = sum((lab5 - mean(lab5)).^2);
MSQ6 = sum((lab6 - mean(lab6)).^2);

%
rmse1 = 1 - SQ1/MSQ1;
rmse2 = 1 - SQ2/MSQ2;
rmse3 = 1 - SQ3/MSQ3;
rmse4 = 1 - SQ4/MSQ4;
rmse5 = 1 - SQ5/MSQ5;
rmse6 = 1 - SQ6/MSQ6;

RMSE = [rmse1 rmse2 rmse3 rmse4 rmse5 rmse6 ];
% Display the RMSE
disp('RMSE: ');
disp(RMSE);

out_file = 'Autotrophfit';
xlswrite(out_file,OD_graph(:,1),'Fit1','M8');%OD
xlswrite(out_file,c(:,2)./Acetoin.*1000,'Fit1','N8'); % acetoin mM
xlswrite(out_file,c(:,3)./zweidreiButandiol.*1000,'Fit1','O8'); % butandiol mM
xlswrite(out_file,c(:,4).*100,'Fit1','P8'); %CO2gas
xlswrite(out_file,c(:,5).*100,'Fit1','Q8');%O2gas
xlswrite(out_file,c(:,6).*100,'Fit1','R8');%H2gas
xlswrite(out_file,c(:,7),'Fit1','S8');%CO2 liquid
xlswrite(out_file,c(:,8),'Fit1','T8');%O2 liquid
xlswrite(out_file,c(:,9),'Fit1','U8');%H2 liquid
xlswrite(out_file,t(:,1),'Fit1','L8');%Time

xlswrite(out_file,paropt(:,:),'Fit1','H2'); %
xlswrite(out_file,rmse1,'Fit1','A2'); %
xlswrite(out_file,rmse2,'Fit1','B2'); %
xlswrite(out_file,rmse3,'Fit1','C2'); %
xlswrite(out_file,rmse4,'Fit1','D2'); %
xlswrite(out_file,rmse5,'Fit1','E2'); %
xlswrite(out_file,rmse6,'Fit1','F2'); %

## Objective

function J_final=objective(par,ICs,T1, X, A, B, T2,CO2_calc, O2_calc, H2_calc,cag_cloac_Time_3,cag_cloac_Time_4,cag_cloac_CO2_calc_new,cag_cloac_O2_calc_new,cag_cloac_H2_calc_new)

 weight = [1;...% weighting viable cell densitiy
 8;... % weighting Acetoin
 8;... % weighting Butandiol
 600;... % weighting CO2
 180;... % Weighting O2
 18];... % Weighting H2

% Solves the DGL System with starting values
options=odeset('reltol',1E-09,'abstol',1E-09,'maxstep',0.1,'normcontrol','on');

t_all = T1;
tspan = 0:0.1:max(T1)+1;

size_vec = size(T1,1);

[~,c] = ode15s(@DGL,tspan,ICs,options,par);

% Concentration profile over time
c_1 = zeros(size_vec,1);
c_2 = zeros(size_vec,1);
c_3 = zeros(size_vec,1);

[~,LocAllB] = ismembertol(tspan',t_all(1:end,1),0.0001);
[~,LocAllB2] = ismembertol(tspan',t_all(1:end,1),0.0001);
[~,LocAllB3] = ismembertol(tspan',t_all(1:end,1),0.0001);

I = zeros(length(t_all(1:end,1)),3);

for timing = 1:size(t_all(1:end,1),1)

 I(timing,1) = find(timing==LocAllB);
 I(timing,2) = find(timing==LocAllB2);
 I(timing,3) = find(timing==LocAllB3);

end

for j = 1:size(t_all,1)
 c_1(j,:) = c(I(j,1),1);
 c_2(j,:) = c(I(j,2),2);
 c_3(j,:) = c(I(j,3),3);
end

% Solves the DGL System with starting values
options=odeset('reltol',1E-09,'abstol',1E-09,'maxstep',0.1,'normcontrol','on');

t_all_2 = round(cag_cloac_Time_3,1);
t_all_3 = round(cag_cloac_Time_4,1);
tspan_2 = 0:0.1:max(T2)+1;

size_vec_2 = size(cag_cloac_Time_3,1);
size_vec_3 = size(cag_cloac_Time_4,1);

[~,c] = ode15s(@DGL,tspan_2,ICs,options,par);

% Concentration profile over time
c_4 = zeros(size_vec_2,1);
c_5 = zeros(size_vec_3,1);
c_6 = zeros(size_vec_3,1);

[~,LocAllB4] = ismembertol(tspan_2',t_all_2(1:end,1),0.0001);
[~,LocAllB5] = ismembertol(tspan_2',t_all_3(1:end,1),0.0001);
[~,LocAllB6] = ismembertol(tspan_2',t_all_3(1:end,1),0.0001);

I2 = zeros(length(t_all_2(1:end,1)),3);
I3 = zeros(length(t_all_3(1:end,1)),3);


for timing = 1:size(t_all_2(1:end,1),1)

 I2(timing,1) = find(timing==LocAllB4);
end

for j = 1:size(t_all_2,1)
 c_4(j,:) = c(I2(j,1),4);
end

for timing = 1:size(t_all_3(1:end,1),1)

 I3(timing,1) = find(timing==LocAllB5);
 I3(timing,2) = find(timing==LocAllB6);
end

for j = 1:size(t_all_3,1)
 c_5(j,:) = c(I3(j,1),5);
 c_6(j,:) = c(I3(j,2),6);
end

 J_final = sum(weight(1)*sum(abs(c_1(:,1)- X(:,1)).^2)...
 +weight(2)*sum(abs(c_2(:,1)- A(:,1)).^2)...
 +weight(3)*sum(abs(c_3(:,1)- B(:,1)).^2)...
 +weight(4)*sum(abs(c_4(:,1)- cag_cloac_CO2_calc_new(:,1)).^2)...
 +weight(5)*sum(abs(c_5(:,1)- cag_cloac_O2_calc_new(:,1)).^2)...
 +weight(6)*sum(abs(c_6(:,1)- cag_cloac_H2_calc_new(:,1)).^2));
end

## ODEs for Batch reactor

function dcdt= DGL(t,c,par)

%Gas constants and calculations
T = 303.15; %Temperature [K]
R = 8314.47; %Gas constant[Pa L moL-1 K-1]
P =101325; %Pressure [Pa]
T_regular = 298.15; % K

Hatm_CO2 = 3.4e-2; %Henry constant for CO2 [mol/L.atm]
Hatm_O2 = 1.3e-3; %Henry constant for O2 [mol/L.atm]
Hatm_H2 = 7.8e-4; %Henry constant for H2 [mol/L.atm]

c_co2 = 2400; %constant for temperature dependent henry constant
c_o2 = 1700; %constant for temperature dependent henry constant
c_h2 = 500; %constant for temperature dependent henry constant

Hatm_CO2_op = Hatm_CO2*exp(c_co2*(1/T-1/T_regular)); % henry constant at 30°C
Hatm_O2_op = Hatm_O2*exp(c_o2*(1/T-1/T_regular)); % henry constant at 30°C
Hatm_H2_op = Hatm_H2*exp(c_h2*(1/T-1/T_regular)); % henry constant at 30°C

He_CO2 = Hatm_CO2_op/P; %Henry constant for CO2 [mol/L.Pa]
He_O2 = Hatm_O2_op/P; %Henry constant for O2 [mol/L.Pa]
He_H2 = Hatm_H2_op/P; %Henry constant for H2 [mol/L.Pa]

% constant parameters
VL =0.05; %liquid volume [L]
VG = 0.95; % Gas volume [L]

%Renaming variables for easier overview
X=c(1); % g/l
PrA=c(2); % g/l
PrB=c(3); % g/l
YCO2= c(4); % %
YO2 = c(5); % %
YH2 = c(6); % %
cCO2_L = c(7); % moles/l
cO2_L = c(8); % moles/l
cH2_L = c(9); % moles/l

mumax = par(1);
mudmin = par(2);
mudmax = par(3);
KLa = par(4);
K_O2 = par(5);
K_CO2 = par(6);
K_H2 = par(7);
K_B = par(8);
q_A_d_max = par(9);
Y_AB = par(10);
K1_A = par(11);
Y_BA_r = par(12);
q_B_upmax = par(13);
Y_XA = par(14);
Y_XB = par(15);
Y_XCO2 = par(16);
Y_XO2 = par(17);
Y_XH2 = par(18);
t_lag = par(19);
q_A_d_max_end = par(20);
K1_A_end = par(21);
Y_BA_r_end = par(22);

mu = mumax* (cCO2_L /(K_CO2+cCO2_L))*(cO2_L/(K_O2+cO2_L)) *(cH2_L/(K_H2+cH2_L)) ; %Specific growth rate of biomass (1/h)
mud =mudmin + mudmax *(cCO2_L /(K_CO2+cCO2_L))*(K_O2/(K_O2+cO2_L)) * (K_H2/(K_H2+cH2_L)); %Specific death rate of biomass(1/h)

q_A_P = mu/Y_XA;
q_B = mu/Y_XB;

dcdt(1,1)= (mu-mud)*X ; %dxdt [g biomass/L h]
dcdt(2,1)= (q_A_P) * X ; % dAcetoin/dt [g/L h]
dcdt(3,1)= (q_B)* X ; % dButandiol/dt [g/L h]
dcdt(4,1)= - KLa * VL/VG * R*T/P * (YCO2*He_CO2*P - cCO2_L); % dCO2/dt in gas [%CO2/h]
dcdt(5,1)= - KLa * VL/VG * R*T/P * (YO2*He_O2*P - cO2_L); % dO2/dt in gas [%O2/h]
dcdt(6,1)= - KLa * VL/VG * R*T/P * (YH2*He_H2*P - cH2_L); % dH2/dt in gas [%H2/h]
dcdt(7,1)= KLa*(YCO2*He_CO2*P - cCO2_L)- X*mu/Y_XCO2; % dCO2/dt in liquid [mole/L h]
dcdt(8,1)= KLa*(YO2*He_O2*P - cO2_L)- X*mu/Y_XO2; % dO2/dt in liquid [mole/L h]
dcdt(9,1)= KLa*(YH2*He_H2*P - cH2_L)- X*mu/Y_XH2 ; % dH2/dt in liquid [mole/L h]

if t>=22 && t<=42
mu = mumax* (cCO2_L /(K_CO2+cCO2_L))*(cO2_L/(K_O2+cO2_L)) *(cH2_L/(K_H2+cH2_L)) ; %Specific growth rate of biomass (1/h)

mu1= mumax*(cO2_L/(K_O2+cO2_L)) *(cH2_L/(K_H2+cH2_L)) ; %Specific growth rate of biomass (1/h)
mud1 =mudmin + mudmax *(K_O2/(K_O2+cO2_L)) * (K_H2/(K_H2+cH2_L)); %Specific death rate of biomass(1/h)

q_A_P = mu/Y_XA;
q_B = mu/Y_XB;

q_A_d = q_A_d_max * PrA/(PrA + K1_A) ;
q_A_d_r = q_A_d * Y_BA_r;

dcdt(1,1)= (mu1-mud1)*X ; %dxdt [g biomass/L h]
dcdt(2,1)= (q_A_P-q_A_d) * X ; % dAcetoin/dt [g/L h]
dcdt(3,1)= (q_B+q_A_d_r)* X ; % dButandiol/dt [g/L h
dcdt(4,1)= - KLa * VL/VG * R*T/P * (YCO2*He_CO2*P - cCO2_L); % dCO2/dt in gas [%CO2/h]
dcdt(5,1)= - KLa * VL/VG * R*T/P * (YO2*He_O2*P - cO2_L); % dO2/dt in gas [%O2/h]
dcdt(6,1)= - KLa * VL/VG * R*T/P * (YH2*He_H2*P - cH2_L); % dH2/dt in gas [%H2/h]
dcdt(7,1)= KLa*(YCO2*He_CO2*P - cCO2_L)- X*mu/Y_XCO2; % dCO2/dt in liquid [mole/L h]
dcdt(8,1)= KLa*(YO2*He_O2*P - cO2_L)- X*mu1/Y_XO2; % dO2/dt in liquid [mole/L h]
dcdt(9,1)= KLa*(YH2*He_H2*P - cH2_L)- X*mu1/Y_XH2 ; % dH2/dt in liquid [mole/L h]

elseif t>42 && t<=71
mu = mumax* (cCO2_L /(K_CO2+cCO2_L))*(cO2_L/(K_O2+cO2_L)) *(cH2_L/(K_H2+cH2_L)) ; %Specific growth rate of biomass (1/h)

mu1= mumax*(cO2_L/(K_O2+cO2_L)) *(cH2_L/(K_H2+cH2_L)) ; %Specific growth rate of biomass (1/h)
mud1 =mudmin + mudmax *(K_O2/(K_O2+cO2_L)) * (K_H2/(K_H2+cH2_L)); %Specific death rate of biomass(1/h)

q_A_P = mu/Y_XA;
q_B = mu/Y_XB;
q_B_r = q_B_upmax*PrB/(PrB+K_B) ;
q_B_rA = q_B_r*Y_AB ;

dcdt(1,1)= (mu1-mud1)*X ; %dxdt [g biomass/L h]
dcdt(2,1)= (q_A_P+q_B_rA) * X ; % dAcetoin/dt [g/L h]
dcdt(3,1)= (q_B - q_B_r)* X ; % dButandiol/dt [g/L h
dcdt(4,1)= - KLa * VL/VG * R*T/P * (YCO2*He_CO2*P - cCO2_L); % dCO2/dt in gas [%CO2/h]
dcdt(5,1)= - KLa * VL/VG * R*T/P * (YO2*He_O2*P - cO2_L); % dO2/dt in gas [%O2/h]
dcdt(6,1)= - KLa * VL/VG * R*T/P * (YH2*He_H2*P - cH2_L); % dH2/dt in gas [%H2/h]
dcdt(7,1)= KLa*(YCO2*He_CO2*P - cCO2_L)- X*mu/Y_XCO2; % dCO2/dt in liquid [mole/L h]
dcdt(8,1)= KLa*(YO2*He_O2*P - cO2_L)- X*mu1/Y_XO2; % dO2/dt in liquid [mole/L h]
dcdt(9,1)= KLa*(YH2*He_H2*P - cH2_L)- X*mu1/Y_XH2 ; % dH2/dt in liquid [mole/L h]

elseif t>71
mu = mumax* (cCO2_L /(K_CO2+cCO2_L))*(cO2_L/(K_O2+cO2_L)) *(cH2_L/(K_H2+cH2_L)) ; %Specific growth rate of biomass (1/h)

mu1= mumax*(cO2_L/(K_O2+cO2_L)) *(cH2_L/(K_H2+cH2_L)) ; %Specific growth rate of biomass (1/h)
mud1 =mudmin + mudmax *(K_O2/(K_O2+cO2_L)) * (K_H2/(K_H2+cH2_L)); %Specific death rate of biomass(1/h)

q_A_P = mu/Y_XA;
q_B = mu/Y_XB;
q_A_d_end = q_A_d_max_end* PrA/(PrA + K1_A_end) ;
q_A_d_r_end= q_A_d_end * Y_BA_r_end;

dcdt(1,1)= (mu1-mud1)*X ; %dxdt [g biomass/L h]
dcdt(2,1)= (q_A_P-q_A_d_end) * X ; % dAcetoin/dt [g/L h]
dcdt(3,1)= (q_B+q_A_d_r_end)* X ; % dButandiol/dt [g/L h
dcdt(4,1)= - KLa * VL/VG * R*T/P * (YCO2*He_CO2*P - cCO2_L); % dCO2/dt in gas [%CO2/h]
dcdt(5,1)= - KLa * VL/VG * R*T/P * (YO2*He_O2*P - cO2_L); % dO2/dt in gas [%O2/h]
dcdt(6,1)= - KLa * VL/VG * R*T/P * (YH2*He_H2*P - cH2_L); % dH2/dt in gas [%H2/h]
dcdt(7,1)= KLa*(YCO2*He_CO2*P - cCO2_L)- X*mu/Y_XCO2; % dCO2/dt in liquid [mole/L h]
dcdt(8,1)= KLa*(YO2*He_O2*P - cO2_L)- X*mu1/Y_XO2; % dO2/dt in liquid [mole/L h]
dcdt(9,1)= KLa*(YH2*He_H2*P - cH2_L)- X*mu1/Y_XH2 ; % dH2/dt in liquid [mole/L h]
end
end

clc
clear all
close all

 % Data imported from Excel
budC_pneum_Time_1 =xlsread('Autotroph.xlsx','budC(pneum)','E2:E10'); %h
budC_pneum_OD=xlsread('Autotroph.xlsx','budC(pneum)','I2:I10'); %OD600
budC_pneum_X = (0.6417 * budC_pneum_OD + 0.0632); %g/l
budC_pneum_Acetoin=xlsread('Autotroph.xlsx','budC(pneum)','AM2:AM10'); %g/l
budC_pneum_Butandiol=xlsread('Autotroph.xlsx','budC(pneum)','AN2:AN10'); %g/l
budC_pneum_Time_2=xlsread('Autotroph.xlsx','budC(pneum)','B34:B38'); %h
budC_pneum_CO2=xlsread('Autotroph.xlsx','budC(pneum)','C34:C38'); %
budC_pneum_CO2_calc = budC_pneum_CO2/100;
budC_pneum_O2=xlsread('Autotroph.xlsx','budC(pneum)','G34:G38'); %
budC_pneum_O2_calc = budC_pneum_O2/100;
budC_pneum_H2=xlsread('Autotroph.xlsx','budC(pneum)','E34:E38'); %
budC_pneum_H2_calc = budC_pneum_H2/100;

budC_pneum_Time_3=xlsread('Autotroph.xlsx','budC(pneum)','AS2:AS11'); %h
budC_pneum_Time_4=xlsread('Autotroph.xlsx','budC(pneum)','AU2:AU13'); %h

budC_pneum_CO2_new=xlsread('Autotroph.xlsx','budC(pneum)','AR2:AR11'); %
budC_pneum_CO2_calc_new = budC_pneum_CO2_new/100;
budC_pneum_O2_new=xlsread('Autotroph.xlsx','budC(pneum)','AV2:AV13'); %
budC_pneum_O2_calc_new = budC_pneum_O2_new/100;
budC_pneum_H2_new=xlsread('Autotroph.xlsx','budC(pneum)','AT2:AT13'); %
budC_pneum_H2_calc_new = budC_pneum_H2_new/100;

T1 = budC_pneum_Time_1;
OD = budC_pneum_OD;
X = budC_pneum_X;
A = budC_pneum_Acetoin;
B = budC_pneum_Butandiol;
T2 = budC_pneum_Time_2;
CO2_calc = budC_pneum_CO2_calc;
O2_calc = budC_pneum_O2_calc;
H2_calc = budC_pneum_H2_calc;

%Parameters chosen by hand for curve fitting and optimization
mumax = 0.02; %1 % Maximum specific growth rate(1/h)
mudmin = 0.001; %2 %Minimum specific death rate (1/h)
mudmax = 0.02; %3 %Maximum specific death rate (1/h)
KLa = 150; %4 %Mass transfer coefficient for shaking flasks (1/h)
K_O2 = 0.00003125; %5 % (Monod constant) Half-saturation constant for O2 (in g/L) mole/L
K_CO2 = 0.0000227; %6 % (Monod constant) Half-saturation constant for CO2 (in g/L) mole/L
K_H2 = 0.0005; %7 % (Monod constant) Half-saturation constant for H2 (in g/L) mole/L
K_B = 0.005; %8 (Monod constant) Half-saturation constant butandiol (g/l)
q_A_d_max = 0.00001; %9 Maximum specific consumption rate of Acetoin by biomass after the depletion of CO2 (g acetoin/g biomass)
Y_AB = 0.97; %10 Yield coefficient of Acetoin converted back from Butandiol (g acetoin/g Butanediol)
K1_A = 0.005; %11 (Monod constant) Half-saturation constant acetoin during first forward reaction after co2 depletion (g/l)
Y_BA_r = 1.02; %12 Yield coefficient of Butandiol converted from Acetoin (g butandiol/g acetoin)
q_B_upmax = 0.00001; %13 Maximum specific consumption rate of butandiol by biomass after the depletion of CO2 with still oxygen (g butandiol / g biomass)
Y_XA = 0.005; %14 Yield coefficient of biomass produced acetoin (g biomass/g acetoin)
Y_XB = 0.005; %15 Yield coefficient of biomass produced butandiol (g biomass/g butandiol)
Y_XCO2 = 0.005; %16 Yield coefficient of biomass per CO2 (g biomass/mole CO2)
Y_XO2 = 0.005; %17 Yield coefficient of biomass per O2 (g biomass/mole O2)
Y_XH2 = 0.005; %18 Yield coefficient of biomass per H2 (g biomass/mole H2)
t_lag = 15; %19 time term for lag phase and delayed grwoth or production (h)
q_A_d_max_end = 0.00001; %20 Maximum specific consumption rate of acetoin by biomass after the depletion of CO2 and O2 (g acetoin / g biomass)
K2_A = 0.005; %21 (Monod constant) Half-saturation constant acetoin after co2 and o2 depletion (g/l)
Y_BA_r_end = 1.02; %22 Yield coefficient of Butandiol converted from Acetoin after co2 and o2 depletion (g butandiol/g acetoin)

%molare Massen
Acetoin = 88.11; %g/mol
zweidreiButandiol = 90.14; %g/mol
CO2 = 44.01; %g/mol
H2 = 2;%g/mol
O2 = 32;%g/mol

%Gas constants and calculations
T = 303.15; %Temperature [K]
R = 8314.47; %Gas constant[Pa L moL-1 K-1]
P =101325; %Pressure [Pa]
T_regular = 298.15; % K

Hatm_CO2 = 3.4e-2; %Henry constant for CO2 [mol/L.atm]
Hatm_O2 = 1.3e-3; %Henry constant for O2 [mol/L.atm]
Hatm_H2 = 7.8e-4; %Henry constant for H2 [mol/L.atm]

c_co2 = 2400; %constant for temperature dependent henry constant
c_o2 = 1700; %constant for temperature dependent henry constant
c_h2 = 500; %constant for temperature dependent henry constant

Hatm_CO2_op = Hatm_CO2*exp(c_co2*(1/T-1/T_regular)); % henry constant at 30°C
Hatm_O2_op = Hatm_O2*exp(c_o2*(1/T-1/T_regular)); % henry constant at 30°C
Hatm_H2_op = Hatm_H2*exp(c_h2*(1/T-1/T_regular)); % henry constant at 30°C

He_CO2 = Hatm_CO2_op/P; %Henry constant for CO2 [mol/L.Pa]
He_O2 = Hatm_O2_op/P; %Henry constant for O2 [mol/L.Pa]
He_H2 = Hatm_H2_op/P; %Henry constant for H2 [mol/L.Pa]
%Gases initial (percentage) in air
YO2_0=O2_calc(1,1); %initial proportion oxygen in gas (%)
YCO2_0=CO2_calc(1,1); %initial proportion CO2 in gas (%)
YH2_0=H2_calc(1,1); %initial proportion H2 in gas (%)

%Initial Gases concentrations in liquid (mol/L)
 cCO2_L_0 = YCO2_0*P*He_CO2;
 cO2_L_0 = YO2_0*P*He_O2;
 cH2_L_0 = YH2_0*P*He_H2;

% Defines intitial values from data and guessed parameters
ICs = [X(1,1), A(1,1), B(1,1), CO2_calc(1,1),O2_calc(1,1),H2_calc(1,1), cCO2_L_0,cO2_L_0, cH2_L_0]; %Initial conditions

par0 = [mumax, mudmin, mudmax, KLa,K_O2, K_CO2, K_H2, K_B,q_A_d_max,Y_AB,K1_A,Y_BA_r,q_B_upmax,Y_XA,Y_XB,Y_XCO2,Y_XO2,Y_XH2, t_lag,q_A_d_max_end,K2_A,Y_BA_r_end];

% Lower bounds
lowerBounds = [0.001, 0.0001, 0.001, 50, 0, 0.00001, 0.00001, 0, 0, 0.8, 0, 0.8, 0, 0, 0, 0, 0, 0, 0 ,0, 0, 0.8 ];
% Upper bound
upperBounds = [0.2, 0.01, 0.2, 200, 2, 2, 2, 8, 10, 1.2, 8, 1.2, 2, 40, 40, 10, 10, 10, 200, 10, 8, 1.2];

## Use fmincon

options = optimset('Display', 'Iter', 'MaxIter', 200, 'TolFun', 1e-6, 'TolX', 1e-6);

paropt = fmincon(@objective, par0, [], [], [], [], lowerBounds, upperBounds, [], options, ICs, T1, X, A, B, T2, CO2_calc, O2_calc, H2_calc,budC_pneum_Time_3,budC_pneum_Time_4,budC_pneum_CO2_calc_new,budC_pneum_O2_calc_new,budC_pneum_H2_calc_new);
Results_par = paropt; % Safe estimated parameters

## Final simulation

tspan1 = T1(:,1);
ttspan1 = 0:0.5:max(tspan1 + 4);
tspan2 = T2(:,1);
ttspan2 = 0:0.5:max(tspan2 + 4);

options=odeset('reltol',1E-4,'abstol',1E-4);
[t,c] = ode15s(@DGL,ttspan2,ICs, options, Results_par); %

OD_graph = (c(:,1) - 0.0632)/0.6417;

figure('Name','budC(pneum)','NumberTitle','off');
subplot(3,1,1)
plot(tspan1, OD(:,1), 'ob');
hold on
plot(t, OD_graph , '-r', 'LineWidth', 1);
hold off
x=xlabel('Time [h]');
y=ylabel('OD_{600}');
legend('Lab','Model');
x.Color='black';
y.Color='black';
ax = gca;
ax.YColor = 'k';

subplot(3,1,2)
plot(t,c(:,2),'-r',tspan1,A(:,1),'or');
x1=xlabel('Time [h]');
y1=ylabel('Acetoin [g L^-^1]');
legend('Acetoin', 'exp. Data Acetoin','Location','southeast','NumColumns',2);
y1.Color='black';
x1.Color='black';
ax = gca;
ax.YColor = 'k';
hold off

subplot(3,1,3)
plot(t,c(:,3),'-b',tspan1,B(:,1),'ob');
x1=xlabel('Time [h]');
y1=ylabel('Butandiol [g L^-^1]');
legend('Butandiol','exp. Data Butandiol','Location','southeast','NumColumns',2);
x1.Color='black';
y1.Color='black';
ax = gca;
ax.YColor = 'k';
hold off

figure('Name','budC(pneum)','NumberTitle','off');
subplot (3,1,1)
plot(t,c(:,4).*100,'-b',tspan2,CO2_calc(:,1).*100,'og');
legend('CO2','exp. Data CO2','Location','southeast','NumColumns',2);
y1=ylabel('CO2 [%]');
x1=xlabel('Time [h]');
x1.Color='black';
y1.Color='black';
ax = gca;
ax.YColor = 'k';
hold off

subplot (3,1,2)
plot(t,c(:,5).*100,'-b',tspan2,O2_calc(:,1).*100,'og');
legend('O2','exp. Data CO2','Location','southeast','NumColumns',2);
y1=ylabel('O2 [%]');
x1=xlabel('Time [h]');
x1.Color='black';
y1.Color='black';
ax = gca;
ax.YColor = 'k';
hold off

subplot (3,1,3)
plot(t,c(:,6).*100,'-b',tspan2,H2_calc(:,1).*100,'og');
legend('H2','exp. Data CO2','Location','southeast','NumColumns',2);
y1=ylabel('H2 [%]');
x1=xlabel('Time [h]');
x1.Color='black';
y1.Color='black';
ax = gca;
ax.YColor = 'k';
hold off

figure('Name','budC(pneum)','NumberTitle','off');
subplot (3,1,1)
plot(t,c(:,7),'-b');
legend('CO2','Location','southeast','NumColumns',2);
y1=ylabel('CO2 [mole L^-^1]');
x1=xlabel('Time [h]');
x1.Color='black';
y1.Color='black';
ax = gca;
ax.YColor = 'k';
hold off

subplot (3,1,2)
plot(t,c(:,8),'-b');
legend('O2 in liquid','Location','southeast','NumColumns',2);
y1=ylabel('O2 in liquid [mole L^-^1]');
x1=xlabel('Time [h]');
x1.Color='black';
y1.Color='black';
ax = gca;
ax.YColor = 'k';
hold off

subplot (3,1,3)
legend('H2 in liquid','Location','southeast','NumColumns',2);
plot(t,c(:,9),'-b');
y1=ylabel('H2 [mole L^-^1]');
x1=xlabel('Time [h]');
x1.Color='black';
y1.Color='black';
ax = gca;
ax.YColor = 'k';
hold off

%
TT1 = 2 * (T1 + 1);
TT1 = round(TT1);

TT2 = 2 * (T2 +1) ;
TT2 = round(TT2);

lab1 = OD;
lab2 = A;
lab3 = B;
lab4 = CO2_calc;
lab5 = O2_calc;
lab6 = H2_calc;

model1 = OD_graph(TT1 ,1);
model2 = c(TT1,2);
model3 = c(TT1,3);
model4 = c(TT2,4);
model5 = c(TT2,5);
model6 = c(TT2,6);

% Calculate the squared error
SQ1 = sum((lab1 - model1).^2);
SQ2 = sum((lab2 - model2).^2);
SQ3 = sum((lab3 - model3).^2);
SQ4 = sum((lab4 - model4).^2);
SQ5 = sum((lab5 - model5).^2);
SQ6 = sum((lab6 - model6).^2);

%
MSQ1 = sum((lab1 - mean(lab1)).^2);
MSQ2 = sum((lab2 - mean(lab2)).^2);
MSQ3 = sum((lab3 - mean(lab3)).^2);
MSQ4 = sum((lab4 - mean(lab4)).^2);
MSQ5 = sum((lab5 - mean(lab5)).^2);
MSQ6 = sum((lab6 - mean(lab6)).^2);

%
rmse1 = 1 - SQ1/MSQ1;
rmse2 = 1 - SQ2/MSQ2;
rmse3 = 1 - SQ3/MSQ3;
rmse4 = 1 - SQ4/MSQ4;
rmse5 = 1 - SQ5/MSQ5;
rmse6 = 1 - SQ6/MSQ6;

RMSE = [rmse1 rmse2 rmse3 rmse4 rmse5 rmse6 ];
% Display the RMSE
disp('RMSE: ');
disp(RMSE);

%molare Massen
Acetoin = 88.11; %g/mol
zweidreiButandiol = 90.14; %g/mol

out_file = 'Autotrophfit';
xlswrite(out_file,OD_graph(:,1),'Fit1','X8');%OD
xlswrite(out_file,c(:,2)./Acetoin.*1000,'Fit1','Y8'); % acetoin mM
xlswrite(out_file,c(:,3)./zweidreiButandiol.*1000,'Fit1','Z8'); % butandiol mM
xlswrite(out_file,c(:,4).*100,'Fit1','AA8'); %CO2gas
xlswrite(out_file,c(:,5).*100,'Fit1','AB8');%O2gas
xlswrite(out_file,c(:,6).*100,'Fit1','AC8');%H2gas
xlswrite(out_file,c(:,7),'Fit1','AD8');%CO2 liquid
xlswrite(out_file,c(:,8),'Fit1','AE8');%O2 liquid
xlswrite(out_file,c(:,9),'Fit1','AF8');%H2 liquid
xlswrite(out_file,t(:,1),'Fit1','W8');%Time

xlswrite(out_file,paropt(:,:),'Fit1','H3'); %
xlswrite(out_file,rmse1,'Fit1','A3'); %
xlswrite(out_file,rmse2,'Fit1','B3'); %
xlswrite(out_file,rmse3,'Fit1','C3'); %
xlswrite(out_file,rmse4,'Fit1','D3'); %
xlswrite(out_file,rmse5,'Fit1','E3'); %
xlswrite(out_file,rmse6,'Fit1','F3'); %

## Objective

function J_final=objective(par,ICs,T1, X, A, B, T2,CO2_calc, O2_calc, H2_calc,budC_pneum_Time_3,budC_pneum_Time_4,budC_pneum_CO2_calc_new,budC_pneum_O2_calc_new,budC_pneum_H2_calc_new)

weight = [1;...% weighting viable cell densitiy
 8;... % weighting Acetoin
 8;... % weighting Butandiol
 450;... % weighting CO2
 120;... % Weighting O2
 12];... % Weighting H2

% Solves the DGL System with starting values
options=odeset('reltol',1E-09,'abstol',1E-09,'maxstep',0.1,'normcontrol','on');

t_all = T1;

tspan = 0:0.1:max(T1)+1;

size_vec = size(T1,1);

[~,c] = ode15s(@DGL,tspan,ICs,options,par);

% Concentration profile over time
c_1 = zeros(size_vec,1);
c_2 = zeros(size_vec,1);
c_3 = zeros(size_vec,1);

[~,LocAllB] = ismembertol(tspan',t_all(1:end,1),0.0001);
[~,LocAllB2] = ismembertol(tspan',t_all(1:end,1),0.0001);
[~,LocAllB3] = ismembertol(tspan',t_all(1:end,1),0.0001);

I = zeros(length(t_all(1:end,1)),3);

for timing = 1:size(t_all(1:end,1),1)

 I(timing,1) = find(timing==LocAllB);
 I(timing,2) = find(timing==LocAllB2);
 I(timing,3) = find(timing==LocAllB3);
end


for j = 1:size(t_all,1)
 c_1(j,:) = c(I(j,1),1);
 c_2(j,:) = c(I(j,2),2);
 c_3(j,:) = c(I(j,3),3);
end

% Solves the DGL System with starting values
options=odeset('reltol',1E-09,'abstol',1E-09,'maxstep',0.1,'normcontrol','on');

t_all_2 = round(budC_pneum_Time_3,1);
t_all_3 = round(budC_pneum_Time_4,1);


tspan_2 = 0:0.1:max(budC_pneum_Time_3)+1;

size_vec_2 = size(budC_pneum_Time_3,1);
size_vec_3 = size(budC_pneum_Time_4,1);

[~,c] = ode15s(@DGL,tspan_2,ICs,options,par);

% Concentration profile over time
c_4 = zeros(size_vec_2,1);
c_5 = zeros(size_vec_3,1);
c_6 = zeros(size_vec_3,1);

[~,LocAllB4] = ismembertol(tspan_2',t_all_2(1:end,1),0.0001);
[~,LocAllB5] = ismembertol(tspan_2',t_all_3(1:end,1),0.0001);
[~,LocAllB6] = ismembertol(tspan_2',t_all_3(1:end,1),0.0001);

I2 = zeros(length(t_all_2(1:end,1)),3);
I3 = zeros(length(t_all_3(1:end,1)),3);

for timing = 1:size(t_all_2(1:end,1),1)

 I2(timing,1) = find(timing==LocAllB4);

end

for j = 1:size(t_all_2,1)
 c_4(j,:) = c(I2(j,1),4);

end

for timing = 1:size(t_all_3(1:end,1),1)

 I3(timing,1) = find(timing==LocAllB5);
 I3(timing,2) = find(timing==LocAllB6);
end

for j = 1:size(t_all_3,1)
 c_5(j,:) = c(I3(j,1),5);
 c_6(j,:) = c(I3(j,2),6);
end

 J_final = sum(weight(1)*sum(abs(c_1(:,1)- X(:,1)).^2)...
 +weight(2)*sum(abs(c_2(:,1)- A(:,1)).^2)...
 +weight(3)*sum(abs(c_3(:,1)- B(:,1)).^2)...
 +weight(4)*sum(abs(c_4(:,1)- budC_pneum_CO2_calc_new(:,1)).^2)...
 +weight(5)*sum(abs(c_5(:,1)- budC_pneum_O2_calc_new(:,1)).^2)...
 +weight(6)*sum(abs(c_6(:,1)- budC_pneum_H2_calc_new(:,1)).^2));

end

## ODEs for Batch reactor

function dcdt= DGL(t,c,par)

%Gas constants and calculations
T = 303.15; %Temperature [K]
R = 8314.47; %Gas constant[Pa L moL-1 K-1]
P =101325; %Pressure [Pa]
T_regular = 298.15; % K

Hatm_CO2 = 3.4e-2; %Henry constant for CO2 [mol/L.atm]
Hatm_O2 = 1.3e-3; %Henry constant for O2 [mol/L.atm]
Hatm_H2 = 7.8e-4; %Henry constant for H2 [mol/L.atm]

c_co2 = 2400; %constant for temperature dependent henry constant
c_o2 = 1700; %constant for temperature dependent henry constant
c_h2 = 500; %constant for temperature dependent henry constant

Hatm_CO2_op = Hatm_CO2*exp(c_co2*(1/T-1/T_regular)); % henry constant at 30°C
Hatm_O2_op = Hatm_O2*exp(c_o2*(1/T-1/T_regular)); % henry constant at 30°C
Hatm_H2_op = Hatm_H2*exp(c_h2*(1/T-1/T_regular)); % henry constant at 30°C

He_CO2 = Hatm_CO2_op/P; %Henry constant for CO2 [mol/L.Pa]
He_O2 = Hatm_O2_op/P; %Henry constant for O2 [mol/L.Pa]
He_H2 = Hatm_H2_op/P; %Henry constant for H2 [mol/L.Pa]

% constant parameters
VL =0.05; %liquid volume [L]
VG = 0.95; % Gas volume [L]

%Renaming variables for easier overview
X=c(1); % g/l
PrA=c(2); % g/l
PrB=c(3); % g/l
YCO2= c(4); % %
YO2 = c(5); % %
YH2 = c(6); % %
cCO2_L = c(7); % moles/l
cO2_L = c(8); % moles/l
cH2_L = c(9); % moles/l

%Parameters after optimization

mumax = par(1);
mudmin = par(2);
mudmax = par(3);
KLa = par(4);
K_O2 = par(5);
K_CO2 = par(6);
K_H2 = par(7);
K_B = par(8);
q_A_d_max = par(9);
Y_AB = par(10);
K1_A = par(11);
Y_BA_r = par(12);
q_B_upmax = par(13);
Y_XA = par(14);
Y_XB = par(15);
Y_XCO2 = par(16);
Y_XO2 = par(17);
Y_XH2 = par(18);
t_lag = par(19);
q_A_d_max_end = par(20);
K2_A = par(21);
Y_BA_r_end = par(22);

mu = mumax* (cCO2_L /(K_CO2+cCO2_L))*(cO2_L/(K_O2+cO2_L)) *(cH2_L/(K_H2+cH2_L)) ; %Specific growth rate of biomass (1/h)
mud =mudmin + mudmax *(cCO2_L /(K_CO2+cCO2_L))*(K_O2/(K_O2+cO2_L)) * (K_H2/(K_H2+cH2_L)); %Specific death rate of biomass(1/h)

q_A_P = mu/Y_XA;
q_B = mu/Y_XB*(1-exp(-t/t_lag));

dcdt(1,1)= (mu-mud)*X ; %dxdt [g biomass/L h]
dcdt(2,1)= (q_A_P) * X ; % dAcetoin/dt [g/L h]
dcdt(3,1)= (q_B)* X ; % dButandiol/dt [g/L h]
dcdt(4,1)= - KLa * VL/VG * R*T/P * (YCO2*He_CO2*P - cCO2_L); % dCO2/dt in gas [%CO2/h]
dcdt(5,1)= - KLa * VL/VG * R*T/P * (YO2*He_O2*P - cO2_L); % dO2/dt in gas [%O2/h]
dcdt(6,1)= - KLa * VL/VG * R*T/P * (YH2*He_H2*P - cH2_L); % dH2/dt in gas [%H2/h]
dcdt(7,1)= KLa*(YCO2*He_CO2*P - cCO2_L)- X*mu/Y_XCO2; % dCO2/dt in liquid [mole/L h]
dcdt(8,1)= KLa*(YO2*He_O2*P - cO2_L)- X*mu/Y_XO2; % dO2/dt in liquid [mole/L h]
dcdt(9,1)= KLa*(YH2*He_H2*P - cH2_L)- X*mu/Y_XH2 ; % dH2/dt in liquid [mole/L h]

if t>=55 && t<=70

mu = mumax* (cCO2_L /(K_CO2+cCO2_L))*(cO2_L/(K_O2+cO2_L)) *(cH2_L/(K_H2+cH2_L)) ; %Specific growth rate of biomass (1/h)

mu1= mumax*(cO2_L/(K_O2+cO2_L)) *(cH2_L/(K_H2+cH2_L)) ; %Specific growth rate of biomass (1/h)
mud1 =mudmin + mudmax *(K_O2/(K_O2+cO2_L)) * (K_H2/(K_H2+cH2_L)); %Specific death rate of biomass(1/h)

q_A_P = mu/Y_XA;
q_B = mu/Y_XB*(1-exp(-t/t_lag));
q_A_d = q_A_d_max * PrA/(PrA + K1_A) ;
q_A_d_r = q_A_d * Y_BA_r;

dcdt(1,1)= (mu1-mud1)*X ; %dxdt [g biomass/L h]
dcdt(2,1)= (q_A_P-q_A_d) * X ; % dAcetoin/dt [g/L h]
dcdt(3,1)= (q_B+q_A_d_r)* X ; % dButandiol/dt [g/L h
dcdt(4,1)= - KLa * VL/VG * R*T/P * (YCO2*He_CO2*P - cCO2_L); % dCO2/dt in gas [%CO2/h]
dcdt(5,1)= - KLa * VL/VG * R*T/P * (YO2*He_O2*P - cO2_L); % dO2/dt in gas [%O2/h]
dcdt(6,1)= - KLa * VL/VG * R*T/P * (YH2*He_H2*P - cH2_L); % dH2/dt in gas [%H2/h]
dcdt(7,1)= KLa*(YCO2*He_CO2*P - cCO2_L)- X*mu/Y_XCO2; % dCO2/dt in liquid [mole/L h]
dcdt(8,1)= KLa*(YO2*He_O2*P - cO2_L)- X*mu1/Y_XO2; % dO2/dt in liquid [mole/L h]
dcdt(9,1)= KLa*(YH2*He_H2*P - cH2_L)- X*mu1/Y_XH2 ; % dH2/dt in liquid [mole/L h]

elseif t>70 && t<=100
mu = mumax* (cCO2_L /(K_CO2+cCO2_L))*(cO2_L/(K_O2+cO2_L)) *(cH2_L/(K_H2+cH2_L)) ; %Specific growth rate of biomass (1/h)

mu1= mumax*(cO2_L/(K_O2+cO2_L)) *(cH2_L/(K_H2+cH2_L)) ; %Specific growth rate of biomass (1/h)
mud1 =mudmin + mudmax *(K_O2/(K_O2+cO2_L)) * (K_H2/(K_H2+cH2_L)); %Specific death rate of biomass(1/h)

q_A_P = mu/Y_XA;
q_B = mu/Y_XB*(1-exp(-t/t_lag));
q_B_r = q_B_upmax*PrB/(PrB+K_B) ;
q_B_rA = q_B_r*Y_AB ;

dcdt(1,1)= (mu1-mud1)*X ; %dxdt [g biomass/L h]
dcdt(2,1)= (q_A_P+q_B_rA) * X ; % dAcetoin/dt [g/L h]
dcdt(3,1)= (q_B-q_B_r)* X ; % dButandiol/dt [g/L h
dcdt(4,1)= - KLa * VL/VG * R*T/P * (YCO2*He_CO2*P - cCO2_L); % dCO2/dt in gas [%CO2/h]
dcdt(5,1)= - KLa * VL/VG * R*T/P * (YO2*He_O2*P - cO2_L); % dO2/dt in gas [%O2/h]
dcdt(6,1)= - KLa * VL/VG * R*T/P * (YH2*He_H2*P - cH2_L); % dH2/dt in gas [%H2/h]
dcdt(7,1)= KLa*(YCO2*He_CO2*P - cCO2_L)- X*mu/Y_XCO2; % dCO2/dt in liquid [mole/L h]
dcdt(8,1)= KLa*(YO2*He_O2*P - cO2_L)- X*mu1/Y_XO2; % dO2/dt in liquid [mole/L h]
dcdt(9,1)= KLa*(YH2*He_H2*P - cH2_L)- X*mu1/Y_XH2 ; % dH2/dt in liquid [mole/L h]

elseif t>100
mu = mumax* (cCO2_L /(K_CO2+cCO2_L))*(cO2_L/(K_O2+cO2_L)) *(cH2_L/(K_H2+cH2_L)) ; %Specific growth rate of biomass (1/h)

mu1= mumax*(cO2_L/(K_O2+cO2_L)) *(cH2_L/(K_H2+cH2_L)) ; %Specific growth rate of biomass (1/h)
mud1 =mudmin + mudmax *(K_O2/(K_O2+cO2_L)) * (K_H2/(K_H2+cH2_L)); %Specific death rate of biomass(1/h)

q_A_P = mu/Y_XA;
q_B = mu/Y_XB*(1-exp(-t/t_lag));
q_A_d_end = q_A_d_max_end * PrA/(PrA + K2_A) ;
q_A_d_r_end = q_A_d_end * Y_BA_r_end;

dcdt(1,1)= (mu1-mud1)*X ; %dxdt [g biomass/L h]
dcdt(2,1)= (q_A_P-q_A_d_end) * X ; % dAcetoin/dt [g/L h]
dcdt(3,1)= (q_B+q_A_d_r_end)* X ; % dButandiol/dt [g/L h
dcdt(4,1)= - KLa * VL/VG * R*T/P * (YCO2*He_CO2*P - cCO2_L); % dCO2/dt in gas [%CO2/h]
dcdt(5,1)= - KLa * VL/VG * R*T/P * (YO2*He_O2*P - cO2_L); % dO2/dt in gas [%O2/h]
dcdt(6,1)= - KLa * VL/VG * R*T/P * (YH2*He_H2*P - cH2_L); % dH2/dt in gas [%H2/h]
dcdt(7,1)= KLa*(YCO2*He_CO2*P - cCO2_L)- X*mu/Y_XCO2; % dCO2/dt in liquid [mole/L h]
dcdt(8,1)= KLa*(YO2*He_O2*P - cO2_L)- X*mu1/Y_XO2; % dO2/dt in liquid [mole/L h]
dcdt(9,1)= KLa*(YH2*He_H2*P - cH2_L)- X*mu1/Y_XH2 ; % dH2/dt in liquid [mole/L h]
end
end

clear all
close all

 % Data imported from Excel
cag_pneum_Time_1 =xlsread('Autotroph.xlsx','cag(pneum)','E2:E9'); %h
cag_pneum_OD=xlsread('Autotroph.xlsx','cag(pneum)','I2:I9'); %OD600
cag_pneum_X = (0.6417 * cag_pneum_OD + 0.0632); %g/l
cag_pneum_Acetoin=xlsread('Autotroph.xlsx','cag(pneum)','AM2:AM9'); %g/l
cag_pneum_Butandiol=xlsread('Autotroph.xlsx','cag(pneum)','AN2:AN9'); %g/l
cag_pneum_Time_2=xlsread('Autotroph.xlsx','cag(pneum)','B32:B36'); %h
cag_pneum_CO2=xlsread('Autotroph.xlsx','cag(pneum)','C32:C36'); %
cag_pneum_CO2_calc = cag_pneum_CO2/100;
cag_pneum_O2=xlsread('Autotroph.xlsx','cag(pneum)','G32:G36'); %
cag_pneum_O2_calc = cag_pneum_O2/100;
cag_pneum_H2= xlsread('Autotroph.xlsx','cag(pneum)','E32:E36'); %
cag_pneum_H2_calc = cag_pneum_H2/100;

cag_pneum_Time_3=xlsread('Autotroph.xlsx','cag(pneum)','AS2:AS8'); %h
cag_pneum_Time_4=xlsread('Autotroph.xlsx','cag(pneum)','AV2:AV11'); %h

cag_pneum_CO2_new=xlsread('Autotroph.xlsx','cag(pneum)','AR2:AR8'); %
cag_pneum_CO2_calc_new = cag_pneum_CO2_new/100;
cag_pneum_O2_new=xlsread('Autotroph.xlsx','cag(pneum)','AU2:AU11'); %
cag_pneum_O2_calc_new = cag_pneum_O2_new/100;
cag_pneum_H2_new= xlsread('Autotroph.xlsx','cag(pneum)','AX2:AX11'); %
cag_pneum_H2_calc_new = cag_pneum_H2_new/100;

T1 = cag_pneum_Time_1;
OD = cag_pneum_OD;
X = cag_pneum_X;
A = cag_pneum_Acetoin;
B = cag_pneum_Butandiol;
T2 = cag_pneum_Time_2;
CO2_calc = cag_pneum_CO2_calc;
O2_calc = cag_pneum_O2_calc;
H2_calc = cag_pneum_H2_calc;

%Parameters chosen by hand for curve fitting and optimization
mumax = 0.02; %1 % Maximum specific growth rate(1/h)
mudmin = 0.001; %2 %Minimum specific death rate (1/h)
mudmax = 0.02; %3 %Maximum specific death rate (1/h)
KLa = 150; %4 %Mass transfer coefficient for shaking flasks (1/h)
K_O2 = 0.00003125; %5 % (Monod constant) Half-saturation constant for O2 (in g/L) mole/L
K_CO2 = 0.0000227; %6 % (Monod constant) Half-saturation constant for CO2 (in g/L) mole/L
K_H2 = 0.0005; %7 % (Monod constant) Half-saturation constant for H2 (in g/L) mole/L
K_B = 0.005; %8 (Monod constant) Half-saturation constant butandiol (g/l)
q_A_d_max = 0.00001; %9 Maximum specific consumption rate of Acetoin by biomass after the depletion of CO2 (g acetoin/g biomass)
Y_AB = 0.97; %10 Yield coefficient of Acetoin converted back from Butandiol (g acetoin/g Butanediol)
K1_A = 0.005; %11 (Monod constant) Half-saturation constant acetoin during first forward reaction after co2 depletion (g/l)
Y_BA_r = 1.02; %12 Yield coefficient of Butandiol converted from Acetoin (g butandiol/g acetoin)
q_B_upmax = 0.00001; %13 Maximum specific consumption rate of butandiol by biomass after the depletion of CO2 with still oxygen (g butandiol / g biomass)
Y_XA = 0.005; %14 Yield coefficient of biomass produced acetoin (g biomass/g acetoin)
Y_XB = 0.005; %15 Yield coefficient of biomass produced butandiol (g biomass/g butandiol)
Y_XCO2 = 0.005; %16 Yield coefficient of biomass per CO2 (g biomass/mole CO2)
Y_XO2 = 0.005; %17 Yield coefficient of biomass per O2 (g biomass/mole O2)
Y_XH2 = 0.005; %18 Yield coefficient of biomass per H2 (g biomass/mole H2)
t_lag = 2; %19 time term for lag phase and delayed grwoth or production (h)
q_A_d_max_end = 0.0001; %20 Maximum specific consumption rate of acetoin by biomass after the depletion of CO2 and O2 (g acetoin / g biomass)
K1_A_end = 0.005; % 21 (Monod constant) Half-saturation constant acetoin after co2 and o2 depletion (g/l)
Y_BA_r_end = 1.02; %22 Yield coefficient of Butandiol converted from Acetoin after co2 and o2 depletion (g butandiol/g acetoin)

%molare Massen
Acetoin = 88.11; %g/mol
zweidreiButandiol = 90.14; %g/mol
CO2 = 44.01; %g/mol
H2 = 2;%g/mol
O2 = 32;%g/mol

%Gas constants and calculations
T = 303.15; %Temperature [K]
R = 8314.47; %Gas constant[Pa L moL-1 K-1]
P =101325; %Pressure [Pa]
T_regular = 298.15; % K

Hatm_CO2 = 3.4e-2; %Henry constant for CO2 [mol/L.atm]
Hatm_O2 = 1.3e-3; %Henry constant for O2 [mol/L.atm]
Hatm_H2 = 7.8e-4; %Henry constant for H2 [mol/L.atm]

c_co2 = 2400; %constant for temperature dependent henry constant
c_o2 = 1700; %constant for temperature dependent henry constant
c_h2 = 500; %constant for temperature dependent henry constant

Hatm_CO2_op = Hatm_CO2*exp(c_co2*(1/T-1/T_regular)); % henry constant at 30°C
Hatm_O2_op = Hatm_O2*exp(c_o2*(1/T-1/T_regular)); % henry constant at 30°C
Hatm_H2_op = Hatm_H2*exp(c_h2*(1/T-1/T_regular)); % henry constant at 30°C

He_CO2 = Hatm_CO2_op/P; %Henry constant for CO2 [mol/L.Pa]
He_O2 = Hatm_O2_op/P; %Henry constant for O2 [mol/L.Pa]
He_H2 = Hatm_H2_op/P; %Henry constant for H2 [mol/L.Pa]

%Gases initial (percentage) in air
YO2_0=O2_calc(1,1); %initial proportion oxygen in gas (%)
YCO2_0=CO2_calc(1,1); %initial proportion CO2 in gas (%)
YH2_0=H2_calc(1,1); %initial proportion H2 in gas (%)

%Initial Gases concentrations in liquid (mol/L)
 cCO2_L_0 = YCO2_0*P*He_CO2;
 cO2_L_0 = YO2_0*P*He_O2;
 cH2_L_0 = YH2_0*P*He_H2;

% Defines intitial values from data and guessed parameters
ICs = [X(1,1), A(1,1), B(1,1), CO2_calc(1,1),O2_calc(1,1),H2_calc(1,1), cCO2_L_0,cO2_L_0, cH2_L_0]; %Initial conditions

par0 = [mumax, mudmin, mudmax, KLa,K_O2, K_CO2, K_H2, K_B,q_A_d_max,Y_AB,K1_A,Y_BA_r,q_B_upmax,Y_XA,Y_XB,Y_XCO2,Y_XO2,Y_XH2, t_lag q_A_d_max_end, K1_A_end,Y_BA_r_end];

% Lower bounds
lowerBounds = [0.001, 0.0001, 0.001, 130, 0.00001, 0.00001, 0.00001, 0.001, 0, 0.8, 0.001, 0.8, 0, 0.0001, 0.0001, 0.0001, 0.0001, 0.0001, 0 ,0, 0.001, 0.8 ];
% Upper bound
upperBounds = [0.1, 0.01, 0.2, 180, 2, 2, 2, 8, 4, 1.2, 8, 1.2, 4, 40, 40, 10, 10, 10, 200, 4, 8, 1.2];

## Use fmincon

options = optimset('Display', 'Iter', 'MaxIter', 200, 'TolFun', 1e-6, 'TolX', 1e-6);

paropt = fmincon(@objective, par0, [], [], [], [], lowerBounds, upperBounds, [], options, ICs, T1, X, A, B, T2, CO2_calc, O2_calc, H2_calc,cag_pneum_Time_3,cag_pneum_Time_4,cag_pneum_CO2_calc_new,cag_pneum_O2_calc_new,cag_pneum_H2_calc_new);
Results_par = paropt; % Safe estimated parameters

## Final simulation

tspan1 = T1(:,1);
ttspan1 = 0:0.5:max(tspan1 + 4);
tspan2 = T2(:,1);
ttspan2 = 0:0.5:max(tspan2 + 4);

options=odeset('reltol',1E-4,'abstol',1E-4);
[t,c] = ode15s(@DGL,ttspan2,ICs, options, Results_par); %using 23s is for solving stiffness problem

OD_graph = (c(:,1) - 0.0632)/0.6417;
figure('Name','cag(pneum)','NumberTitle','off');
subplot(3,1,1)
plot(tspan1, OD(:,1), 'ob');
hold on
plot(t, OD_graph , '-r', 'LineWidth', 1);
hold off
x=xlabel('Time [h]');
y=ylabel('OD_{600}');
legend('Lab','Model');
x.Color='black';
y.Color='black';
ax = gca;
ax.YColor = 'k';

subplot(3,1,2)
plot(t,c(:,2),'-r',tspan1,A(:,1),'or');
x1=xlabel('Time [h]');
y1=ylabel('Acetoin [g L^-^1]');
legend('Acetoin', 'exp. Data Acetoin','Location','southeast','NumColumns',2);
y1.Color='black';
x1.Color='black';
ax = gca;
ax.YColor = 'k';
hold off

subplot(3,1,3)
plot(t,c(:,3),'-b',tspan1,B(:,1),'ob');
x1=xlabel('Time [h]');
y1=ylabel('Butandiol [g L^-^1]');
legend('Butandiol','exp. Data Butandiol','Location','southeast','NumColumns',2);
x1.Color='black';
y1.Color='black';
ax = gca;
ax.YColor = 'k';
hold off

figure('Name','cag(pneum)','NumberTitle','off');
subplot (3,1,1)
plot(t,c(:,4).*100,'-b',tspan2,CO2_calc(:,1).*100,'og');
legend('CO2','exp. Data CO2','Location','southeast','NumColumns',2);
y1=ylabel('CO2 [%]');
x1=xlabel('Time [h]');
x1.Color='black';
y1.Color='black';
ax = gca;
ax.YColor = 'k';
hold off

subplot (3,1,2)
plot(t,c(:,5).*100,'-b',tspan2,O2_calc(:,1).*100,'og');
legend('O2','exp. Data CO2','Location','southeast','NumColumns',2);
y1=ylabel('O2 [%]');
x1=xlabel('Time [h]');
x1.Color='black';
y1.Color='black';
ax = gca;
ax.YColor = 'k';
hold off

subplot (3,1,3)
plot(t,c(:,6).*100,'-b',tspan2,H2_calc(:,1).*100,'og');
legend('H2','exp. Data CO2','Location','southeast','NumColumns',2);
y1=ylabel('H2 [%]');
x1=xlabel('Time [h]');
x1.Color='black';
y1.Color='black';
ax = gca;
ax.YColor = 'k';
hold off

figure('Name','cag(pneum)','NumberTitle','off');
subplot (3,1,1)
plot(t,c(:,7),'-b');
legend('CO2','Location','southeast','NumColumns',2);
y1=ylabel('CO2 [mole L^-^1]');
x1=xlabel('Time [h]');
x1.Color='black';
y1.Color='black';
ax = gca;
ax.YColor = 'k';
hold off

subplot (3,1,2)
plot(t,c(:,8),'-b');
legend('O2 in liquid','Location','southeast','NumColumns',2);
y1=ylabel('O2 in liquid [mole L^-^1]');
x1=xlabel('Time [h]');
x1.Color='black';
y1.Color='black';
ax = gca;
ax.YColor = 'k';
hold off

subplot (3,1,3)
legend('H2 in liquid','Location','southeast','NumColumns',2);
plot(t,c(:,9),'-b');
y1=ylabel('H2 [mole L^-^1]');
x1=xlabel('Time [h]');
x1.Color='black';
y1.Color='black';
ax = gca;
ax.YColor = 'k';
hold off

%
TT1 = 2 * (T1 + 1);
TT1 = round(TT1);

TT2 = 2 * (T2 +1) ;
TT2 = round(TT2);

lab1 = OD;
lab2 = A;
lab3 = B;
lab4 = CO2_calc;
lab5 = O2_calc;
lab6 = H2_calc;

model1 = OD_graph(TT1 ,1);
model2 = c(TT1,2);
model3 = c(TT1,3);
model4 = c(TT2,4);
model5 = c(TT2,5);
model6 = c(TT2,6);

% Calculate the squared error
SQ1 = sum((lab1 - model1).^2);
SQ2 = sum((lab2 - model2).^2);
SQ3 = sum((lab3 - model3).^2);
SQ4 = sum((lab4 - model4).^2);
SQ5 = sum((lab5 - model5).^2);
SQ6 = sum((lab6 - model6).^2);

%
MSQ1 = sum((lab1 - mean(lab1)).^2);
MSQ2 = sum((lab2 - mean(lab2)).^2);
MSQ3 = sum((lab3 - mean(lab3)).^2);
MSQ4 = sum((lab4 - mean(lab4)).^2);
MSQ5 = sum((lab5 - mean(lab5)).^2);
MSQ6 = sum((lab6 - mean(lab6)).^2);

%
rmse1 = 1 - SQ1/MSQ1;
rmse2 = 1 - SQ2/MSQ2;
rmse3 = 1 - SQ3/MSQ3;
rmse4 = 1 - SQ4/MSQ4;
rmse5 = 1 - SQ5/MSQ5;
rmse6 = 1 - SQ6/MSQ6;

RMSE = [rmse1 rmse2 rmse3 rmse4 rmse5 rmse6 ];
% Display the RMSE
disp('RMSE: ');
disp(RMSE);

out_file = 'Autotrophfit';
xlswrite(out_file,OD_graph(:,1),'Fit1','AI8');%OD
xlswrite(out_file,c(:,2)./Acetoin.*1000,'Fit1','AJ8'); % acetoin mM
xlswrite(out_file,c(:,3)./zweidreiButandiol.*1000,'Fit1','AK8'); % butandiol mM
xlswrite(out_file,c(:,4).*100,'Fit1','AL8'); %CO2gas
xlswrite(out_file,c(:,5).*100,'Fit1','AM8');%O2gas
xlswrite(out_file,c(:,6).*100,'Fit1','AN8');%H2gas
xlswrite(out_file,c(:,7),'Fit1','AO8');%CO2 liquid
xlswrite(out_file,c(:,8),'Fit1','AP8');%O2 liquid
xlswrite(out_file,c(:,9),'Fit1','AQ8');%H2 liquid
xlswrite(out_file,t(:,1),'Fit1','AH8');%Time

xlswrite(out_file,paropt(:,:),'Fit1','H4'); %
xlswrite(out_file,rmse1,'Fit1','A4'); %
xlswrite(out_file,rmse2,'Fit1','B4'); %
xlswrite(out_file,rmse3,'Fit1','C4'); %
xlswrite(out_file,rmse4,'Fit1','D4'); %
xlswrite(out_file,rmse5,'Fit1','E4'); %
xlswrite(out_file,rmse6,'Fit1','F4'); %

## Objective

function J_final=objective(par,ICs,T1, X, A, B, T2,CO2_calc, O2_calc, H2_calc,cag_pneum_Time_3,cag_pneum_Time_4,cag_pneum_CO2_calc_new,cag_pneum_O2_calc_new,cag_pneum_H2_calc_new )

 weight = [1;...% weighting viable cell densitiy
 4;... % weighting Acetoin
 7;... % weighting Butandiol
 950;... % weighting CO2
 180;... % Weighting O2
 12];... % Weighting H2

% Solves the DGL System with starting values
options=odeset('reltol',1E-09,'abstol',1E-09,'maxstep',0.1,'normcontrol','on');

t_all = T1;
tspan = 0:0.1:max(T1)+1;

size_vec = size(T1,1);

[~,c] = ode15s(@DGL,tspan,ICs,options,par);

% Concentration profile over time
c_1 = zeros(size_vec,1);
c_2 = zeros(size_vec,1);
c_3 = zeros(size_vec,1);

[~,LocAllB] = ismembertol(tspan',t_all(1:end,1),0.0001);
[~,LocAllB2] = ismembertol(tspan',t_all(1:end,1),0.0001);
[~,LocAllB3] = ismembertol(tspan',t_all(1:end,1),0.0001);

I = zeros(length(t_all(1:end,1)),3);

for timing = 1:size(t_all(1:end,1),1)

 I(timing,1) = find(timing==LocAllB);
 I(timing,2) = find(timing==LocAllB2);
 I(timing,3) = find(timing==LocAllB3);
end

for j = 1:size(t_all,1)
 c_1(j,:) = c(I(j,1),1);
 c_2(j,:) = c(I(j,2),2);
 c_3(j,:) = c(I(j,3),3);
end

% Solves the DGL System with starting values
options=odeset('reltol',1E-09,'abstol',1E-09,'maxstep',0.1,'normcontrol','on');

t_all_2 = round(cag_pneum_Time_3,1);
t_all_3 = round(cag_pneum_Time_4,1);

tspan_2 = 0:0.1:max(cag_pneum_Time_3)+1;

size_vec_2 = size(cag_pneum_Time_3,1);
size_vec_3 = size(cag_pneum_Time_4,1);

[~,c] = ode15s(@DGL,tspan_2,ICs,options,par);

% Concentration profile over time

c_4 = zeros(size_vec_2,1);
c_5 = zeros(size_vec_3,1);
c_6 = zeros(size_vec_3,1);

[~,LocAllB4] = ismembertol(tspan_2',t_all_2(1:end,1),0.0001);
[~,LocAllB5] = ismembertol(tspan_2',t_all_3(1:end,1),0.0001);
[~,LocAllB6] = ismembertol(tspan_2',t_all_3(1:end,1),0.0001);

I2 = zeros(length(t_all_2(1:end,1)),3);
I3 = zeros(length(t_all_3(1:end,1)),3);

for timing = 1:size(t_all_2(1:end,1),1)

 I2(timing,1) = find(timing==LocAllB4);
end

for j = 1:size(t_all_2,1)
 c_4(j,:) = c(I2(j,1),4);
end

for timing = 1:size(t_all_3(1:end,1),1)

 I3(timing,1) = find(timing==LocAllB5);
 I3(timing,2) = find(timing==LocAllB6);
end

for j = 1:size(t_all_3,1)
 c_5(j,:) = c(I3(j,1),5);
 c_6(j,:) = c(I3(j,2),6);
end

 J_final = sum(weight(1)*sum(abs(c_1(:,1)- X(:,1)).^2)...
 +weight(2)*sum(abs(c_2(:,1)- A(:,1)).^2)...
 +weight(3)*sum(abs(c_3(:,1)- B(:,1)).^2)...
 +weight(4)*sum(abs(c_4(:,1)- cag_pneum_CO2_calc_new(:,1)).^2)...
 +weight(5)*sum(abs(c_5(:,1)- cag_pneum_O2_calc_new(:,1)).^2)...
 +weight(6)*sum(abs(c_6(:,1)- cag_pneum_H2_calc_new(:,1)).^2));

end

## ODEs for Batch reactor

function dcdt= DGL(t,c,par)

%Gas constants and calculations
T = 303.15; %Temperature [K]
R = 8314.47; %Gas constant[Pa L moL-1 K-1]
P =101325; %Pressure [Pa]
T_regular = 298.15; % K

Hatm_CO2 = 3.4e-2; %Henry constant for CO2 [mol/L.atm]
Hatm_O2 = 1.3e-3; %Henry constant for O2 [mol/L.atm]
Hatm_H2 = 7.8e-4; %Henry constant for H2 [mol/L.atm]

c_co2 = 2400; %constant for temperature dependent henry constant
c_o2 = 1700; %constant for temperature dependent henry constant
c_h2 = 500; %constant for temperature dependent henry constant

Hatm_CO2_op = Hatm_CO2*exp(c_co2*(1/T-1/T_regular)); % henry constant at 30°C
Hatm_O2_op = Hatm_O2*exp(c_o2*(1/T-1/T_regular)); % henry constant at 30°C
Hatm_H2_op = Hatm_H2*exp(c_h2*(1/T-1/T_regular)); % henry constant at 30°C

He_CO2 = Hatm_CO2_op/P; %Henry constant for CO2 [mol/L.Pa]
He_O2 = Hatm_O2_op/P; %Henry constant for O2 [mol/L.Pa]
He_H2 = Hatm_H2_op/P; %Henry constant for H2 [mol/L.Pa]

% constant parameters
VL =0.05; %liquid volume [L]
VG = 0.95; % Gas volume [L]

%Renaming variables for easier overview
X=c(1); % g/l
PrA=c(2); % g/l
PrB=c(3); % g/l
YCO2= c(4); % %
YO2 = c(5); % %
YH2 = c(6); % %
cCO2_L = c(7); % moles/l
cO2_L = c(8); % moles/l
cH2_L = c(9); % moles/l

%Parameters after optimization

mumax = par(1);
mudmin = par(2);
mudmax = par(3);
KLa = par(4);
K_O2 = par(5);
K_CO2 = par(6);
K_H2 = par(7);
K_B = par(8);
q_A_d_max = par(9);
Y_AB = par(10);
K1_A = par(11);
Y_BA_r = par(12);
q_B_upmax = par(13);
Y_XA = par(14);
Y_XB = par(15);
Y_XCO2 = par(16);
Y_XO2 = par(17);
Y_XH2 = par(18);
t_lag = par(19);
q_A_d_max_end = par(20);
K1_A_end = par(21);
Y_BA_r_end = par(22);

mu = mumax* (cCO2_L /(K_CO2+cCO2_L))*(cO2_L/(K_O2+cO2_L)) *(cH2_L/(K_H2+cH2_L)) ; %Specific growth rate of biomass (1/h)
mud =mudmin + mudmax *(cCO2_L /(K_CO2+cCO2_L))*(K_O2/(K_O2+cO2_L)) * (K_H2/(K_H2+cH2_L)); %Specific death rate of biomass(1/h)

q_A_P = mu/Y_XA;
q_B = mu/Y_XB;

dcdt(1,1)= (mu-mud)*X ; %dxdt [g biomass/L h]
dcdt(2,1)= (q_A_P) * X ; % dAcetoin/dt [g/L h]
dcdt(3,1)= (q_B)* X ; % dButandiol/dt [g/L h]
dcdt(4,1)= - KLa * VL/VG * R*T/P * (YCO2*He_CO2*P - cCO2_L); % dCO2/dt in gas [%CO2/h]
dcdt(5,1)= - KLa * VL/VG * R*T/P * (YO2*He_O2*P - cO2_L); % dO2/dt in gas [%O2/h]
dcdt(6,1)= - KLa * VL/VG * R*T/P * (YH2*He_H2*P - cH2_L); % dH2/dt in gas [%H2/h]
dcdt(7,1)= KLa*(YCO2*He_CO2*P - cCO2_L)- X*mu/Y_XCO2; % dCO2/dt in liquid [mole/L h]
dcdt(8,1)= KLa*(YO2*He_O2*P - cO2_L)- X*mu/Y_XO2; % dO2/dt in liquid [mole/L h]
dcdt(9,1)= KLa*(YH2*He_H2*P - cH2_L)- X*mu/Y_XH2 ; % dH2/dt in liquid [mole/L h]
end

clc
clear all
close all

 % Data imported from Excel
pBBR_alsSD_Time_1=xlsread('Autotroph.xlsx','pBBR_alsSD','E2:E10'); %h
pBBR_alsSD_OD=xlsread('Autotroph.xlsx','pBBR_alsSD','I2:I10'); %OD600
pBBR_alsSD_X = (0.6417 * pBBR_alsSD_OD + 0.0632); %g/l
pBBR_alsSD_Acetoin=xlsread('Autotroph.xlsx','pBBR_alsSD','AM2:AM10'); %g/l
pBBR_alsSD_Butandiol=xlsread('Autotroph.xlsx','pBBR_alsSD','AN2:AN10'); %g/l
pBBR_alsSD_Time_2=xlsread('Autotroph.xlsx','pBBR_alsSD','B33:B37'); %h
pBBR_alsSD_CO2=xlsread('Autotroph.xlsx','pBBR_alsSD','C33:C37'); %
pBBR_alsSD_CO2_calc = pBBR_alsSD_CO2/100;
pBBR_alsSD_O2=xlsread('Autotroph.xlsx','pBBR_alsSD','G33:G37'); %
pBBR_alsSD_O2_calc = pBBR_alsSD_O2/100;
pBBR_alsSD_H2=xlsread('Autotroph.xlsx','pBBR_alsSD','E33:E37'); %
pBBR_alsSD_H2_calc = pBBR_alsSD_H2/100;

T1 = pBBR_alsSD_Time_1;
OD = pBBR_alsSD_OD;
X = pBBR_alsSD_X;
A = pBBR_alsSD_Acetoin;
B = pBBR_alsSD_Butandiol;
T2 = pBBR_alsSD_Time_2;
CO2_calc = pBBR_alsSD_CO2_calc;
O2_calc = pBBR_alsSD_O2_calc;
H2_calc = pBBR_alsSD_H2_calc;

%Parameters chosen by hand for curve fitting and optimization
mumax = 0.02; %1 % Maximum specific growth rate (1/h)
mudmin = 0.001; %2 %Minimum specific death rate (1/h)
mudmax = 0.02; %3 %Maximum specific death rate (1/h)
KLa = 150; %4 %Mass transfer coefficient for shaking flasks (1/h)
K_O2 = 0.00003125; %5 % (Monod constant) Half-saturation constant for O2 (in g/L) mole/L
K_CO2 = 0.0000227; %6 % (Monod constant) Half-saturation constant for CO2 (in g/L) mole/L
K_H2 = 0.0005; %7 % (Monod constant) Half-saturation constant for H2 (in g/L) mole/L
K_B = 0.005; %8 (Monod constant) Half-saturation constant butandiol (g/l)
q_A_d_max = 0.00001; %9 Maximum specific consumption rate of Acetoin by biomass after the depletion of CO2 (g acetoin/g biomass)
Y_AB = 0.97; %10 Yield coefficient of Acetoin converted back from Butandiol (g acetoin/g Butanediol)
K1_A = 0.005; %11 (Monod constant) Half-saturation constant acetoin during first forward reaction after co2 depletion (g/l)
Y_BA_r = 1.02; %12 Yield coefficient of Butandiol converted from Acetoin (g butandiol/g acetoin)
q_B_upmax = 0.00001; %13 Maximum specific consumption rate of butandiol by biomass after the depletion of CO2 with still oxygen (g butandiol / g biomass)
Y_XA = 0.005; %14 Yield coefficient of biomass produced acetoin (g biomass/g acetoin)
Y_XB = 0.005; %15 Yield coefficient of biomass produced butandiol (g biomass/g butandiol)
Y_XCO2 = 0.005; %16 Yield coefficient of biomass per CO2 (g biomass/mole CO2)
Y_XO2 = 0.005; %17 Yield coefficient of biomass per O2 (g biomass/mole O2)
Y_XH2 = 0.005; %18 Yield coefficient of biomass per H2 (g biomass/mole H2)
t_lag = 15; %19 time term for lag phase and delayed grwoth or production (h)

%molare Massen
Acetoin = 88.11; %g/mol
zweidreiButandiol = 90.14; %g/mol
CO2 = 44.01; %g/mol
H2 = 2;%g/mol
O2 = 32;%g/mol

%Gas constants and calculations
T = 303.15; %Temperature [K]
R = 8314.47; %Gas constant[Pa L moL-1 K-1]
P =101325; %Pressure [Pa]
T_regular = 298.15; % K

Hatm_CO2 = 3.4e-2; %Henry constant for CO2 [mol/L.atm]
Hatm_O2 = 1.3e-3; %Henry constant for O2 [mol/L.atm]
Hatm_H2 = 7.8e-4; %Henry constant for H2 [mol/L.atm]

c_co2 = 2400; %constant for temperature dependent henry constant
c_o2 = 1700; %constant for temperature dependent henry constant
c_h2 = 500; %constant for temperature dependent henry constant

Hatm_CO2_op = Hatm_CO2*exp(c_co2*(1/T-1/T_regular)); % henry constant at 30°C
Hatm_O2_op = Hatm_O2*exp(c_o2*(1/T-1/T_regular)); % henry constant at 30°C
Hatm_H2_op = Hatm_H2*exp(c_h2*(1/T-1/T_regular)); % henry constant at 30°C

He_CO2 = Hatm_CO2_op/P; %Henry constant for CO2 [mol/L.Pa]
He_O2 = Hatm_O2_op/P; %Henry constant for O2 [mol/L.Pa]
He_H2 = Hatm_H2_op/P; %Henry constant for H2 [mol/L.Pa]

%Gases initial (percentage) in air
YO2_0=0.15; %initial proportion oxygen in gas (%)
YCO2_0=0.05; %initial proportion CO2 in gas (%)
YH2_0=0.8; %initial proportion H2 in gas (%)

%Initial Gases concentrations in liquid (mol/L)
 cCO2_L_0 = YCO2_0*P*He_CO2;
 cO2_L_0 = YO2_0*P*He_O2;
 cH2_L_0 = YH2_0*P*He_H2;

% Defines intitial values from data and guessed parameters
ICs = [X(1,1), A(1,1), B(1,1), CO2_calc(1,1),O2_calc(1,1),H2_calc(1,1), cCO2_L_0,cO2_L_0, cH2_L_0]; %Initial conditions

par0 = [mumax, mudmin, mudmax, KLa,K_O2, K_CO2, K_H2, K_B,q_A_d_max,Y_AB,K1_A,Y_BA_r,q_B_upmax,Y_XA,Y_XB,Y_XCO2,Y_XO2,Y_XH2, t_lag];

% Lower bounds
lowerBounds = [0.001, 0, 0.001, 130, 0.00001, 0.00001, 0.00001, 0.001, 0, 0.8, 0.001, 0.8, 0, 0.0001, 0.0001, 0.0001, 0.0001, 0.0001,0 ];
% Upper bound
upperBounds = [0.2, 0.01, 0.2, 180, 10, 10, 10, 8, 4, 1.2, 8, 1.2, 4, 40, 40, 10, 10, 10, 200];

## Use fmincon

options = optimset('Display', 'Iter', 'MaxIter', 200, 'TolFun', 1e-6, 'TolX', 1e-6);

paropt = fmincon(@objective, par0, [], [], [], [], lowerBounds, upperBounds, [], options, ICs, T1, X, A, B, T2, CO2_calc, O2_calc, H2_calc);
Results_par = paropt; % Safe estimated parameters

## Final simulation

tspan1 = T1(:,1);
ttspan1 = 0:0.5:max(tspan1 + 4);
tspan2 = T2(:,1);
ttspan2 = 0:0.5:max(tspan2 + 4);

options=odeset('reltol',1E-4,'abstol',1E-4);
[t,c] = ode15s(@DGL,ttspan2,ICs, options, Results_par); %using 23s is for solving stiffness problem

OD_graph = (c(:,1) - 0.0632)/0.6417;
figure('Name','pBBR_alsSD','NumberTitle','off');
subplot(3,1,1)
plot(tspan1, OD(:,1), 'ob');
hold on
plot(t, OD_graph , '-r', 'LineWidth', 1);
hold off
x=xlabel('Time [h]');
y=ylabel('OD_{600}');
legend('Lab','Model');
x.Color='black';
y.Color='black';
ax = gca;
ax.YColor = 'k';

subplot(3,1,2)
plot(t,c(:,2),'-r',tspan1,A(:,1),'or');
x1=xlabel('Time [h]');
y1=ylabel('Acetoin [g L^-^1]');
legend('Acetoin', 'exp. Data Acetoin','Location','southeast','NumColumns',2);
y1.Color='black';
x1.Color='black';
ax = gca;
ax.YColor = 'k';
hold off

subplot(3,1,3)
plot(t,c(:,3),'-b',tspan1,B(:,1),'ob');
x1=xlabel('Time [h]');
y1=ylabel('Butandiol [g L^-^1]');
legend('Butandiol','exp. Data Butandiol','Location','southeast','NumColumns',2);
x1.Color='black';
y1.Color='black';
ax = gca;
ax.YColor = 'k';
hold off

figure('Name','pBBR_alsSD','NumberTitle','off');
subplot (3,1,1)
plot(t,c(:,4).*100,'-b',tspan2,CO2_calc(:,1).*100,'og');
legend('CO2','exp. Data CO2','Location','southeast','NumColumns',2);
y1=ylabel('CO2 [%]');
x1=xlabel('Time [h]');
x1.Color='black';
y1.Color='black';
ax = gca;
ax.YColor = 'k';
hold off

subplot (3,1,2)
plot(t,c(:,5).*100,'-b',tspan2,O2_calc(:,1).*100,'og');
legend('O2','exp. Data CO2','Location','southeast','NumColumns',2);
y1=ylabel('O2 [%]');
x1=xlabel('Time [h]');
x1.Color='black';
y1.Color='black';
ax = gca;
ax.YColor = 'k';
hold off

subplot (3,1,3)
plot(t,c(:,6).*100,'-b',tspan2,H2_calc(:,1).*100,'og');
legend('H2','exp. Data CO2','Location','southeast','NumColumns',2);
y1=ylabel('H2 [%]');
x1=xlabel('Time [h]');
x1.Color='black';
y1.Color='black';
ax = gca;
ax.YColor = 'k';
hold off

figure('Name','pBBR_alsSD','NumberTitle','off');
subplot (3,1,1)
plot(t,c(:,7),'-b');
legend('CO2','Location','southeast','NumColumns',2);
y1=ylabel('CO2 [mole L^-^1]');
x1=xlabel('Time [h]');
x1.Color='black';
y1.Color='black';
ax = gca;
ax.YColor = 'k';
hold off

subplot (3,1,2)
plot(t,c(:,8),'-b');
legend('O2 in liquid','Location','southeast','NumColumns',2);
y1=ylabel('O2 in liquid [mole L^-^1]');
x1=xlabel('Time [h]');
x1.Color='black';
y1.Color='black';
ax = gca;
ax.YColor = 'k';
hold off

subplot (3,1,3)
legend('H2 in liquid','Location','southeast','NumColumns',2);
plot(t,c(:,9),'-b');
y1=ylabel('H2 [mole L^-^1]');
x1=xlabel('Time [h]');
x1.Color='black';
y1.Color='black';
ax = gca;
ax.YColor = 'k';
hold off

%
TT1 = 2 * (T1 + 1);
TT1 = round(TT1);

TT2 = 2 * (T2 +1) ;
TT2 = round(TT2);

lab1 = OD;
lab2 = A;
lab3 = B;
lab4 = CO2_calc;
lab5 = O2_calc;
lab6 = H2_calc;

model1 = OD_graph(TT1 ,1);
model2 = c(TT1,2);
model3 = c(TT1,3);
model4 = c(TT2,4);
model5 = c(TT2,5);
model6 = c(TT2,6);

% Calculate the squared error
SQ1 = sum((lab1 - model1).^2);
SQ2 = sum((lab2 - model2).^2);
SQ3 = sum((lab3 - model3).^2);
SQ4 = sum((lab4 - model4).^2);
SQ5 = sum((lab5 - model5).^2);
SQ6 = sum((lab6 - model6).^2);

%
MSQ1 = sum((lab1 - mean(lab1)).^2);
MSQ2 = sum((lab2 - mean(lab2)).^2);
MSQ3 = sum((lab3 - mean(lab3)).^2);
MSQ4 = sum((lab4 - mean(lab4)).^2);
MSQ5 = sum((lab5 - mean(lab5)).^2);
MSQ6 = sum((lab6 - mean(lab6)).^2);

%
rmse1 = 1 - SQ1/MSQ1;
rmse2 = 1 - SQ2/MSQ2;
rmse3 = 1 - SQ3/MSQ3;
rmse4 = 1 - SQ4/MSQ4;
rmse5 = 1 - SQ5/MSQ5;
rmse6 = 1 - SQ6/MSQ6;

RMSE = [rmse1 rmse2 rmse3 rmse4 rmse5 rmse6 ];
% Display the RMSE
disp('RMSE: ');
disp(RMSE);

%molare Massen
Acetoin = 88.11; %g/mol
zweidreiButandiol = 90.14; %g/mol

out_file = 'Autotrophfit';
xlswrite(out_file,OD_graph(:,1),'Fit1','AT8');%OD
xlswrite(out_file,c(:,2)./Acetoin.*1000,'Fit1','AU8'); % acetoin mM
xlswrite(out_file,c(:,3)./zweidreiButandiol.*1000,'Fit1','AV8'); % butandiol mM
xlswrite(out_file,c(:,4).*100,'Fit1','AW8'); %CO2gas
xlswrite(out_file,c(:,5).*100,'Fit1','AX8');%O2gas
xlswrite(out_file,c(:,6).*100,'Fit1','AY8');%H2gas
xlswrite(out_file,c(:,7),'Fit1','AZ8');%CO2 liquid
xlswrite(out_file,c(:,8),'Fit1','BA8');%O2 liquid
xlswrite(out_file,c(:,9),'Fit1','BB8');%H2 liquid
xlswrite(out_file,t(:,1),'Fit1','AS8');%Time

xlswrite(out_file,paropt(:,:),'Fit1','H5'); %
xlswrite(out_file,rmse1,'Fit1','A5'); %
xlswrite(out_file,rmse2,'Fit1','B5'); %
xlswrite(out_file,rmse3,'Fit1','C5'); %
xlswrite(out_file,rmse4,'Fit1','D5'); %
xlswrite(out_file,rmse5,'Fit1','E5'); %
xlswrite(out_file,rmse6,'Fit1','F5'); %

## Objective

function J_final=objective(par,ICs,T1, X, A, B, T2,CO2_calc, O2_calc, H2_calc)

weight = [1;...% weighting viable cell densitiy
 5;... % weighting Acetoin
 8;... % weighting Butandiol
 500;... % weighting CO2
 60;... % Weighting O2
 6];... % Weighting H2

% Solves the DGL System with starting values
options=odeset('reltol',1E-09,'abstol',1E-09,'maxstep',0.1,'normcontrol','on');

t_all = T1;
tspan = 0:0.1:max(T1)+1;

size_vec = size(T1,1);

[~,c] = ode15s(@DGL,tspan,ICs,options,par);

% Concentration profile over time
c_1 = zeros(size_vec,1);
c_2 = zeros(size_vec,1);
c_3 = zeros(size_vec,1);

[~,LocAllB] = ismembertol(tspan',t_all(1:end,1),0.0001);
[~,LocAllB2] = ismembertol(tspan',t_all(1:end,1),0.0001);
[~,LocAllB3] = ismembertol(tspan',t_all(1:end,1),0.0001);

I = zeros(length(t_all(1:end,1)),3);

for timing = 1:size(t_all(1:end,1),1)

 I(timing,1) = find(timing==LocAllB);
 I(timing,2) = find(timing==LocAllB2);
 I(timing,3) = find(timing==LocAllB3);
end

for j = 1:size(t_all,1)
 c_1(j,:) = c(I(j,1),1);
 c_2(j,:) = c(I(j,2),2);
 c_3(j,:) = c(I(j,3),3);
end

% Solves the DGL System with starting values
options=odeset('reltol',1E-09,'abstol',1E-09,'maxstep',0.1,'normcontrol','on');

t_all_2 = round(T2,1);
tspan_2 = 0:0.1:max(T2)+1;

size_vec_2 = size(T2,1);

[~,c] = ode15s(@DGL,tspan_2,ICs,options,par);

% Concentration profile over time
c_4 = zeros(size_vec_2,1);
c_5 = zeros(size_vec_2,1);
c_6 = zeros(size_vec_2,1);

[~,LocAllB4] = ismembertol(tspan_2',t_all_2(1:end,1),0.0001);
[~,LocAllB5] = ismembertol(tspan_2',t_all_2(1:end,1),0.0001);
[~,LocAllB6] = ismembertol(tspan_2',t_all_2(1:end,1),0.0001);

I2 = zeros(length(t_all_2(1:end,1)),3);

for timing = 1:size(t_all_2(1:end,1),1)

 I2(timing,1) = find(timing==LocAllB4);
 I2(timing,2) = find(timing==LocAllB5);
 I2(timing,3) = find(timing==LocAllB6);
end

for j = 1:size(t_all_2,1)
 c_4(j,:) = c(I2(j,1),4);
 c_5(j,:) = c(I2(j,2),5);
 c_6(j,:) = c(I2(j,3),6);
end

 J_final = sum(weight(1)*sum(abs(c_1(:,1)- X(:,1)).^2)...
 +weight(2)*sum(abs(c_2(:,1)- A(:,1)).^2)...
 +weight(3)*sum(abs(c_3(:,1)- B(:,1)).^2)...
 +weight(4)*sum(abs(c_4(:,1)- CO2_calc(:,1)).^2)...
 +weight(5)*sum(abs(c_5(:,1)- O2_calc(:,1)).^2)...
 +weight(6)*sum(abs(c_6(:,1)- H2_calc(:,1)).^2));

end

## ODEs for Batch reactor

function dcdt= DGL(t,c,par)

%Gas constants and calculations
T = 303.15; %Temperature [K]
R = 8314.47; %Gas constant[Pa L moL-1 K-1]
P =101325; %Pressure [Pa]
T_regular = 298.15; % K

Hatm_CO2 = 3.4e-2; %Henry constant for CO2 [mol/L.atm]
Hatm_O2 = 1.3e-3; %Henry constant for O2 [mol/L.atm]
Hatm_H2 = 7.8e-4; %Henry constant for H2 [mol/L.atm]

c_co2 = 2400; %constant for temperature dependent henry constant
c_o2 = 1700; %constant for temperature dependent henry constant
c_h2 = 500; %constant for temperature dependent henry constant

Hatm_CO2_op = Hatm_CO2*exp(c_co2*(1/T-1/T_regular)); % henry constant at 30°C
Hatm_O2_op = Hatm_O2*exp(c_o2*(1/T-1/T_regular)); % henry constant at 30°C
Hatm_H2_op = Hatm_H2*exp(c_h2*(1/T-1/T_regular)); % henry constant at 30°C

He_CO2 = Hatm_CO2_op/P; %Henry constant for CO2 [mol/L.Pa]
He_O2 = Hatm_O2_op/P; %Henry constant for O2 [mol/L.Pa]
He_H2 = Hatm_H2_op/P; %Henry constant for H2 [mol/L.Pa]

% constant parameters
VL =0.05; %liquid volume [L]
VG = 0.95; % Gas volume [L]

%Renaming variables for easier overview
X=c(1); % g/l
PrA=c(2); % g/l
PrB=c(3); % g/l
YCO2= c(4); % %
YO2 = c(5); % %
YH2 = c(6); % %
cCO2_L = c(7); % moles/l
cO2_L = c(8); % moles/l
cH2_L = c(9); % moles/l

%Parameters after optimization
mumax = par(1);
mudmin = par(2);
mudmax = par(3);
KLa = par(4);
K_O2 = par(5);
K_CO2 = par(6);
K_H2 = par(7);
K_B = par(8);
q_A_d_max = par(9);
Y_AB = par(10);
K1_A = par(11);
Y_BA_r = par(12);
q_B_upmax = par(13);
Y_XA = par(14);
Y_XB = par(15);
Y_XCO2 = par(16);
Y_XO2 = par(17);
Y_XH2 = par(18);
t_lag = par(19);

mu = mumax* (cCO2_L /(K_CO2+cCO2_L))*(cO2_L/(K_O2+cO2_L)) *(cH2_L/(K_H2+cH2_L)) ; %Specific growth rate of biomass (1/h)
mud =mudmin + mudmax *(cCO2_L /(K_CO2+cCO2_L))*(K_O2/(K_O2+cO2_L)) * (K_H2/(K_H2+cH2_L)); %Specific death rate of biomass(1/h)

q_A_P = mu/Y_XA;
q_B = mu/Y_XB*(1-exp(-t/t_lag));

dcdt(1,1)= (mu-mud)*X ; %dxdt [g biomass/L h]
dcdt(2,1)= (q_A_P) * X ; % dAcetoin/dt [g/L h]
dcdt(3,1)= (q_B)* X ; % dButandiol/dt [g/L h]
dcdt(4,1)= - KLa * VL/VG * R*T/P * (YCO2*He_CO2*P - cCO2_L); % dCO2/dt in gas [%CO2/h]
dcdt(5,1)= - KLa * VL/VG * R*T/P * (YO2*He_O2*P - cO2_L); % dO2/dt in gas [%O2/h]
dcdt(6,1)= - KLa * VL/VG * R*T/P * (YH2*He_H2*P - cH2_L); % dH2/dt in gas [%H2/h]
dcdt(7,1)= KLa*(YCO2*He_CO2*P - cCO2_L)- X*mu/Y_XCO2; % dCO2/dt in liquid [mole/L h]
dcdt(8,1)= KLa*(YO2*He_O2*P - cO2_L)- X*mu/Y_XO2; % dO2/dt in liquid [mole/L h]
dcdt(9,1)= KLa*(YH2*He_H2*P - cH2_L)- X*mu/Y_XH2 ; % dH2/dt in liquid [mole/L h]

if t>=92.5
mu = mumax* (cCO2_L /(K_CO2+cCO2_L))*(cO2_L/(K_O2+cO2_L)) *(cH2_L/(K_H2+cH2_L)) ; %Specific growth rate of biomass (1/h)

mu1= mumax*(cO2_L/(K_O2+cO2_L)) *(cH2_L/(K_H2+cH2_L)) ; %Specific growth rate of biomass (1/h)
mud1 =mudmin + mudmax *(K_O2/(K_O2+cO2_L)) * (K_H2/(K_H2+cH2_L)); %Specific death rate of biomass(1/h)

q_A_P = mu/Y_XA;
q_B = mu/Y_XB*(1-exp(-t/t_lag));
q_A_d = q_A_d_max * PrA/(PrA + K1_A) ;
q_A_d_r = q_A_d * Y_BA_r;

dcdt(1,1)= (mu1-mud1)*X ; %dxdt [g biomass/L h]
dcdt(2,1)= (q_A_P-q_A_d) * X ; % dAcetoin/dt [g/L h]
dcdt(3,1)= (q_B+q_A_d_r)* X ; % dButandiol/dt [g/L h
dcdt(4,1)= - KLa * VL/VG * R*T/P * (YCO2*He_CO2*P - cCO2_L); % dCO2/dt in gas [%CO2/h]
dcdt(5,1)= - KLa * VL/VG * R*T/P * (YO2*He_O2*P - cO2_L); % dO2/dt in gas [%O2/h]
dcdt(6,1)= - KLa * VL/VG * R*T/P * (YH2*He_H2*P - cH2_L); % dH2/dt in gas [%H2/h]
dcdt(7,1)= KLa*(YCO2*He_CO2*P - cCO2_L)- X*mu/Y_XCO2; % dCO2/dt in liquid [mole/L h]
dcdt(8,1)= KLa*(YO2*He_O2*P - cO2_L)- X*mu1/Y_XO2; % dO2/dt in liquid [mole/L h]
dcdt(9,1)= KLa*(YH2*He_H2*P - cH2_L)- X*mu1/Y_XH2 ; % dH2/dt in liquid [mole/L h]
end
end

[*Published with MATLAB® R2020b*](https://www.mathworks.com/products/matlab)
